# Finely-Tuned Bis(imino)pyridylcobalt Complexes Enhance Ethylene Polymerization: The Role of Bulky and Halogen Substituents

**DOI:** 10.3390/molecules30040859

**Published:** 2025-02-13

**Authors:** Elizabeth Ogbe, Yanping Ma, Yizhou Wang, Jiahao Gao, Yang Sun, Wen-Hua Sun

**Affiliations:** 1 Key Laboratory of Engineering Plastics and Beijing National Laboratory for Molecular Science, Institute of Chemistry, Chinese Academy of Sciences, Beijing 100190, China; oelizabeth@iccas.ac.cn (E.O.); wangyizhou13@iccas.ac.cn (Y.W.); gaojh514@iccas.ac.cn (J.G.); sy0471103@iccas.ac.cn (Y.S.); 2 CAS Research/Education Center for Excellence in Molecular Sciences and International School, University of Chinese Academy of Sciences, Beijing 100049, China

**Keywords:** unsymmetric bis(imino)pyridylcobalt complex, ethylene polymerization, high activity and thermal stability, highly linear and vinyl-terminal polyethylene

## Abstract

The bis(imino)pyridylcobalt complexes have been finely tuned through using the aniline derivative bearing a *meta*-chloro substituent, besides its *ortho*- and *para*-di(4-fluorophenyl)methyl and *ortho*-methyl substituents for the series of 2-[1-(3-chloro-4,6-bis((di(4-fluorophenyl)methyl)-2-methylphenylimino)ethyl]-6-[1-(arylimino)ethyl]pyridylcobalt(II) chlorides (2,6-Me_2_Ph, **Co1**; 2,6-Et_2_Ph, **Co2**; 2,6-*i*Pr_2_Ph, **Co3**; 2,4,6-Me_3_Ph, **Co4**; and 2,6-Et_2_-4-MePh, **Co5**). The compounds were characterized using elemental analysis, ^1^H/^13^C NMR, FT-IR spectroscopy, and the single-crystal X-ray diffraction used in confirming the molecular structures of **Co1, Co2, Co4,** and **Co5**. The newly synthesized precatalysts, maintaining steric influences with the addition of an electron-withdrawing *meta*-chloro group, achieved higher activities along with better thermal stability, and controlled molecular weights of polyethylenes obtained. Upon activation with either MAO or MMAO, all catalysts exhibited remarkable activity for ethylene polymerization, for example, 9.2 × 10^6^ g mol^−1^ h^−1^ by **Co1** at 70 °C with 30 min and 18.0 × 10^6^ g mol^−1^ h^−1^ by **Co4** with the first 5 min. **Co4** demonstrated exceptionally thermal stability with the peak activity of 8.9 × 10^6^ g mol^−1^ h^−1^ at 70 °C and slightly decreased to 7.2 × 10^6^ g mol^−1^ h^−1^ at 80 °C, and even maintained an activity of 1.6 × 10^6^ g mol^−1^ h^−1^ at 100 °C. More importantly, all resultant polyethylenes were characterized as having vinyl-terminal and high-linear feature along with narrow dispersity; the molecular weights could be adapted in the ranges from 6.4 to 50.0 kg mol^−1^. In comparison with previous cobalt analogs, the current system performed better thermal stability and polymerization efficiency. Therefore, such robust complex catalysts are potentially considered for the polyethylene industry.

## 1. Introduction

Polyethylene is one of the most widely used synthetic polymers, valued for its cost-effectiveness, ease of processing, and recyclability [1]. It serves as a key material in a variety of industries, ranging from packaging to automotive, due to its favorable mechanical properties, chemical resistance, and versatility [2]. The sustainability of polyethylene is closely tied to its hydrocarbon structure derived from natural oils, which can also serve as an energy source [3]. The polymerization of ethylene is central to the production of polyethylene [4], and significant advancements have been made in improving the efficiency and control of this process primarily through the development of highly effective catalysts [5,6,7,8,9,10].

**Scheme 1:**
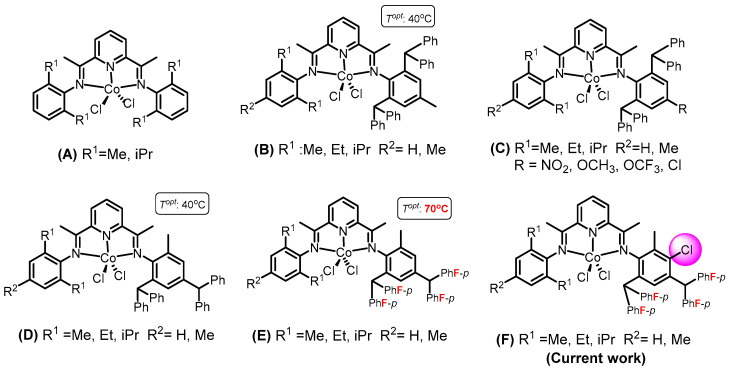
Structural modifications in bis(imino)pyridylcobalt precatalysts (**A**–**E**), along with the precatalyst developed in this work (**F**).

A promising strategy for enhancing catalytic performance in ethylene polymerization is the incorporation of bulky groups into the ligands of metal catalysts [11,12,13,14,15]. These bulky substituents introduce steric hindrance, which can significantly influence the catalytic activity, polymerization kinetics, and polymer properties [16,17]. Such modification can improve catalyst stability and allow for better control over polymer molecular weights, making them essential in the design of next-generation catalysts. Common bulky groups [7], such as dibenzhydryl [12], anthracenyl, and dibenzosuberyl [18] groups, have been shown to significantly improve the activity and thermal stability of catalysts, particularly those based on late-transition metals. Bis(imino)pyridyl cobalt precatalysts (**A**, Scheme 1) firstly discovered in 1998 [19,20,21] have demonstrated remarkable performance in producing highly linear polyethylene, with their activity and thermal stability significantly enhanced by the strategic incorporation of bulky substituents. Particularly, the synthesis of cobalt precatalysts featuring unsymmetrical ligands (**B**, Scheme 1) [12] achieved by introducing the bulky group dibenzhydryl into the two *ortho* positions of one of the N-aryl groups, forming the 2-[1-(2,6-dibenzhydrylphenylimino)ethyl]-6-[1-(arylimino)ethyl] pyridylcobalt precatalyst, has been shown to enhance catalytic activity in ethylene polymerization. This resulted in a peak activity of 9.87 × 10^6^ g mol^−1^ h^−1^. The steric hindrance from these *ortho*-bulky groups protects the catalytic active sites, leading to improved activity and thermal stability. Meanwhile, electronic effects were explored by varying the substituents at the *para* position of the N-aryl ring in the catalysts (**C**, Scheme 1) [12,22,23,24]. A high activity was observed when the *para* position is occupied by a methyl group [12], while good thermal stability was achieved with the methoxy group at this site [21]. These findings demonstrate that both bulky substituents and electronic groups play a crucial role in tuning catalyst performance and controlling the polymerization process. In a previous study by our group, we further optimized the catalysts by shifting the dibenzhydryl moieties from one of the *ortho* positions to the *para* position (**D**, Scheme 1) [25], resulting in a significant increase in activity (18.05 × 10^6^ g mol^−1^ h^−1^). All these catalyst systems produced highly linear polyethylene and narrow polydispersity indices (PDIs), characteristic of single-site active species.

Another significant advancement in polyethylene catalyst design is the introduction of halogen groups, such as fluorine and chlorine, which have been shown to further enhance the performance of ethylene polymerization catalysts [26]. These halogen groups were typically introduced into the *ortho* [27,28] and *para* [29] positions of the N-aryl groups or the *para* position of the bulky group [30,31]. The inclusion of halogen substituents not only improves the thermal stability of the catalysts, but also plays a crucial role in controlling the molecular weight distribution of the resulting polymer [31]. For instance, when we introduced the fluoride group into the *para* position of the bulky group in structure **D**, resulting in catalyst **E** in Scheme 1 [30], the optimal polymerization temperature was found to be 70 °C towards ethylene polymerization, a notable improvement over catalysts **B** and **D**, both of which exhibited an optimal temperature of 40 °C. The halogen groups are known to enhance polymerization efficiency by modifying the electronic properties of the metal center. By fine-tuning the steric and electronic properties of the catalyst, these modifications can enable a more controlled polymerization process. As a result, catalysts with halogen substitutions exhibit higher thermal stability, more consistent polymerization behavior, and better control over the molecular weight and polydispersity of the polymer produced.

In this study, we synthesized a series of unsymmetrical bis(arylimino)pyridine-Co (II) complexes by introducing the fluoride group at the *para* positions of the two bulky dibenzhydryl groups attached to the *ortho* and *para* positions of one of the N-aryl groups, as well as a chloride group at the *meta* position of the N-aryl group (**F**, Scheme 1). The rationale for this design was to explore the synergistic effects of bulky and halogen-substituted groups on the catalytic performance of the cobalt complex in ethylene polymerization. Specifically, we aimed to build upon the strong performance of catalyst **E**, particularly its good thermal stability, which was attributed to the *ortho*- and *para*-substituted 4-fluorodibenzyl groups that increase steric hindrance around the metal center. We hypothesized that adding an electron-withdrawing *meta*-chloro group would further modify the Lewis acidity of the cobalt metal center, enhancing its electrophilic character. This modification was expected to promote the polymerization process by improving the catalyst’s reactivity. By combining these steric and electronic effects, we anticipated an overall enhancement in catalytic activity and thermal stability, and control over polymer properties like molecular weights. To evaluate the catalytic properties of these newly designed complexes, we activated these cobalt complexes with methyl aluminoxane (MAO) or modified-methyl aluminoxane (MMAO) as co-catalyst. A detailed study was conducted under various conditions, including the Al/Co ratio, temperature, ethylene pressure, and reaction time, to thoroughly investigate the influence of these factors on the catalyst’s performance. Our study systematically explored how electronic and steric variations influenced the catalyst’s structure and polymerization capabilities. Additionally, we compared the catalytic performance of these newly cobalt catalysts with earlier versions of similar catalysts, highlighting the impact of halogen and bulky group modifications on the overall polymerization behavior. The results underscore the potential of this strategy for designing highly active, thermally stable catalysts with precise control over polymer molecular weight distribution, an important characteristic for industrial-scale polyethylene production.

## 2. Results and Discussion

### 2.1. Synthesis and Characterizations of Ligands and Cobalt Complexes

**Scheme 2:**
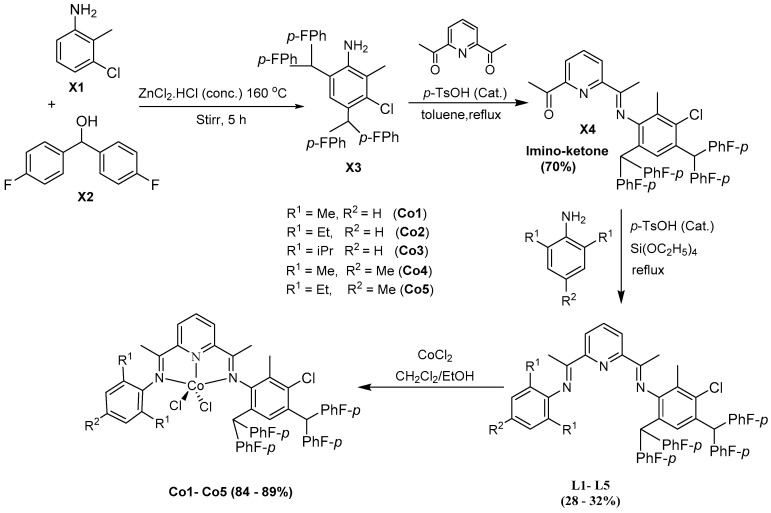
The synthesis route of ligands and their corresponding cobalt complexes.

A series of 2,6-bis(arylimino)pyridine derivatives with an asymmetric framework were synthesized through a multi-step procedure with good overall yields. The synthesized compounds include 2-[1-(3-chloro-4,6-bis((di(4-fluorophenyl)methyl)-2-methyl-phenylimino)-ethyl]-6-(arylimino)ethyl] pyridine, with varying aryl groups: 2,6-Me_2_Ph (**L1**), 2,6-Et_2_Ph (**L2**), 2,6-iPr_2_Ph (**L3**), 2,4,6-Me_3_Ph (**L4**), and 2,6-Et_2_-4-MePh (**L5**)**,** as shown in (Scheme 2).

The synthesis began with the preparation of the bulky 4,6-bis(bis(4-fluorophenyl)-methyl)-3-chloro-2-methylaniline (**X3**) using methods described in the previous literature [30,31,32]. The synthesis commenced with the reaction of 3-chloro-2-methylaniline (**X1**) with bis(4-fluorophenyl)methanol (**X2**) to afford the target bulky aniline **X3**. Next, the condensation of 2,6-diacetylpyridine with one equivalent of **X3** resulted in the primary formation of the imine-ketone 2-acetyl-6-[1-(3-chloro-4,6-bis((di(4-fluorophenyl)methyl)-2-methylphenylimino)ethyl]pyridine (**X4**). The imine-ketone intermediate **X4** was treated with different anilines, under acidic conditions, leading to the formation of ligands **L1** to **L5**. Subsequently, **L1** to **L5** were reacted with anhydrous cobalt (II) chloride in a solvent mixture of ethanol and dichloromethane, producing 2-[1-(3-chloro-4,6-bis((di(4-fluorophenyl)methyl)-2- methylphenylimino)-ethyl]-6-[1(arylimino)ethyl]pyridylcobalt(II) chlorides: (aryl = 2,6-Me_2_Ph **Co1**, 2,6-Et_2_Ph **Co2**, 2,6-iPr_2_Ph **Co3**, 2,4,6-Me_3_Ph **Co4**, and 2,6-Et_2_-4-MePh **Co5** in yields of 84–89% (as shown in Scheme 2).

**Figure 1:**
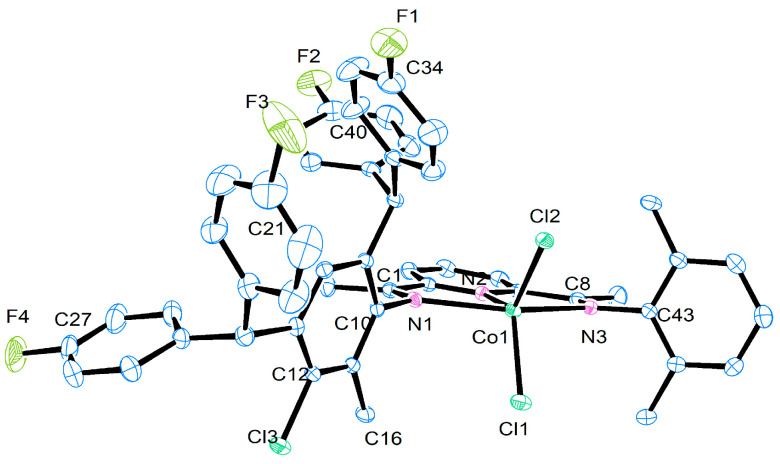
ORTEP representation of **Co1** with thermal ellipsoids illustrated at a 30% probability level, and all hydrogen atoms are omitted for clarity.

**Figure 2:**
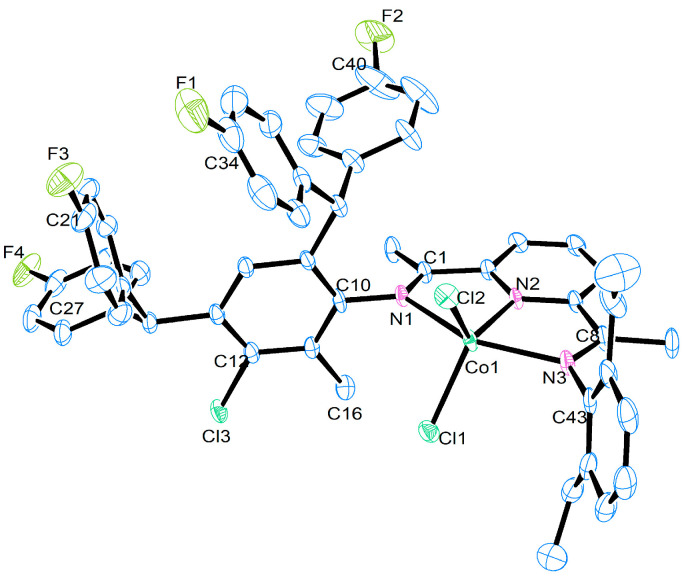
ORTEP representation of **Co2** with thermal ellipsoids illustrated at a 30% probability level, and all hydrogen atoms are omitted for clarity.

**Figure 3:**
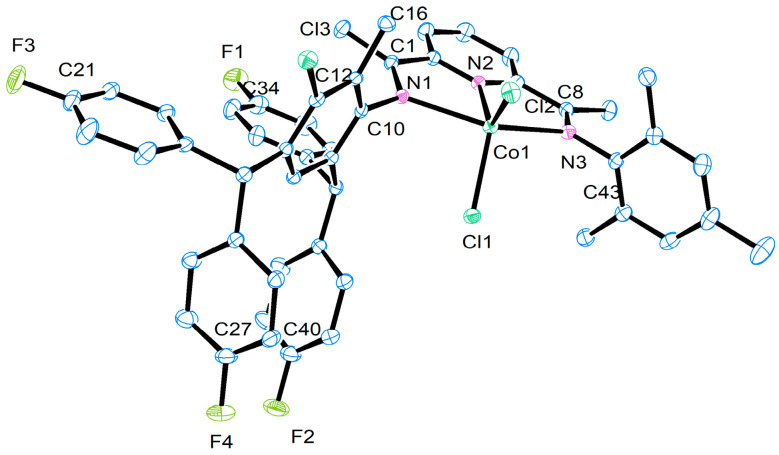
ORTEP representation of **Co4** with thermal ellipsoids illustrated at a 30% probability level, and all hydrogen atoms are omitted for clarity.

**Figure 4:**
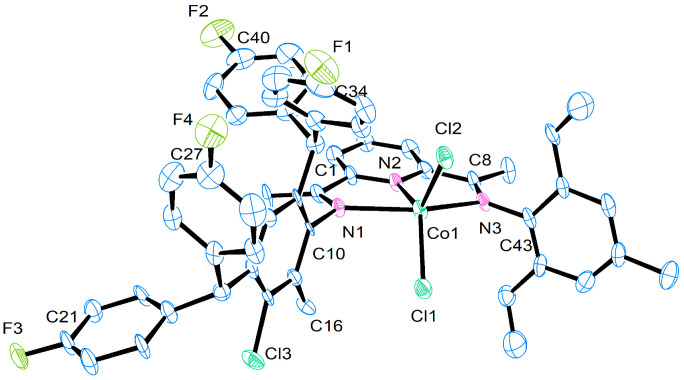
ORTEP representation of **Co5** with thermal ellipsoids illustrated at a 30% probability level, and all hydrogen atoms are omitted for clarity.

The obtained compounds **X3**, **X4,** and ligands **L1**–**L5** were structurally characterized using ^1^H, ^13^C NMR, and/or elemental analysis (Section 3 and Figures S1 and S2). ^19^F NMR spectroscopy was performed on L1–L5 to investigate the fluorine environments. As illustrated in Figure S3, the free ligand spectra displayed signals with chemical shifts around δ −116 ppm. There were no significant differences in the chemical shift positions of these fluoride substituents, as they are attached to the *para* position of the bulky dibenzhydryl group, and are not influenced much by changes in the other N-aryl moiety. The cobalt complexes (**Co1** to **Co5**) were characterized using elemental analysis techniques and FT-IR spectroscopy (Figure S4). **Co1, Co2, Co4,** and **Co5** were further subjected to single-crystal X-ray diffraction for structural elucidation. Single crystals of **Co1, Co2, Co4,** and **Co5** used for X-ray investigations were grown by gradually diffusing n-heptane into a solution of dichloromethane containing the respective complex. The perspectives of crystal structures are shown in Figure 1, Figure 2, Figure 3 and Figure 4, with selected bond lengths and angles summarized in Table 1, while full crystallographic details are available in Table S1. These crystal structures of **Co1**, **Co2, Co4,** and **Co5** exhibited similar conformations and will be collectively described. Each complex structure features a solitary cobalt center coordinated by an N^N^N-chelating bis(arylimino)pyridine and two chloride ions, forming a distorted square pyramid geometry. To quantify this distortion, the geometric tau value (τ5) was calculated using the equation τ5 = (β – α)/60, where β represents the largest angle and α represents the second largest angle in the coordination sphere. The value of τ5 for **Co5** is 0.14 which indicates a nearly perfect square pyramid, while a value of one suggests a perfect trigonal bipyramid. For **Co4, Co2,** and **Co1**, τ5 values were 0.41, 0.45, and 0.41, respectively, indicating some deviation from a perfect square pyramid. The bond lengths between cobalt and nitrogen were analyzed and it was found that the Co(1)-N(2) bond lengths were consistently shorter than those Co(1)-N(1) and Co(1)-N(3), indicating a stronger coordination between cobalt and the central nitrogen atom. Specifically, the Co(1)-N(2) bond lengths were 2.0321 (15) Å for **Co1**, 2.033 (5) Å for **Co2**, 2.0362 (19) Å for **Co4**, and 2.021 (8) Å for **Co5**. In comparison, the Co(1)-N(1) bond lengths were 2.2918 (15) Å for **Co1**, 2.271 (15) Å for **Co2**, 2.3090 (19) Å for **Co4**, and 2.294 (8) Å for **Co5**, while the Co(1)-N(3) bond lengths were 2.2398 (15) Å for **Co1**, 2.228 (5) Å for **Co2**, 2.204 (2) Å for **Co4**, and 2.233 (9) Å for **Co5**. These consistently shorter Co(1)-N(2) bonds suggest a stronger coordination interaction between cobalt and the nitrogen atom at position N(2). This is consistent with similar findings in the literature [30,31], where shorter Co-N bonds to the central nitrogen are typically indicative of a stronger metal–ligand coordination. Similarly, the bond lengths of Co(1)-Cl(1) [2.2470 (5) Å for **Co1**, 2.2542 (18) Å for **Co2**, 2.2567 (7) Å for **Co4**, and 2.242 (3) Å for **Co5**] and Co(1)-Cl(2) [2.2689 (5) Å for **Co1**, 2.2538 (18) Å for **Co2**, 2.2979 (7) Å for **Co4**, and 2.259 (3) Å for **Co5**] exhibited slight variations while remaining generally consistent across the different cobalt atoms. This consistency in the Co-Cl bond lengths suggests a stable coordination environment, which is advantageous for predicting the behavior of these complexes in various chemical reactions. Additionally, the uniformity in these bond lengths can indicate the reliability of the synthesis method, ensuring the reproducibility and stability of the compounds. The nitrogen–carbon bond lengths N(1)-C(10) [1.432 (2) Å (**Co1**), 1.440 (7) Å (**Co2**), 1.435 (3) Å (**Co4**), and 1.453 (11) Å (**Co5**)] and N(3)-C(43) [1.435 (2) Å (**Co1**), 1.447 (8) Å (**Co2**), 1.445 (3) Å (**Co4**), and 1.410 (14) Å (**Co5**)] exhibit slight variations. These differences can be explained by the subtle variations in the local environment and electronic effects around each cobalt center. Factors such as coordination geometry, ligand field effects, and inter-ligand interactions can affect the bond lengths, resulting in the observed minor variations. Similar tendencies have been observed in other complexes of unsymmetrical bis(arylimino)pyridine [33,34,35]. The description of X-ray crystallographic studies can be found in the Supporting Information (SI). Finally, the bond lengths between F(1) and C(34) showed some variability [1.361 (2) Å for **Co1**, 1.354 (10) Å for **Co2**, 1.357 (3) Å for **Co4**, and 1.405 (16) Å for **Co5**], suggesting potential differences in the interactions between fluorine and carbon. The bond angles around **Co1**, **Co2**, **Co4**, and **Co5** in Table 2 show how *meta*-chloro, *ortho*- and *para*-di(4-fluorophenyl)methyl, and *ortho*-methyl substituents affect the coordination geometry of cobalt complexes. Comparing **Co1**, **Co2**, and **Co4** with **Co5**, significant differences are observed. For example, the N(1)-Co(1)-N(3) angle in **Co5** (101.8°) is notably smaller than in **Co1**, **Co2**, and **Co4** (approximately 150°), indicating greater steric hindrance around **Co5**. This steric effect is also evident in the N(2)-Co(1)-Cl(2) angle, which is larger in **Co5** (118.2°) compared to **Co1**, **Co2**, and **Co4**, suggesting more space around **Co5** for the chloride ligand. These findings support previous literature that has examined similar substituent effects on transition metal complexes [36]. These results highlight how the combination effect from the substituents on both of the N-aryl rings on the coordination environments around the metal center affect bond angles and overall geometries. The crystal data and structural refinements for **Co1**, **Co2**, **Co4,** and **Co5** can be found in (Table S1).

### 2.2. Ethylene Polymerization

Previous research has shown that aluminoxanes, such as MAO (methylaluminoxane) or MMAO (modified methylaluminoxane), are effective co-catalysts for activating the bis(imino)pyridine-cobalt catalyst. To evaluate the catalytic properties of **Co1** to **Co5**, both MAO and MMAO were used for comparison. We chose **Co4** as the initial catalyst to establish the optimal reaction conditions for polymerizations under these two co-catalysts. This involved systematically exploring the effects of co-catalyst amount, temperature, reaction duration, and ethylene pressure on the catalytic performance and molecular weight (*M_w_*) of the resulting polyethylene. Toluene was used as the solvent in all experimental procedures and the results of the polymerization experiments were summarized in Table 2, Table 3 and Table 4 with some of the general descriptions listed in the Supplementary Information.

#### 2.2.1. Optimization of Polymerization Conditions Using **Co4**/MAO

The optimization of reaction conditions for ethylene polymerization using **Co4**/MAO began by varying the co-catalyst amounts, represented by the Al/Co ratio (ranging from 2000:1 to 3250:1) (entries 1–5, Table 2). The highest catalytic activity of 4.0 × 10^6^ g mol^−1^ h^−1^ and a peak molecular weight of 20.5 kg mol^−1^ for polyethylene were observed at an Al/Co ratio of 3000:1 (entry 4, Table 2), with the operating temperature maintained at 30 °C, a reaction time of 30 min, and a reaction pressure of 10 atm ethylene. At a lower Al/Co ratio (2000:1), the catalytic activity was relatively lower (entries 1–3), while a further increase in the Al/Co ratio (3250:1) led to a slight reduction in both activity and molecular weight (entry 5). This trend suggests that the co-catalyst concentration has an optimal range, and beyond that, the excess co-catalyst may promote side reactions such as chain transfer and termination, leading to a reduction in the polymer’s molecular weight [37,38,39].

**Table 2:** Optimization of polymerization conditions using **Co4**/MAO ^a^.

Entry	T (°C)	t (min)	Al/Co	PE (g)	Activity ^b^	*M*_w_ ^c^	*M*_w_/*M* _n_^c^	*T*_m_ (°C) ^d^
1	30	30	2000	2.0	2.0	15.3	2.3	131.6
2	30	30	2500	2.4	2.4	15.7	1.9	130.7
3	30	30	2750	2.7	2.7	18.3	1.7	130.9
4	30	30	3000	4.0	4.0	20.8	2.2	131.6
5	30	30	3250	2.5	2.5	16.1	2.4	131.6
6	40	30	3000	4.1	4.1	20.5	2.6	131.8
7	50	30	3000	4.5	4.5	16.3	2.0	130.7
8	60	30	3000	4.9	4.9	14.6	2.2	130.4
9	70	30	3000	8.9	8.9	10.9	2.0	131.8
10	80	30	3000	7.2	7.2	7.5	2.0	129.1
11	90	30	3000	5.0	5.0	7.1	2.2	128.1
12	100	30	3000	1.6	1.6	6.4	2.9	128.3
13	70	5	3000	3.0	18.0	8.5	2.1	128.0
14	70	15	3000	6.9	13.7	10.0	2.4	129.1
15	70	45	3000	12.9	8.6	11.4	2.1	129.0
16	70	60	3000	13.3	6.6	12.7	2.7	128.8
17 ^e^	70	30	3000	4.3	4.3	8.2	2.5	129.0
18 ^f^	70	30	3000	0.9	0.9	2.2	2.3	128.0

^a^ General conditions: 2 μmol of **Co4**, 100 mL toluene, 10 atm C_2_H_4_; ^b^ 10^6^ g mol^−1^ h^−1^; ^c^ M_w_: kg mol^−1^, determined by GPC; ^d^ determined by DSC; ^e^ 5 atm of C_2_H_4_; ^f^ 1 atm of C_2_H_4._

**Table 3:** Optimization of polymerization conditions using **Co4**/MMAO ^a^.

Entry	T (°C)	t (min)	Al/Co	PE (g)	Activity ^b^	*M*_w_ ^c^	*M*_w_/*M*_n_ ^c^	*T*_m_ (°C) ^d^
1	30	30	2000	2.2	2.2	19.8	1.9	130.8
2	30	30	2500	2.4	2.4	21.6	2.0	130.7
3	30	30	3000	2.9	2.9	23.3	1.9	132.0
4	30	30	3500	4.1	4.1	21.0	2.0	131.5
5	30	30	4000	3.3	3.3	18.4	2.1	132.0
6	40	30	3500	4.5	4.5	20.0	2.1	131.6
7	50	30	3500	4.6	4.6	15.9	2.2	130.7
8	60	30	3500	4.8	4.8	15.0	2.2	130.5
9	70	30	3500	3.2	3.2	10.3	2.3	129.4
10	80	30	3500	0.9	0.9	9.0	2.6	128.9
11	90	30	3500	0.8	0.8	8.1	2.4	128.7
12	60	5	3500	2.1	13.0	11.8	1.1	130.0
13	60	15	3500	2.3	4.7	13.0	1.7	130.9
14	60	45	3500	5.3	3.5	19.3	2.3	131.9
15	60	60	3500	6.1	3.1	22.1	2.9	131.8
16 ^e^	60	60	3500	2.0	2.0	19.1	2.0	128.6
17 ^f^	60	60	3500	0.5	0.5	6.5	1.9	128.0

^a^ General conditions: 2 μmol of **Co4**, 100 mL toluene, 10 atm C_2_H_4_; ^b^ 10^6^ g mol^−1^ h^−1^; ^c^ M_w_: kg mol^−1^, determined by GPC; ^d^ determined by DSC; ^e^ 5 atm of C_2_H_4_; ^f^ 1 atm of C_2_H_4_.

**Table 4:** Evaluation of **Co1** to **Co5** with either MAO/MMAO under optimized conditions ^a^.

Entry	Precat.	Co-Cat.	Activity ^b^	*M_w_* ^c^	*M_w_/M_n_* ^c^	*T_m_* (°C) ^d^
1	**Co1**	MAO	9.2	8.3	2.6	129.5
2	**Co2**	MAO	9.1	14.5	2.4	130.7
3	**Co3**	MAO	7.1	25.7	2.3	131.8
4	**Co4**	MAO	8.9	10.9	2.0	131.8
5	**Co5**	MAO	8.0	17.9	2.1	131.3
6	**Co1**	MMAO	2.7	14.9	2.2	130.7
7	**Co2**	MMAO	2.6	29.6	2.1	131.9
8	**Co3**	MMAO	2.3	50.0	1.9	132.0
9	**Co4**	MMAO	4.8	15.0	2.2	131.7
10	**Co5**	MMAO	2.5	32.4	1.8	132.3

^a^ General conditions: 2 μmol of cobalt precatalyst, 100 mL toluene, 10 atm C_2_H_4_, 30 min, Al/Co ratio of 3000:1 and 70 °C for MAO, Al/Co ratio of 3500:1 and 60 °C for MMAO; ^b^ 10^6^ g mol^−1^ h^−1^; ^c^ determined by GPC, *M_w_*: kg mol^−1^; ^d^ determined by DSC.

**Figure 5:**
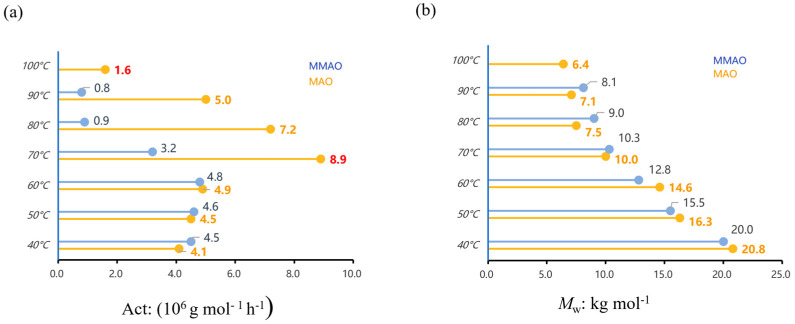
The activity in 10^6^ g mol^−1^ h^−1^ (**a**) and molecular weight in kg mol^−1^ (**b**) varying with reaction temperatures using **Co4** that is activated by either MAO or MMAO.

Next, the effect of reaction temperature on the catalytic activity and polymer properties was explored by varying the temperature from 30 to 100 °C (entries 6–12 in Table 2, Figure 5a). As the temperature increased from 30 to 70 °C, the catalytic activity gradually intensified, reaching a peak of 8.9 × 10^6^ g mol^−1^ h^−1^ at 70 °C (entry 9, Table 2). However, higher temperatures above 70 °C resulted in a decrease in catalytic activity, with values dropping to 7.2 × 10^6^ g mol^−1^ h^−1^ at 80 °C, 5.0 × 10^6^ g mol^−1^ h^−1^ at 90 °C, and a further decrease to 1.6 × 10^6^ g mol^−1^ h^−1^ at 100 °C. This decrease in catalytic activity at elevated temperatures can be attributed to the thermal instability of the active species, which is a well-documented phenomenon in similar catalytic systems [40]. Despite this, the system exhibited notable thermal stability, with only a 13% decrease in activity at 80 °C compared to the peak activity at 70 °C, and it still demonstrated an excellent catalytic performance even at 100 °C. This suggests that the **Co4**/MAO system retains a significant degree of stability at higher temperatures, with only a moderate decline in activity as the temperature increases. On the other hand, the molecular weight of the resulting polyethylene decreased with increasing temperature, from 20.8 kg mol^−1^ at 30 to 6.4 kg mol^−1^ at 100 °C (entries 4, 6–12 in Table 2 and Figure 5b). This reduction in molecular weight can be attributed to an enhanced rate of chain transfer reactions compared to chain propagation, which is common at higher operating temperatures. Additionally, the reduced solubility of the ethylene monomer at higher temperatures could contribute to the observed decrease in molecular weight, as chain termination could occur via β-hydrogen elimination to the ethylene monomer, resulting in polymers with vinyl end groups, as evidenced by the high-temperature ^1^H and ^13^C NMR spectra presented in the microstructural analysis section. Similar trends of decreasing molecular weight with increasing temperature due to the increased chain transfer rates at elevated temperatures have been reported in the literature [41].

The time/activity profile of **Co4**/MAO was assessed at intervals ranging from 5 to 60 min (entries 9, 13–16, Table 2), while maintaining the optimal Al/Co ratio of 3000:1 and reaction temperature of 70 °C. The results showed a gradual decline in catalytic activity over time, with the peak occurring at 5 min (18.0 × 10^6^ g mol^−1^ h^−1^) and the lowest level observed at 60 min (6.6 × 10^6^ g mol^−1^ h^−1^), reflecting the short induction period required to generate the active species. The polymerization system maintained a relatively extended lifespan during prolonged reactions, indicating that the **Co4**/MAO catalyst is stable over the course of the polymerization process.

Polymerization experiments at varying ethylene pressures (5 and 1 atm) were conducted to investigate the influence of pressure on the catalytic activity of **Co4**/MAO and polymer molecular weight (entries 9, 17, and 18, Table 2). The results clearly revealed that higher pressures enhanced both catalytic performance and polymer molecular weight. Specifically, at 5 atm of ethylene, the catalytic activity was nearly halved compared to 10 atm, with the molecular weight of polyethylene decreasing to 8.2 kg mol^−1^ (entry 17, Table 2). Conversely, at 1 atm, the catalytic activity dropped significantly to 0.9 × 10^6^ g mol^−1^ h^−1^, and the polymer molecular weight decreased to 2.2 kg mol^−1^. These findings suggest that the higher ethylene pressure improves the solubility of the ethylene monomer and facilitates better coordination and insertion, thereby enhancing both catalytic activity and molecular weight.

In the above optimization of polymerization conditions of **Co4**/MAO, the produced polyethylenes consistently exhibited a narrow dispersity (*M*_w_/*M*_n_ range from 1.7 to 2.9; entries 1–18, Table 2). The well-defined molecular weight distribution is indicative of a controlled chain growth process, with minimal variations in the chain lengths of the resulting polyethylene. This is a significant advantage for producing polymers with consistent and predictable properties, which is essential for many industrial applications. Furthermore, the melting temperatures of the produced polyethylenes ranged from 128.0 °C to 131.6 °C, which is optimal for high-density polyethylene (HDPE) with a narrow molecular weight distribution. The combination of narrow molecular weight distribution and high melting point indicates that **Co4**/MAO is a promising catalyst system for the synthesis of high-quality polyethylene materials.

#### 2.2.2. Optimization of Polymerization Conditions Using **Co4**/MMAO

In order to enhance the performance of **Co4**/MMAO, a similar optimization strategy was employed by substituting MMAO for MAO. The full set of results is detailed in Table 3. The impact of varying the Al/Co ratio on **Co4**/MMAO was studied by adjusting the ratio from 2000:1 to 4000:1 (entries 1–5, Table 3), with the temperature kept constant at 30 °C. The results showed a steady increase in catalytic activity, reaching a peak at a ratio of 3500:1 (entry 4, Table 3) with a value of 4.1 × 10^6^ g mol^−1^ h^−1^. Beyond this point, increasing the co-catalyst amounts led to a decline in activity, with the decreased value of 3.3 × 10^6^ g mol^−1^ h^−1^ at a ratio of 4000:1 (entry 5, Table 3). As for the molecular weight of the polyethylene, the highest value of 23.3 kg mol^−1^ was achieved at 3000:1 and decreased progressively as the ratio was increased at 4000:1. This trend is consistent with increased rates of chain transfer and termination reactions at higher co-catalyst concentrations [42].

**Figure 6:**
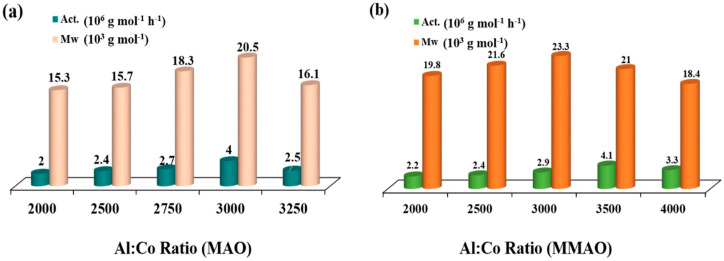
Catalytic activity and polymer *M*_w_ at different Al/Co ratios. (**a**) MAO, (**b**) MMAO.

Polymerizations conducted at temperatures ranging from 30 to 90 °C (entries 4, 6–11, Table 3) revealed a peak activity of 4.8 × 10^6^ g mol^−1^ h^−1^ at 60 °C (entry 4 in Table 3, Figure 6a). Although this peak activity was lower than that achieved with **Co4**/MAO, the optimal temperature of 60 °C further underscored the exceptional thermal stability of **Co4** catalyst. When temperatures exceeded 60 °C, the catalytic activity dropped to 3.2 × 10^6^ g mol^−1^ h^−1^ at 70 °C, 0.9 × 10^6^ g mol^−1^ h^−1^ at 80 °C, and further to 0.8 × 10^6^ g mol^−1^ h^−1^ at 90 °C (entries 9, 10 and 11, Table 3). Molecular weights of the polyethylene displayed a pattern akin to those observed with MAO (Figure 6b), decreasing from 21.0 to 8.1 kg mol^−1^ as the temperature increased from 30 to 90 °C. The polyethylenes also exhibited a relatively narrow dispersity throughout this temperature range (entries 4, 6–11, Table 3). The impact of run time on **Co4**/MMAO was examined at different intervals, ranging from 5 to 60 min (entries 8, 12–15, Table 3), while maintaining a constant temperature of 60 °C and an Al/Co ratio of 3500:1. The highest activity, measuring 13.0 × 10^6^ g mol^−1^ h^−1^, was observed at the 5-min mark (entry 12, Table 3). As the reaction time increased, the catalytic activity gradually decreased, reaching 3.1× 10^6^ g mol^−1^ h^−1^ after 60 min (entry 15, Table 3). Despite this decline, the activity levels remained significant, indicating the prolonged effectiveness of the active species. Furthermore, the molecular weight of the polyethylenes increased progressively over time.

The impact of ethylene pressure on **Co4**/MMAO was investigated under optimal conditions for run temperature and Al/Co ratio (entries 8, 16, and 17, Table 3). The highest catalytic activity was observed at the highest ethylene pressure, which is consistent with the findings from the MAO study. The catalytic activity decreased to 2.2 × 10^6^ g mol^−1^ h^−1^ at a pressure of 5 atm, and further reduced to 0.5 × 10^6^ g mol^−1^ h^−1^ at 1 atm. Correspondingly, the molecular weight of the polyethylene dropped to 19.1 kg mol^−1^ at 5 atm, and further declined to 6.5 kg mol^−1^ at 1 atm. These observations can be attributed to a common kinetic phenomenon, where the polymerization rate is directly proportional to the monomer concentration induced by pressure.

Similar to the case of **Co4**/MAO, the dispersity of the polyethylenes (*M*_w_/*M*_n_) is narrow, ranging from 1.1 to 2.9 across all polymerization conditions using **Co4**/MMAO, indicating that both co-catalysts are effective for producing high-quality polyethylene.

#### 2.2.3. Evaluation of **Co1** to **Co5** with Either Methyl Aluminoxane (MAO) or Modified-Methyl Aluminoxane (MMAO) Under Optimized Conditions

The catalytic performance of cobalt complexes **Co1, Co2, Co3,** and **Co5** were tested under the optimal conditions specified for either **Co4**/MAO (70 °C, Al/Co ratio of 3000:1, 30 min run time, 10 atm ethylene pressure) or **Co4**/MMAO (60 °C, Al/Co ratio of 3500:1, 30 min run time, 10 atm ethylene pressure) towards ethylene polymerization.

The experiment results are presented in Table 4. When MAO was used as a co-catalyst, all the cobalt precatalysts showed significant catalytic efficiency in ethylene polymerization. The efficiency ranged from 7.1 to 9.2 × 10^6^ g mol^−1^ h^−1^, with the **Co1** achieving the highest activity of 9.2 × 10^6^ g mol^−1^ h^−1^, closely followed by **Co2** and **Co4**. **Co5** showed a moderate activity of 8.0 × 10^6^ g mol^−1^ h^−1^, while **Co3**, with bulkier ortho-isopropyl substituents, displayed the lowest catalytic activity of 7.1 × 10^6^ g mol^−1^ h^−1^. In contrast, **Co3** produced polyethylene with the highest molecular weight of 25.7 kg mol^−1^, and then **Co5** with 17.9 kg mol^−1^, suggesting that steric hindrance from bulky *ortho*-isopropyl substituents may reduce chain transfer, thereby leading to longer polymer chains. This effect is indicative of how structural variations in the precatalysts can influence both catalytic efficiency and polymer properties, with larger substituents potentially shielding the active site and hindering chain transfer reactions, resulting in higher molecular weight polyethylenes.

**Figure 7:**
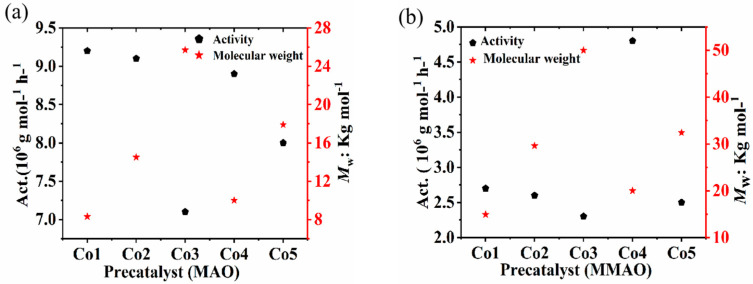
Comparison of polymerization activity and *M*_w_ for different precatalysts from **Co1** to **Co5** using MAO (**a**) and (**b**) MMAO (data in Table 4).

Similarly, the catalytic performance of **Co1**–**Co5** under MMAO (entries 6–10, Table 4) followed a similar trend but exhibited lower activities to those observed with MAO. Under MMAO, the **Co4** has the highest activity (4.8 × 10^6^ g mol^−1^ h^−1^) instead of **Co1** in the case of MAO. This can likely be explained by the different activation processes associated with each co-catalyst. The steric effects from the N-aryl substituents continued to significantly impact performance, with the least hindered 2,4,6-trimethyl **Co4** and 2,6-dimethyl **Co1** achieving higher catalytic ranges (entries 6 and 9, Table 4). Similarly, the bulkiest precatalysts, **Co3** (*M*_w_ = 50.0 kg mol^−1^) and **Co5** (*M*_w_ = 32.4 kg mol^−1^), have lower activities but produced the highest molecular weight polyethylene, further supporting the hypothesis that larger substituents shield the active site and suppress chain transfer, leading to higher molecular weight polyethylenes. Figure 7 illustrates the variation in the activities of catalysts and molecular weight of polyethylene produced using precatalysts **Co1**–**Co5** under (a) MAO and (b) MMAO. As seen, **Co1** and **Co4** consistently show higher activities and relatively moderate molecular weights, while **Co3** and **Co5**, despite their lower activities, produce polyethylenes with the highest molecular weights. These results highlight the critical role of catalyst structure in determining both catalytic efficiency and polymer characteristics, with steric effects playing a pivotal role in controlling polymerization outcomes. The comparison of activity and molecular weight at different temperatures (a) and reaction time (b) using the MAO/MMAO catalytic system can be found in Figures S5 and S6, while the GPC curves at different Al/Co ratios, temperatures, and reaction time using the MMAO catalytic system are provided in Figure S7. Most of the DSC curves of polyethylene samples are provided in Figure S8.

### 2.3. Microstructural Attributes of the Produced Polyethylenes

Table 2, Table 3 and Table 4 indicate that all polyethylene samples produced in this study have melting points (*T*_m_) ranging from 128.0 to 132.0 °C, which is typical for linear polyethylenes. The bis(imino)pyridine-cobalt catalysts employed in this research are well-known for their ability to produce polymers with minimal branching [43]. These catalysts demonstrate a high level of control over the polymer structure, enabling the synthesis of highly linear polyethylenes. To gain a deeper understanding of the microstructure and chain termination mechanism, high-temperature ^1^H/^13^C NMR spectroscopy were performed on selected polyethylene samples (entries 4 and 9, Table 4). For acquiring high-quality NMR spectra, the dissolution of these samples was carried out in deuterated tetrachloroethylene (C_2_D_2_Cl_4_) at a temperature of 100 °C ensuring sufficient solubility for effective spectral measurement.

**Figure 8:**
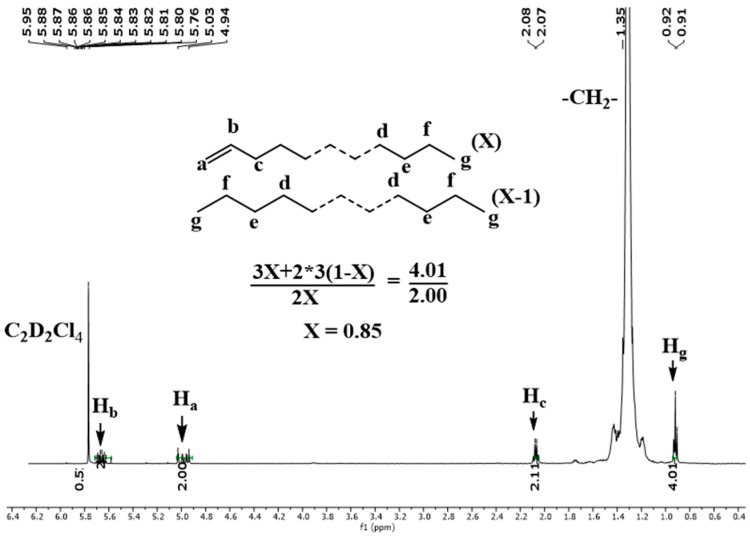
^1^H NMR spectrum of polyethylene produced using **Co4**/MAO (entry 4 in Table 4) recorded in C_2_D_2_Cl_4_ at 100 °C.

**Figure 9:**
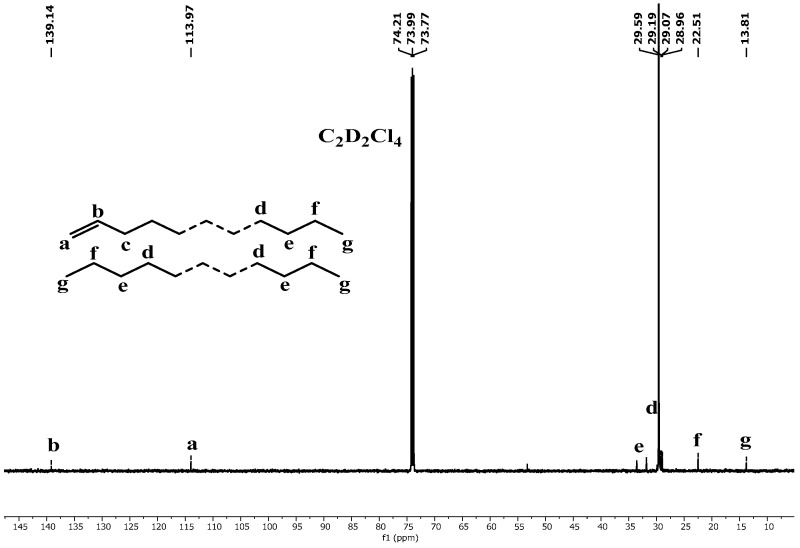
^13^C NMR spectrum of polyethylene produced using **Co4**/MAO (entry 4 in Table 4) recorded in C_2_D_2_Cl_4_ at 100 °C.

Using the **Co4**/MAO system (entry 4, Table 4) as a representative example, the ^1^H NMR spectra of the polyethylene sample in Figure 8 displayed a pronounced peak at 1.35 ppm, characteristic of the –CH_2_– repeating units, which further confirms the highly linear structure of the resulting polyethylene. Additionally, two downfield multiplet signals at 5.0 ppm and 5.8 ppm were attributed to the protons of the vinyl group (–CH=CH_2_). The peak at 5.0 ppm is attributed to two protons, while the peak at 5.8 ppm corresponds to a single proton. These vinyl protons are indicative of the termination mechanism, particularly β-hydride elimination [42], a well-known pathway for chain termination in catalyzed olefin polymerization. Meanwhile, the ^1^H NMR spectrum also revealed a prominent signal at δ 0.91 ppm, attributed to the saturated terminal methyl group (-CH_3_), with a relative integration value of 4.01. This integration suggests the coexistence of both saturated (-CH_3_) and unsaturated end groups (–CH=CH_2_) in the polyethylene by **Co4**/MAO, further supporting the notion that the chain termination process involves chain transfer reactions to alkyl aluminum species. This chain transfer reaction results in the formation of both saturated and unsaturated terminal groups, confirming the dual nature of chain termination mechanisms occurring during polymerization. The ^13^C NMR spectra in Figure 9 of the polyethylene prepared by **Co4**/MAO further supports these findings, confirming the highly linear nature of the polymer. The spectrum also clearly shows signals corresponding to both vinyl and saturated methyl end groups, further validating the presence of both types of terminal groups and the expected polymer microstructure. These results suggest that the **Co4**/MAO system not only promotes the formation of linear polyethylene but also favors β-hydride elimination for chain termination, resulting in vinyl-terminated polymer chains.

Similarly, the ^1^H/^13^C NMR spectra of polyethylene prepared by **Co4**/MMAO in Figures S9 and S10 revealed a comparable linear microstructure. Both saturated (-CH_3_) and unsaturated end groups (–CH=CH_2_) were identified in this sample as well, indicating that while the **Co4**/MMAO system also produces linear polyethylenes, the chain termination process occurs in a similar manner to that of **Co4**/MAO, involving a combination of chain transfer to alkyl aluminum species and β-hydride elimination.

To better understand the dominant microstructure and elucidate the chain termination mechanism in more detail, the relative proportion of vinyl-terminated polyethylene (–CH=CH_2_) and fully saturated polyethylene (-CH_3_) was evaluated. The molar fraction X representing the portion of vinyl-terminated polyethylene was calculated using the integration of the H_a_ and H_g_ signals in the ^1^H NMR spectrum (3X + 2 × 3 (1 − X)/2X = H_g_/H_a_). When X equals 1, it indicates 100% vinyl-terminated polyethylene. Once the value of X is determined, (1 − X) can be used to represent the proportion of fully saturated polyethylene (-CH_3_). From this analysis, a vinyl-terminated fraction of 0.85 (85%) was determined for the polyethylene prepared with **Co4**/MAO at 70 °C. This high vinyl-terminated fraction suggests that the chain termination process is predominantly governed by β-hydride elimination. In contrast, for the polyethylene produced using **Co4**/MMAO at 60 °C, the vinyl-terminated fraction was calculated to be 0.59 (59%). The lower fraction of vinyl-terminated end groups indicates a greater tendency for chain transfer to alkyl aluminum species in this system compared to **Co4**/MAO at 70 °C.

These results indicate that while both **Co4**/MAO and **Co4**/MMAO systems promote β-hydride elimination as the primary chain termination mechanism, the reaction conditions particularly the type of co-catalysts and temperature play significant roles in determining the proportion of vinyl-terminated polyethylenes. In summary, the polymerization systems studied here produce highly linear polyethylenes with both saturated and unsaturated end groups, with the dominant chain termination mechanism being β-hydride elimination. The ratio of vinyl-terminated to fully saturated polyethylene is influenced by reaction conditions, highlighting the importance of careful control of polymerization conditions in tailoring the structure and properties of the resulting polymers.

### 2.4. Comparative Analysis of the Present Catalyst System with Previously Documented Examples

The development of cobalt precatalysts for ethylene polymerization has been a central focus of research, highlighting several critical challenges associated with these catalysts. These challenges include limited catalytic activity, inadequate molecular weights of the resulting polymers, and broad molecular weight distributions. Moreover, persistent issues such as low thermal stability and the high demands for co-catalysts have impeded practical applications. Consequently, ongoing research initiatives are dedicated to improving catalytic performance and enabling the synthesis of novel polyethylene products to address these challenges [9].

As discussed in the introduction, steric hindrance introduced by *ortho*-substituted bulky groups, as seen in complex **B** (Scheme 1), plays a key role in protecting catalytic active sites, thereby enhancing both catalytic activity and thermal stability. A significant improvement in catalytic performance was achieved when one *ortho*-substituted bulky dibenzhydryl group was retained, while the other was shifted to the *para* position, resulting in complex **D**. This structural modification led to an improvement in both activity and stability. Further enhancement in thermal stability was observed when a fluoride group was incorporated at the *para* position of the bulky dibenzhydryl groups, leading to the development of complex **E**. Complex **E** exhibited a higher optimal polymerization temperature of 70 °C, significantly higher than the 40 °C optimal temperature of complexes **B** and **D**. Moreover, complex **E** demonstrated a maximum operating temperature of 80 °C, compared to the 50 °C limitation for complexes **B** and **D**.

Building on the structure of complex **E**, the newly developed complex **F** in this work was further optimized by adding an electron-withdrawing *meta*-chloro group. This modification enhanced the thermal stability of the catalyst, making complex **F** more resilient at higher temperatures. Complex **F** displayed exceptional thermal stability, maintaining an activity of 1.6 × 10^6^ g mol^−1^ h^−1^ at 100 °C—much higher than the maximum operating temperatures of complexes **B**, **D**, and **E**. When evaluating optimal temperature performance, complex F achieved the highest catalytic activity of 9.2 × 10^6^ g mol^−1^ h^−1^ at 70 °C. Although this value is slightly lower than that of complex **B** (9.87 × 10^6^ g mol^−1^ h^−1^) and complex **D** (18.1 × 10^6^ g mol^−1^ h^−1^), both of which exhibit a higher activity at lower temperatures (40 °C), complex F stands out because it can maintain a high activity at elevated temperatures. In contrast, complexes **B** and **D**, optimized for lower temperatures, would likely degrade if exposed to higher thermal conditions. Complex **E** also reaches its peak activity at 70 °C, showing a value of 10.2 × 10^6^ g mol^−1^ h^−1^ when using **Co4**/MAO after a shorter reaction time of 15 min. In comparison, complex **F** achieved a higher activity of 13.7 × 10^6^ g mol^−1^ h^−1^, demonstrating superior performance.

**Figure 10:**
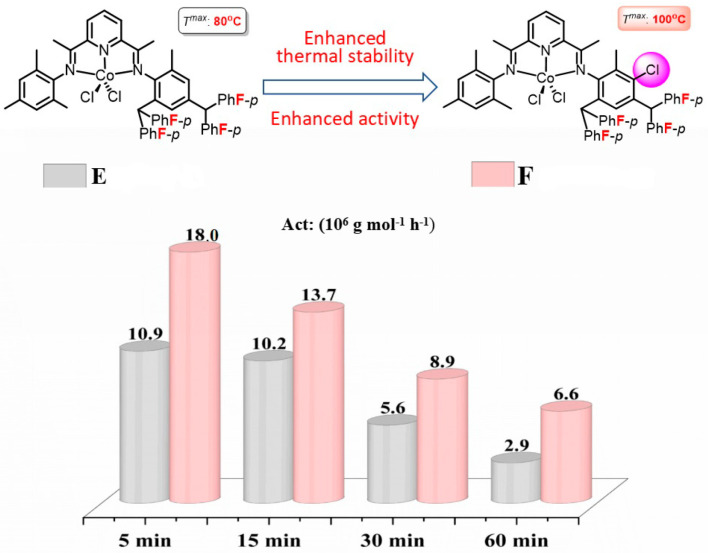
Comparative analysis of activity levels of compounds **E** (Scheme 1) and **F** (this work) over time at 70 °C. All experiments were conducted under optimized conditions at 10 atm of C_2_H_4_ using **MAO** as the co-catalyst.

To further assess the performance of complex **F**, we compared its catalytic behavior with complex **E** under identical reaction conditions at varying time intervals. As shown in Figure 10, complex **F** consistently outperformed complex **E** at all measured time points—5 min, 15 min, 30 min, and 60 min—when tested at the same optimal temperature of 70 °C. At 5 min, complex **E** showed an activity of 10.9 × 10^6^ g mol^−1^ h^−1^, while complex F exhibited a significantly higher activity of 18.0 × 10^6^ g mol^−1^ h^−1^. This early advantage indicates that complex **F** is more reactive or efficient than complex **E** in the initial stages of the reaction. As the reaction progressed, the activity of complex E decreased to 10.2 × 10^6^ g mol^−1^ h^−1^ at 15 min, while complex **F** maintained a higher activity of 13.7 × 10^6^ g mol^−1^ h^−1^. This trend continued with the activity of complex **E** dropping to 5.6 × 10^6^ g mol^−1^ h^−1^ at 30 min, while the activity of complex **F** remained higher at 8.9 × 10^6^ g mol^−1^ h^−1^. By 60 min, the activity of complex **E** had further decreased to 2.9 × 10^6^ g mol^−1^ h^−1^, while the activity of complex F remained at 6.6 × 10^6^ g mol^−1^ h^−1^. Over time, complex **F** consistently exhibited nearly double the activity of complex **E**, underscoring its greater stability and efficiency over extended periods.

Compared to complexes **B**, **D**, and **E**, which show higher activity at lower temperatures but fail to sustain performance under elevated thermal conditions, complex **F** stands out as the most efficient and effective compound. Its ability to maintain a high activity at 70 °C and demonstrate exceptional thermal stability at 100 °C makes it the most promising catalyst for high-performance applications that demand both efficiency and thermal stability.

In conclusion, complex F in this work outperforms previous catalysts in both catalytic activity and thermal stability, particularly at higher temperatures. With its superior performance at 70 °C and exceptional thermal stability at 100 °C, complex F is the most promising catalyst for advanced ethylene polymerization. Its combination of high activity, resilience, and thermal stability positions it as the ideal candidate for high-performance applications in industrial settings.

## 3. Experimental Section

### 3.1. Synthesis of 4,6-Bis(bis(4-fluorophenyl)methyl)-3-chloro-2-methylaniline (***X3***)

A 500-mL round-bottom flask containing a magnetic stir bar was charged with 10.6 g (75 mmol) of 3-chloro-2-methylaniline (**X1**), and 16.5 g (75 mmol) of bis(4-fluorophenyl)-methanol (**X2**). The mixture was stirred at 160 °C for 30 min to form a homogeneous solution. A catalytic amount of ZnCl_2_ (2.0 g) in 5 mL of HCl was then added dropwise to the solution, and the reaction was allowed to continue stirring at 160 °C for 5 h. After cooling, the solid product was dissolved in 250 mL of dichloromethane and washed sequentially with saturated aqueous NH_4_Cl, followed by NaCl. The organic phase was separated, dried over anhydrous MgSO_4_, and concentrated using a rotary evaporator. Since precipitation was not observed with various solvents, alumina column chromatography was employed using petroleum ether/ethyl acetate (*v*/*v* = 500/1) as the eluent, yielding the product as a yellow powder **X3** (14.5 g, 53%). ^1^H NMR (400 Hz, CDCl_3_, TMS) showed peaks at δ 6.95–6.82 (m, 17H, Ar), 5.83 (s, 1H, –CH–), 5.77 (s, 1H, –CH–), 3.58 (bs, 2H, –NH_2_), 2.26 (s, 3H, –CH_3_). The ^13^C NMR spectrum (101 MHz, CDCl_3_, TMS) showed peaks at δ 162.99, 162.61, 160.55, 160.18, 141.97, 138.96, 138.93, 137.50, 137.46, 133.41, 130.66, 130.58, 130.28, 126.08, 120.78, 115.68, 115.46, 115.16, 114.95, 77.48, 77.16, 76.84, 52.30, 50.75, 14.70, 1.16.

### 3.2. Synthesis of 2-Acetyl-6-[1-(3-chloro-4,6-bis((di(4-fluorophenyl)methyl)-2-methylphenylimino)-ethyl]pyridine (***X4***)

A total of 2.4 g (15 mmol) of 2,6-diacetylpyridine and 0.057 g (0.3 mmol) of *p*-toluene sulfonic acid were added to a 250-mL two-neck round-bottom flask containing 100 mL of toluene. The mixture was stirred and heated to reflux at 115 °C. After 45 min, a solution of 9.4 g (15 mmol) of 4,6-bis(bis(4-fluorophenyl) methyl)-3-chloro-2-methylaniline **X3** in toluene was added dropwise to the reaction mixture. The mixture was refluxed for an additional 10 h, with the completion of the reaction confirmed by TLC analysis. Then, the mixture was cooled to room temperature. All volatile components were removed under reduced pressure using a vacuum rotary. The residual solid was purified using basic alumina column chromatography, with petroleum ether/ethyl acetate (*v*/*v* = 500/1) as the eluent, resulting in the formation of the title compound **X4** as a yellow solid yielding 8.0 g (72%). The ^1^H NMR spectrum (400 MHz, CDCl_3_, TMS) showed peaks at δ 8.38 (d, *J* = 7.9 Hz, 1H), 8.13 (d, *J* = 7.6 Hz, 1H), 7.93 (t, *J* = 7.8 Hz, 1H), 6.99–6.66 (m, 17H), 6.16 (s, 1H), 5.85 (s, 1H), 5.26 (s, 1H), 3.72 (q, *J* = 7.0 Hz, 3H), 2.72 (s, 3H), 2.05 (s, 3H), 1.67 (s, 3H). The ^13^C NMR spectrum (101 MHz, CDCl_3_, TMS) showed peaks at δ 199.98, 169.34, 162.75, 160.38, 154.83, 152.70, 147.25, 138.52, 138.31, 137.76, 137.54, 136.20, 133.67, 131.38, 130.90, 130.83, 130.76, 130.67, 130.60, 130.54, 130.47, 129.93, 124.56, 124.40, 123.08, 115.48, 115.40, 115.33, 115.27, 115.19, 115.14, 115.11, 114.93, 77.48, 77.16, 76.84, 52.39, 50.85, 25.74, 17.02, 15.66. FT-IR (cm^−1^): 3060 (w), 3012 (w), 2926 (w), 2836 (w), 2829 (w), 1804 (w), 1649 (v (C=N), s), 1584 (w), 1528 (w), 1491 (m), 1463 (w), 1429 (w), 1364 (s), 1309 (m), 1234 (m), 1150 (w), 1120 (w), 1078 (w), 1042 (w), 1014 (w), 990 (w), 940 (w), 892 (w), 828 (w), 816 (m), 790 (w), 760 (s), 734 (m), 701 (m), 668 (w).

### 3.3. Synthesis of 2-[1-(3-Chloro-4,6-bis((di(4-fluorophenyl)methyl)-2-methylphenylimino)-ethyl]-6-(arylimino)ethyl] (***L1*** to ***L5***)

**L1** (Ar = 2,6-Me_2_C_6_H_3_)

A mixture of imino-ketone (1.378 g, 2.00 mmol) and 2,6-dimethyl aniline (0.290 g, 2.40 mmol), along with *p*-toluenesulfonic acid (0.152 g) were mixed with 20 mL of tetraethyl silicate in a 250-mL flask. The mixture was refluxed and heated at 150 °C under nitrogen atmosphere for 5 h. Upon completion of the reaction (checked by TLC), the reaction mixture was allowed to cool to ambient temperature and tetraethyl silicate was evaporated under reduced pressure, and the resulting solid was eluted with petroleum ether/ethyl acetate (4:1, *v*/*v*) on an alumina column. The second fraction obtained during elution was concentrated to produce a yellow powder (0.53 g, 31% yield). ^1^H NMR (400 MHz, CDCl_3_) δ 8.49 (d, *J* = 7.8 Hz, 1H), 8.28 (d, *J* = 7.8 Hz, 1H), 7.91 (t, *J* = 7.9 Hz, 1H), 6.99–6.71 (m, 19H), 6.17 (s, 1H), 5.86 (s, 1H), 2.18 (s, 3H), 2.11–2.03 (m, 9H), 1.69 (s, 3H). ^13^C NMR (101 MHz, CDCl_3_) δ 169.63, 167.19, 162.82, 162.79, 162.75, 162.71, 160.39, 160.36, 160.31, 160.27, 155.41, 154.60, 148.79, 147.71, 138.70, 138.67, 138.61, 138.57, 138.46, 138.43, 137.89, 137.86, 137.00, 135.86, 133.60, 131.30, 130.94, 130.85, 130.76, 130.67, 130.60, 130.57, 130.49, 129.88, 128.06, 125.51, 125.48, 124.37, 123.24, 122.64, 122.21, 115.42, 115.36, 115.30, 115.21, 115.15, 115.11, 115.09, 114.90, 77.48, 77.16, 76.84, 52.38, 50.82, 18.10, 18.06, 17.12, 16.53, 15.66. ^19^F NMR (565 MHz, CDCl_3_) δ −116.22, −116.35, −116.51, −116.65. FT-IR (cm^−1^): 3045 (w), 2965 (w), 2921 (w), 1645 (v (C=N), s), 1595 (m), 1566 (w), 1504 (s), 1460 (m), 1427 (w), 1359 (m), 1321 (w), 1298 (m), 1224 (s), 1153 (m), 1121 (w), 1094 (w), 1035 (w), 1015 (m), 968 (w), 870 (w), 820 (s), 764 (m), 744 (w), 711 (w), 691 (w). The calculated elemental analysis for C_50_H_40_ClF_4_N_3_ (794.33) with [EtOH] is as follows: C, 74.32; H, 5.52; N, 5.00. The found values were C, 73.98; H, 5.00; N, 4.88.

**L2** (Ar = 2,6-Et_2_C_6_H_3_)

Using a method akin to the one outlined for the production of **L1**, **L2** was prepared as a yellow powder (0.57 g, 32% yield). The ^1^H NMR spectrum (400 MHz, CDCl_3_, TMS) shows the following chemical shifts: δ 8.47 (d, *J* = 7.8 Hz, 1H), 8.27 (d, *J* = 7.8 Hz, 1H), 7.91 (t, *J* = 7.8 Hz, 1H), 7.16–6.70 (m, 19H), 6.17 (s, 1H), 5.86 (s, 1H), 2.39 (ddt, *J* = 18.5, 14.9, 7.5 Hz, 3H), 2.18 (s, 3H), 2.07 (s, 3H), 1.56 (s, 4H), 1.26–1.08 (m, 6H). The ^13^C NMR spectrum (100 MHz, CDCl_3_, TMS) shows the following chemical shifts: δ 169.66, 166.98, 162.83, 162.80, 162.75, 162.71, 160.39, 160.36, 160.31, 160.27, 155.39, 154.61, 147.79, 147.73, 138.71, 138.67, 138.61, 138.58, 138.46, 138.43, 137.90, 137.87, 137.03, 135.87, 133.60, 131.30, 131.27, 130.95, 130.87, 130.85, 130.77, 130.68, 130.60, 130.57, 130.50, 129.89, 126.12, 124.37, 123.57, 122.63, 122.20, 115.42, 115.37, 115.30, 115.21, 115.15, 115.12, 115.09, 114.90, 77.48, 77.16, 76.84, 52.39, 50.82, 24.75, 24.72, 17.12, 16.89, 15.67, 13.88, 13.86, 1.17. ^19^F NMR (565 MHz, CDCl_3_) δ −116.20, −116.32, −116.48, −116.62. The FT-IR spectrum (cm^−1^) shows the following peaks: 3030 (w), 2962 (w), 2865 (w), 1642 (v (C=N), s), 1598 (m), 1574 (w), 1504 (s), 1457 (m), 1415 (w), 1365 (m), 1327 (w), 1300 (w), 1265 (w), 1224 (s), 1156 (m), 1124 (w), 1094 (w), 1074 (w), 1018 (w), 968 (w), 932 (w), 882 (w), 853 (w), 841 (w), 817 (s), 767 (m), 744 (w), 714 (w). The calculated elemental analysis for C_52_H_44_ClF_4_N_3_ (822.39) with [2EtOH] is as follows: C, 72.76; H, 5.64; N, 4.90. The found values were C, 73.07; H, 5.49; N, 4.53.

**L3** (Ar = 2,6-*i*Pr_2_C_6_H_3_)

Using a method akin to the one outlined for the production of **L1**, **L3** was prepared and obtained as a brown powder with a yield of 0.49 g (28%). The ^1^H NMR spectrum (400 MHz, CDCl_3_, TMS) showed peaks at δ 8.45 (dd, *J* = 7.7, 4.9 Hz, 1H), 8.26 (d, *J* = 7.7 Hz, 1H), 7.90 (t, *J* = 7.8 Hz, 1H), 7.22–6.71 (m, 19H), 6.17 (s, 1H), 5.86 (s, 1H), 2.87–2.69 (m, 2H), 2.33–2.16 (m, 3H), 2.07 (s, 3H), 1.70 (s, 3H), 1.29–1.11 (m, 12H). The ^13^C NMR spectrum (100 MHz, CDCl_3_, TMS) exhibited peaks at δ 169.68, 162.85, 162.73, 160.33, 160.29, 154.66, 147.69, 138.68, 138.61, 138.43, 137.92, 137.07, 136.04, 135.92, 133.62, 131.30, 130.96, 130.88, 130.85, 130.77, 130.68, 130.61, 130.58, 130.50, 129.91, 124.39, 123.97, 123.22, 122.80, 122.29, 115.42, 115.38, 115.32, 115.21, 115.17, 115.13, 115.11, 114.92, 77.48, 77.16, 76.84, 52.39, 50.82, 28.53, 28.46, 23.41, 23.05, 23.03, 17.28, 17.14, 15.69. ^19^F NMR (565 MHz, CDCl_3_) δ −116.28, −116.42, −116.58, −116.71. The FT-IR spectrum (cm^−1^) displayed peaks at 3050 (w), 2959 (w), 2865 (w), 1645 (v (C=N), s), 1598 (m), 1566 (w), 1507 (s), 1457 (m), 1439 (w), 1349 (m), 1324 (w), 1298 (w), 1227 (s), 1189 (m), 1156 (m), 1124 (w), 1094 (w), 1077 (w), 1047 (w), 1003 (m), 985 (w), 932 (w), 867 (w), 817 (w), 770 (w), 738 (w), and 688 (w). The calculated elemental analysis for C_54_H_48_ClF_4_N_3_ (850.44) with [MeOH] is as follows: C, 74.86; H, 5.94; N, 4.76. The found values were C, 75.19; H, 5.70; N, 4.58.

**L4** (Ar = 2,4,6-Me_3_C_6_H_2_)

Using a method akin to the one outlined for the production of **L1**, **L4** was obtained as a yellow powder with a yield of 0.56 g (32%). The ^1^H NMR spectrum (400 MHz, CDCl_3_, TMS) showed signals at δ 8.50 (d, *J* = 7.8 Hz, 1H), 8.27 (d, *J* = 7.8 Hz, 1H), 7.90 (t, *J* = 7.8 Hz, 1H), 6.98–6.70 (m, 19H), 6.17 (s, 1H), 5.86 (s, 1H), 2.30 (s, 3H), 2.18 (s, 3H), 2.07–1.95 (m, 9H), 1.69 (s, 4H). The ^13^C NMR spectrum (100 MHz, CDCl_3_, TMS) displayed peaks at δ 169.65, 167.51, 162.80, 162.75, 162.71, 160.39, 160.36, 160.31, 160.27, 155.48, 154.57, 147.72, 146.16, 138.71, 138.68, 138.61, 138.58, 138.47, 138.44, 137.89, 137.86, 136.98, 135.86, 133.60, 132.50, 131.31, 130.94, 130.85, 130.77, 130.67, 130.60, 130.57, 130.49, 129.88, 128.99, 128.75, 125.42, 125.39, 124.38, 122.69, 122.18, 115.42, 115.37, 115.30, 115.21, 115.16, 115.11, 115.09, 114.90, 77.48, 77.16, 76.84, 52.38, 50.82, 29.85, 27.06, 20.88, 18.03, 18.00, 17.73, 17.12, 16.98, 16.50, 15.66, 1.17. ^19^F NMR (565 MHz, CDCl_3_) δ −116.25, −116.38, −116.54, −116.69. The FT-IR spectrum (cm^−1^) displayed peaks at 3078 (w), 3062 (w), 2962 (w), 2918 (w), 2847 (w), 1648 (v (C=N), s), 1589 (w), 1577 (w), 1507 (s), 1454 (w), 1427 (w), 1368 (m), 1309 (w), 1256 (w), 1218 (s), 1177 (w), 1156 (m), 1112 (w), 1094 (m), 1012 (m), 968 (w), 882 (w), 820 (s), 797 (w), 758 (w), 747 (w), 708 (w), and 673 (w). The calculated elemental analysis for C_51_H_42_ClF_4_N_3_ (808.36) with [H_2_O] is as follows: C, 74.13; H, 5.37; N, 5.08. The found values were C, 74.52; H, 5.42; N, 4.65.

**L5** (Ar = 2,6-Et_2_-4-MeC_6_H_2_)

Using a method akin to the one outlined for the production of **L1**, **L5** was synthesized as a yellow powder with a yield of 0.57 g (32%). The ^1^H NMR spectrum (400 MHz, CDCl_3_, TMS) showed peaks at δ 8.47 (d, *J* = 7.8 Hz, 1H), 8.27 (d, *J* = 7.8 Hz, 1H), 7.91 (t, *J* = 7.8 Hz, 1H), 7.16–6.70 (m, 19H), 6.17 (s, 1H), 5.86 (s, 1H), 2.39 (ddt, *J* = 18.5, 14.9, 7.5 Hz, 3H), 2.18 (s, 3H), 2.07 (s, 3H), 1.56 (s, 4H), 1.26–1.08 (m, 6H). The ^13^C NMR spectrum (100 MHz, CDCl_3_, TMS) showed peaks at δ 169.67, 162.85, 162.82, 162.77, 162.72, 160.38, 160.32, 155.42, 154.60, 147.73, 138.69, 138.62, 138.58, 138.46, 137.91, 137.88, 137.01, 135.87, 133.61, 132.81, 131.30, 130.95, 130.87, 130.85, 130.77, 130.68, 130.60, 130.58, 130.50, 129.89, 126.88, 124.38, 122.72, 122.18, 115.42, 115.37, 115.31, 115.21, 115.16, 115.12, 115.10, 114.91, 77.48, 77.16, 76.84, 52.39, 50.82, 24.74, 24.72, 21.15, 17.13, 16.87, 15.68, 14.00, 1.17. ^19^F NMR (565 MHz, CDCl_3_) δ −116.27, −116.40, −116.56, −116.70. The FT-IR spectrum (cm^−1^) showed peaks at 3068 (w), 2965 (w), 2921 (w), 1645 (v (C=N), s), 1572 (w), 1501 (s), 1460 (m), 1415 (w), 1398 (w), 1365 (m), 1315 (w), 1256 (w), 1218 (s), 1177 (w), 1156 (m), 1118 (w), 1080 (w), 1012 (w), 994 (w), 882 (w), 823 (s), 791 (w), 764 (w), 723 (w), and 670 (w). The calculated elemental analysis for C_53_H_46_ClF_4_N_3_ (836.42) with [2MeOH] is as follows: C, 73.36; H, 6.04; N, 4.67. The found values were C, 73.40; H, 5.66; N, 4.44.

### 3.4. Synthesis of 2-[1-(3-Chloro-4,6-bis((di(4-fluorophenyl)methyl)-2-methylphenylimino)-ethyl]-6-[1-(arylimino)ethyl]pyridylcobalt(II) Chlorides (***Co1*** to ***Co5***)

**Co1** (Ar = 2,6-Me_2_C_6_H_3_)

**L1** (0.127 g, 0.16 mmol) and CoCl_2_ 6H_2_O (0.038 g, 0.16 mmol) were combined in a mixture of dichloromethane (5 mL) and ethanol (4 mL) in a Schlenk tube. The resulting reaction mixture was stirred at room temperature for 12 h, and the volatile components were evaporated under reduced pressure. The resulting product was precipitated with diethyl ether (30 mL). The precipitate was filtered, washed with diethyl ether, and dried under reduced pressure. **Co1** was obtained as a brown powder with a yield of 0.14 g (84%). The FT-IR spectrum (cm^−1^) of **Co1** showed the following peaks: 3045 (w), 2947 (w), 2918 (w), 1628 (v, C=N), 1589 (s), 1507 (s), 1465 (m), 1436 (w), 1368 (m), 1315 (w), 1253 (w), 1230 (s), 1156 (s), 1094 (m), 1012 (m), 979 (w), 935 (w), 882 (w), 826 (s), 788 (w), 767 (w), 720 (w), and 688 (w). The calculated elemental analysis for C_51_H_43_Cl_3_CoF_4_N_3_ (939.20) with [3H_2_O] is as follows: C, 61.67; H, 4.97; N, 4.23. The found values were C, 61.55; H, 4.49; N, 4.24.

**Co2** (Ar = 2,6-Et_2_C_6_H_3_)

Using the same method and molar ratios as described for **Co1**, **Co2** was obtained as a brown powder (0.15 g, 89% yield). The FT-IR spectrum (cm^−1^) showed peaks at 3068 (w), 2962 (w), 2874 (w), 1630 (v (C=N), w), 1504 (s), 1451 (m), 1368 (m), 1318 (w), 1259 (w), 1224 (s), 1156 (s), 1094 (m), 1015 (m), 976 (w), 935 (w), 876 (w), 829 (s), 811 (s), 773 (w), 744 (w), and 711 (w). The calculated elemental analysis for C_53_H_47_Cl_3_CoF_4_N_3_ (967.26 g) with [CH_2_Cl_2_] is as follows: C, 61.64; H, 4.69; N, 3.99. The found values were C, 62.09; H, 4.59; N, 4.12.

**Co3** (Ar = 2,6-*i*Pr_2_C_6_H_3_)

Using the same method and molar ratios as described for **Co1**, **Co3** was obtained as a brown powder with a yield of 0.15 g (86%). The FT-IR spectrum (cm^−1^) showed peaks at 3053 (w), 2956 (w), 2918 (w), 1628 (v (C=N) (s), 1580 (s), 1507 (s), 1463 (m), 1439 (w), 1374 (m), 1318 (w), 1268 (w), 1227 (s), 1153 (m), 1097 (w), 1009 (w), 938 (w), 909 (w), 832 (s), 791 (w), 773 (w), 741 (w), and 717 (w). The calculated elemental analysis for C_55_H_51_Cl_3_CoF_4_N_3_ (995.31) with [MeOH] is as follows: C, 65.47; H, 5.40; N, 4.09. The found values were C, 65.22; H, 4.90; N, 4.05.

**Co4** (Ar = 2,4,6-Me_3_C_6_H_2_)

Using the same method and molar ratios as described for **Co1**, **Co4** was obtained as a green powder (0.15 g, 89% yield). The FT-IR spectrum (cm^−1^) showed peaks at 3030 (w), 2909 (w), 2862 (w), 1625 (C=N) w, 1592 (m), 1504 (s), 1465 (m), 1427 (w), 1368 (m), 1315 (w), 1253 (w), 1218 (s), 1156 (m), 1103 (w), 1015 (m), 973 (w), 873 (w), 832 (s), 811 (s), 791 (w), 767 (w), and 741 (w). The calculated elemental analysis for C_52_H_45_Cl_3_CoF_4_N_3_ (953.23) with [MeOH] is as follows: C, 64.61; H, 5.01; N, 4.26. The found values were C, 64.60; H, 4.49; N, 4.22.

**Co5** (Ar = 2,6-Et_2_-4-MeC_6_H_2_)

Using the same method and molar ratios as described for **Co1**, **Co5** was obtained as a brown powder (0.15 g, 87% yield). The FT-IR spectrum of **Co5** (cm^−1^) showed peaks at 3053 (w), 2962 (w), 2874 (w), 1622 (v (C=N) (s), 1598 (s), 1507 (s), 1463 (m), 1436 (w), 1368 (m), 1318 (w), 1265 (w), 1221 (s), 1156 (m), 1133 (w), 1095 (m), 1009 (m), 935 (w) 876 (w), 823 (s), 782 (w), and 735 (w). The calculated elemental analysis for (C_54_H_49_Cl_3_CoF_4_N_3_, (981.28) with [2H_2_O] is as follows: C, 63.76; H, 5.25; N, 4.13. The found values were C, 63.18; H, 4.79; N, 3.97.

## 4. Conclusions

This study presents the synthesis and evaluation of a series of unsymmetrical 2,6-bis(arylimino)pyridine cobalt(II) complexes (**Co1** to **Co5**), which were designed with sterically and electronically modified N-aryl substituents to enhance their catalytic performance in ethylene polymerization. Upon activation with aluminoxanes (MAOs) or modified-methyl aluminoxanes (MMAOs), the catalysts exhibited impressive catalytic activity, with **Co1** achieving the highest activity of 9.2 × 10^6^ g mol^−1^ h^−1^ at 70 °C. Notably, the catalysts demonstrated exceptional thermal stability, with complex Co4 maintaining high activity even at elevated temperatures (1.6 × 10^6^ g mol^−1^ h^−1^ at 100 °C), making them suitable for industrial applications where robust performance under high-temperature conditions is essential. The key to the catalysts’ success lies in the strategic incorporation of bulky para-fluorodibenzyl groups and electron-withdrawing meta-chloro substituents. The steric hindrance provided by the *para*-fluorodibenzyl groups enhanced the stability of the catalysts, while the *meta*-chloro group increased the Lewis acidity of the cobalt center, thereby improving catalytic efficiency and polymerization activity. These design features allowed for the production of high linearity polyethylene with narrow molecular weight distributions and molecular weights ranging from 6.4 to 50.0 kg mol^−1^, further confirming the catalysts’ effectiveness in controlling polymerization. The study underscores the importance of fine-tuning the steric and electronic properties of the N-aryl substituents to optimize catalyst performance. Certain configurations of these substituents led to the higher activity and stability during the polymerization process. These findings not only advance the understanding of cobalt-based catalysts, but also provide valuable insights for the development of next-generation catalysts for polyethylene production.

In conclusion, the strategic modification of cobalt(II) chloride N^N^N-pincer complexes through tailored steric and electronic adjustments has resulted in catalysts with high activity, excellent thermal stability, and precise control over the polymer molecular weight. These results contribute to the ongoing efforts to develop more efficient, thermally robust cobalt-based catalysts for industrial-scale polyethylene production. Further research is needed to explore additional structural modifications and to extend these findings to other polymerization processes, paving the way for the design of more advanced catalysts for a range of industrial applications.

## Figures and Tables

**Table 1:** Selected bond lengths (Å) and bond angles (°) for **Co1**, **Co2**, **Co4,** and **Co5**.

Selected Bond Lengths (Å)				
	Co1	Co2	Co4	Co5
Co(1)-N(1)	2.2918 (15)	2.271 (15)	2.3090 (19)	2.294 (8)
Co(1)-N(2)	2.0321 (15)	2.033 (5)	2.0362 (19)	2.021 (8)
Co(1)-N(3)	2.2398 (15)	2.228 (5)	2.204 (2)	2.233 (9)
Co(1)-Cl(1)	2.2470 (5)	2.2542 (18)	2.2567 (7)	2.242 (3)
Co(1)-Cl(2))	2.2689 (5)	2.2538 (18)	2.2979 (7)	2.259 (3)
N(1)-C(10)	1.432 (2)	1.440 (7)	1.435 (3)	1.453 (11)
N(3)-C(43)	1.435 (2)	1.447 (8)	1.445 (3)	1.410 (14)
F(1)-C(34)	1.361 (2)	1.354 (10)	1.357 (3)	1.405 (16)
**Selected Bond Angles (°)**				
N(1)-Co(1)-N(2)	74.57 (6)	74.40 (18)	73.65 (7)	75.5 (3)
N(1)-Co(1)-N(3)	149.96 (6)	150.44 (18)	150.53 (7)	101.8 (2)
N(2)-Co(1)-N(3)	75.46 (6)	76.05 (19)	76.90 (8)	75.1 (3)
N(1)-Co(1)-Cl(1)	97.67 (4)	92.86 (14)	96.86 (5)	92.2 (2)
N(2)-Co(1)-Cl(1)	122.45 (4)	123.07 (16)	122.57 (6)	126.4 (3)
N(3)-Co(1)-Cl(1)	99.91 (4)	102.52 (16)	97.92 (5)	101.8 (2)
N(2)-Co(1)-Cl(2)	125.16 (4)	123.49 (16)	126.23 (5)	118.2 (3)
N(3)-Co(1)-Cl(2)	96.50 (4)	96.96 (2)	105.59 (6)	96.2 (2)
Cl(1)-Co(1)-Cl(2)	112.39 (19)	113.26 (7)	110.44 (3)	115.32 (12)
N(1)-Co(1)-Cl(2)	99.05 (4)	99.93 (14)	92.82 (5)	101.3 (2)

## Data Availability

The authors have made available all the samples of the organic compounds, cobalt complexes, and data supporting the study.

## Supplementary Materials

The following supporting information can be downloaded at: https://www.mdpi.com/article/10.3390/molecules30040859/s1, Figure S1: ^1^H NMR and ^13^C NMR spectra of Sterically Hindered Aniline **X3** and **X4**; Figure S2: ^1^H NMR and ^13^C NMR spectra of imino-ketone and ligands **L1**–**L5**; Figure S3: ^19^F NMR spectra of ligands **L1**–**L5**; Figure S4: FT-IR spectra of ligands **L1**–**L5** and Complexes **Co1**–**Co5**; Figures S5 and S6: Comparison of activity and molecular weight at different temperatures (a) and reaction time (b) using MAO/MMAO catalytic system; Figure S7: GPC curves at different Al/Co ratio, temperatures and reaction time using MMAO catalytic system; Figure S8: DSC curves of polyethylene samples obtained at different conditions; Figures S9 and S10: ^1^H NMR and ^13^C NMR spectra of polymer produced by **Co4**/MMAO under optical conditions. Table S1:Crystal data and structural refinements for **Co1**, **Co2**, **Co4** and **Co5**. Refs [44,45] are cited in the Supplementary Materials.
