# Supplementary material for: Finely-Tuned Bis(imino)pyridylcobalt Complexes Enhance Ethylene Polymerization: The Role of Bulky and Halogen Substituents
Source: Molecules. 2025 Feb 13;30(4):859. doi: 10.3390/molecules30040859 (PMC11857908; doi:10.3390/molecules30040859)
Supplement: Supplementary file 1 [file molecules-30-00859-s001.zip › molecules-3469650-supplementary.pdf]

Supporting Information

Finely-Tuned Bis(imino)pyridylcobalt Complexes Enhance Ethylene Polymerization: The Role of Bulky and Halogen Substituents

Elizabeth Ogbe [1,2], Yanping Ma [1,*], Yizhou Wang [1,2], Jiahao Gao [1], Yang Sun [1] and Wen-Hua Sun [1,2,*]

[1] Key Laboratory of Engineering Plastics and Beijing National Laboratory for Molecular Science, Institute of Chemistry, Chinese Academy of Sciences, Beijing 100190, China.

[2] CAS Research/Education Center for Excellence in Molecular Sciences and International School, University of Chinese Academy of Sciences, Beijing 100049, China.

Correspondence: myanping@iccas.ac.cn (Y.M.); whsun@iccas.ac.cn (W.-H.S.)

Table of Contents

1	X-ray crystallographic studies
2	General Considerations
3	General procedure for ethylene polymerization at 1, 5 and 10 atm
Table S1	Crystal data and structural refinements for **Co1**, **Co2**, **Co4** and **Co5**
Figure S1	^{1}H NMR and ^{13}C NMR spectra of Sterically Hindered Aniline **X3** and **X4**
Figure S2	^{1}H NMR and ^{13}C NMR spectra of imino-ketone and ligands **L1-L5**
Figure S3	^{19}F NMR spectra of Ligands
Figure S4	FT-IR spectra of ligands **L1-L5** and Complexes **Co1-Co5**
Figures S5-S6	Comparison of activity and molecular weight at different temperatures (a) and reaction time (b) using MAO/MMAO catalytic system
Figures S7	GPC curves at different Al/Co ratio, temperatures and reaction time using MMAO catalytic system
Figures S8	DSC curves of polyethylene samples obtained at different conditions
Figures S9-10	^{1}H NMR and ^{13}C NMR spectra of polymer produced by Co4/MMAO under optical conditions

1. X-ray Crystallographic Studies

Single crystal X-ray diffraction analysis of **Co2** and, **Co4** was carried out using a Rigaku Sealed Tube CCD (Saturn 724+) diffractometer. This diffractometer utilized graphite-monochromated Cu-Kα radiation with a wavelength (λ) of 1.54184 Å while **Co1** and **Co5** were measured with Mo radiation.The measurements were conducted at a temperature of 170 (±10) K. The determination of cell parameters involved the global refinement of the positions of all collected reflections. Intensities obtained from the X-ray diffraction analysis were corrected for Lorentz and polymerization effects, and an empirical absorption correction was applied. The structure of complex **Co1**, **Co2**, **Co4** and **Co5** was solved using direct methods and subsequently refined through full-matrix least squares fitting on **Co1**, **Co2**, **Co4** and **Co5**. Non- hydrogen atoms in each complex were refined with directional refinement, while the positions of all hydrogen atoms were determined based on calculated positions. Data collected during the analysis were processed using the Olex[1] program, the structure was determined using the SHELXT[2] program through inherent phasing and subsequently refined using the SHELXL [2] suite via Least Squares minimization. The crystallographic data and and structure refinement parameters for **Co1**, **Co2**, **Co4** and **Co5** are presented in the table below.

2. General Considerations

All reactions involving air- and moisture-sensitive compounds were conducted using standard Schlenk techniques under inert nitrogen atmospheres. Toluene was refluxed with sodium under a nitrogen environment and then distilled before use. The aluminum alkyls, methylaluminoxane (MAO, 1.46 M in toluene), and modified methylaluminoxane (MMAO, 1.93 M in n-heptane) were obtained from Akzo Nobel Corporation (Nanjing, China). High purity ethylene (C_2H_4) was sourced from Beijing Yansan Petrochemical Co. (Beijing, China) and used without additional purification. Additional reagents were purchased from Acros (Beijing, China), Aldrich (Beijing, China), or local suppliers in Beijing, China. The FT-IR spectra were recorded on the Perkin Elmer Spectrum 2000 FT-IR spectrometer (Shanghai, China). The ^{1}H and ^{13}C NMR spectra of all bis(imino)pyridines and their precursors were recorded on a Bruker DMX 400 MHz NMR (Bruker, Karlsruhe, Germany) at ambient temperature using TMS as an internal standard. The elemental analyses were performed on a Flash EA 1112 microanalyzer (Thermo Electron SPA, Beijing, China). The molecular weight (M_w) and dispersity (M_w/M_n) of the polyethylenes were determined using a PL-GPC 220 instrument (PL, Shropshire, UK) operating at 150 °C and employing 1,2,4-trichlorobenzene as the eluting solvent. The melting points of the polyethylenes were measured using differential scanning calorimetry (Q2000 DSC; TA Instruments, New Castle, DE, USA) under a nitrogen atmosphere. Typically, a 5.0 mg sample of polyethylene (PE) was heated to 160 °C at a heating rate of 20 °C min^{-1} and held at 160 °C for 3 minutes to eliminate any thermal history. The sample was then cooled to -40 °C at a rate of 20 °C min^{-1}. The ^{1}H NMR spectra of the polyethylenes were obtained using a Bruker AVANCE III 500 MHz instrument operating at 100 °C. Sample preparation generally involved taking a measured quantity of polyethylene (80-100 mg) and dissolving it in deuterated 1,1,2,2-tetrachloroethane at high temperature. Then, a portion of the solution was transferred to a 5 mm standard glass NMR tube.

3. General procedure for ethylene polymerization (1, 5 and 10 atm pressure)

These experiments were conducted using a stainless-steel autoclave (250 mL) equipped with a mechanical stirrer and a control system for ethylene pressure and temperature. In a typical procedure, the autoclave was emptied and then filled with nitrogen gas. This process was repeated three times. After the final emptying, ethylene was introduced to create an ethylene atmosphere inside the autoclave. At this point, a solution of the corresponding precatalyst (2 μmol) in toluene (25 mL) was injected, followed by an additional 25 mL of toluene. Then, the appropriate amount of co-catalyst (MAO or MMAO) was added, followed by another 50 mL of toluene. Finally, the autoclave was pressurized with ethylene (5 or 10 atm) and stirring began at a speed of 400 rpm. Once the reaction was complete, the stirring was stopped, the reactor was cooled to room temperature, and the pressure was slowly released. The contents of the autoclave were treated with hydrochloric acid (10%) in ethanol to stop the reaction, and the resulting polymer was washed with ethanol. Finally, the polymer was filtered, dried under reduced pressure at 40 °C, and weighed.

The polymerizations were conducted in a Schlenk vessel at a pressure of 1 atm using C_2H_4. First, the vessel was filled with an atmosphere of ethylene (approximately 1 atm). Then, 2μmol of cobalt precatalysts was added to the vessel, followed by 30 mL of toluene. The required amount of co-catalyst was introduced using a syringe. The resulting solution was stirred at 30 °C under an ethylene atmosphere (1 atm). After 30 minutes, the pressure was gradually released, and the solution was quenched with 10% hydrochloric acid in ethanol. The polymer was washed with ethanol, dried under reduced pressure at 40 °C, and weighed.

References

1. Dolomanov O V., Bourhis LJ, Gildea RJ, et al. OLEX2: A complete structure solution, refinement and analysis program. J Appl Crystallogr 2009;42(2); doi: 10.1107/S0021889808042726.
2. Sheldrick GM. SHELXT - Integrated space-group and crystal-structure determination. Acta Crystallogr A 2015;71(1); doi: 10.1107/S2053273314026370.

Table S1: Details of the crystal data and structure refinement parameters for **Co1**, **Co2**, **Co4** and **Co5**

**Identification code**	**Co1**	**Co2**	**Co4**	**Co5**
CCDC No.	2417868	2417869	2417870	2417871
Empirical formula	$C_{50}H_{40}Cl_3CoF_4N_3$	$C_{55}H_{50}Cl_9CoF_4N_3$	$C_{54}H_{48}Cl_9CoF_4N3$	$C_{53}H_{46}Cl_3CoF_4N_3$
Formula weight	924.13	1206.96	1192.93	966.21
Temperature/K	170.00(10)	170(2)	170(2)	170.00(10)
Crystal system	Monoclinic	Monoclinic	monoclinic	Orthorhombic
Space group	P21/c	P21/c	P21/c	P212121
a/Å	20.7469(5)	12.82705(15)	11.84909(14)	12.8771(8)
b/Å	15.0555(3)	15.94030(16)	15.6133(2)	15.8643(8)
c/Å	15.3159(3)	27.4342(4)	29.5476(4)	26.118(2)
α/°	90	90	90	90
β/°	96.821(2)	94.4212(12)	95.6223(12)	90
γ/°	90	90	90	90
Volume/$Å^3$	4750.14(18)	5592.70(11)	5440.09(12)	5335.5(6)
Z	4	4	4	4
ρcalcg/cm^3	1.292	1.433	1.457	1.203
μ/mm^{-1}	0.582	6.798	6.982	0.521
F(000)	1900	2468	2436	1996
Crystal size/mm^3	0.25 × 0.18 × 0.1	0.4 × 0.3 × 0.2	0.25 × 0.15 × 0.1	0.25 × 0.2 × 0.05
Radiation	Mo Kα (λ = 0.71073)	CuKα (λ = 1.54184)	CuKα (λ = 1.54184)	Mo Kα (λ = 0.71073)
2Θ range for data collection/°	3.35 to 61.924	6.418 to 155.768	6.012 to 155.754	3.004 to 62.284
Index ranges	-25 ⩽ h ⩽ 30, -14 ⩽ k ⩽ 21, -20 ⩽ l ⩽ 21	-16 ⩽ h ⩽ 14, -20 ⩽ k ⩽14, -34 ⩽ l ⩽ 34	-14 ⩽ h ⩽ 14, -19 ⩽ k ⩽ 19, -37 ⩽ l ⩽ 35	-18 ⩽ h ⩽ 18, -19 ⩽ k ⩽ 22, -36 ⩽ l ⩽ 29
Reflections collected	39437	41906	40843	41115
Independent reflections	12763 [Rint = 0.0255, Rsigma = 0.0319]	11534 [Rint = 0.0548, Rsigma = 0.0445]	11204 [Rint =0.0403, R_{sigma} = 0.0330]	14418 [Rint = 0.1399, R_{sigma} = 0.1236]
Data/restraints/parameters	12763/1/565	11534/1/665	11204/0/647	14418/108/584
Goodness-of-fit on F^2	1.029	1.081	1.016	1.214
Final R indexes [I>=2σ (I)]	R1 = 0.0443, wR2 = 0.1081	R1 = 0.1071, wR2 = 0.3122	R_1 = 0.0445, wR_2 = 0.0992	R_1 = 0.1331, wR_2 = 0.3355
Final R indexes [all data]	R1 = 0.0590, wR2 = 0.1135	R1 = 0.1151, wR2 = 0.3172	R_1 = 0.0547, wR_2 = 0.1044	R_1 = 0.1711, wR_2 = 0.3604
Largest diff. peak/hole / e Å-3	0.53/-0.27	1.17/-0.95	0.55/-0.62	2.97/-0.99

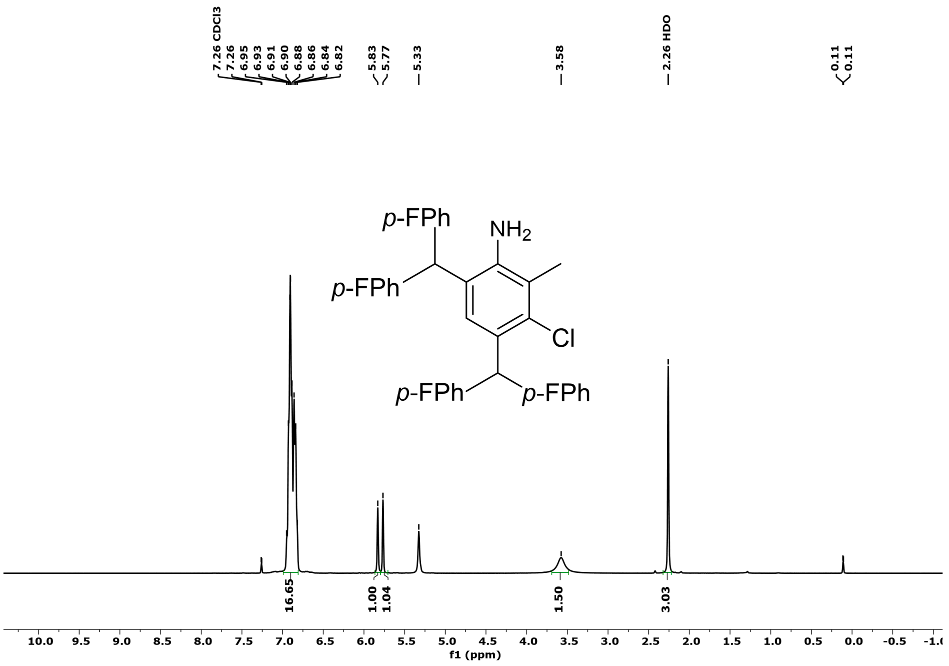

Figure S1-1: The ¹H NMR spectrum of Sterically Hindered Aniline (**X3**) in $CDCl_3$

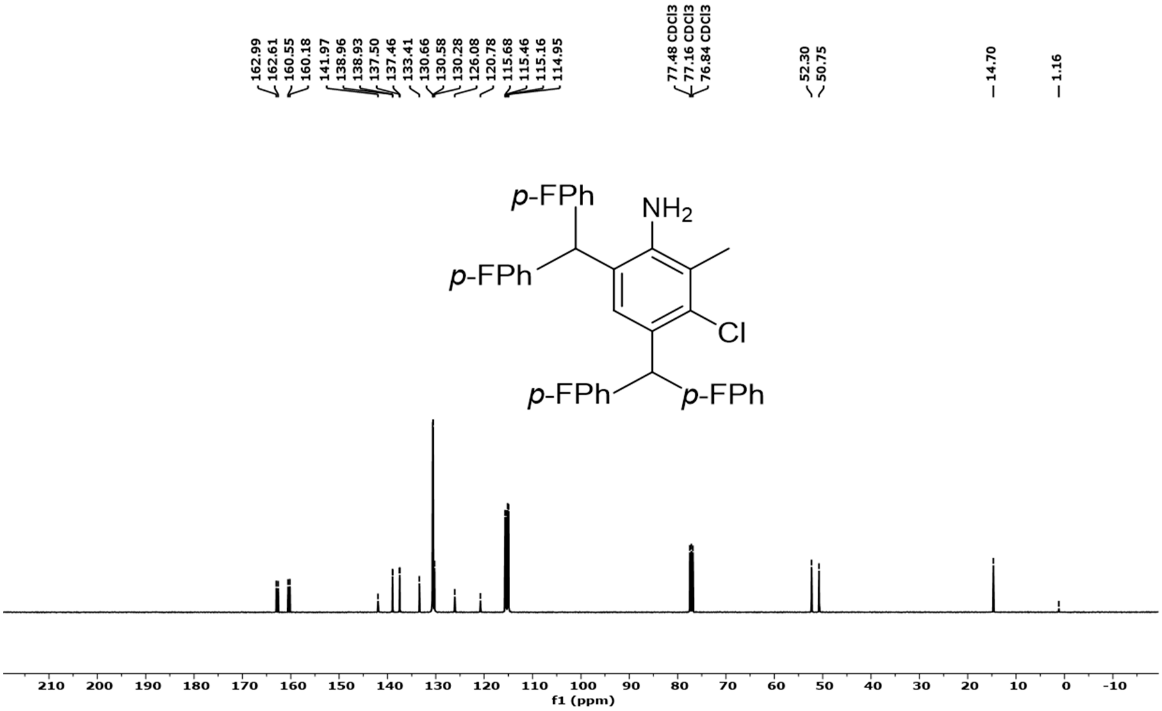

Figure S1-2: The ¹³C NMR spectrum of Sterically Hindered Aniline **(X3**) in $CDCl_3$

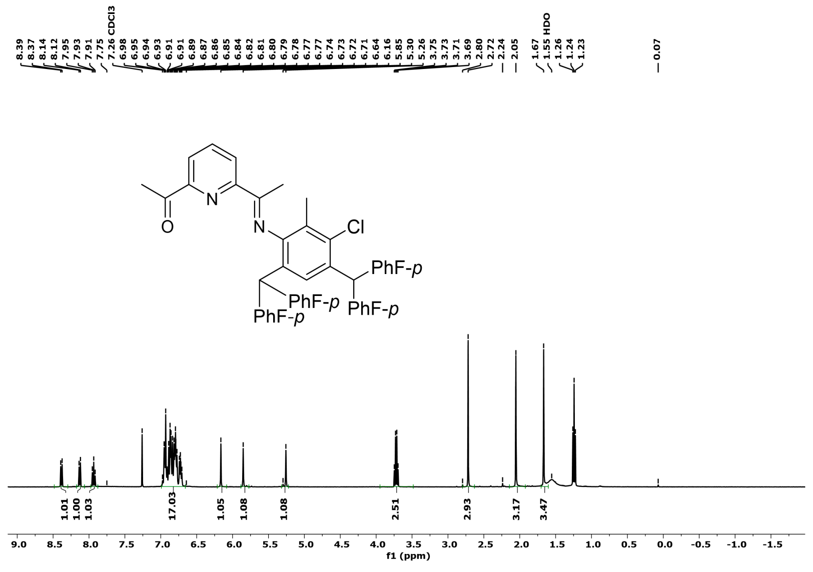

Figure S1-3: The ^{1}H NMR spectrum of Imino-ketone (**X4**) in $CDCl_3$

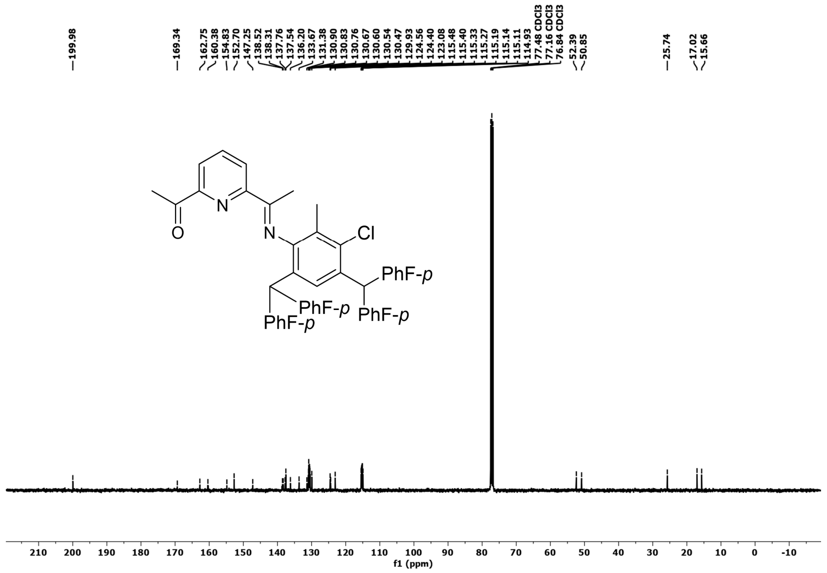

Figure S1-4: The ^{13}C NMR spectrum of Imino-ketone (**X4**) in $CDCl_3$

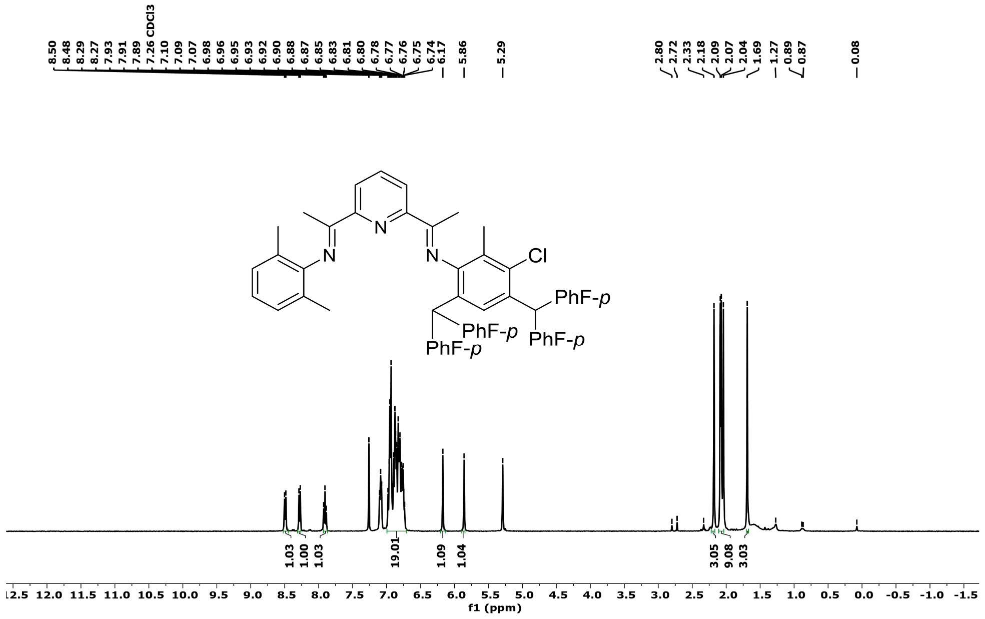

Figure S2-1: The ^{1}H NMR spectrum of **L1** (Ar=2,6-$Me_2C_6H_3$) in $CDCl_3$

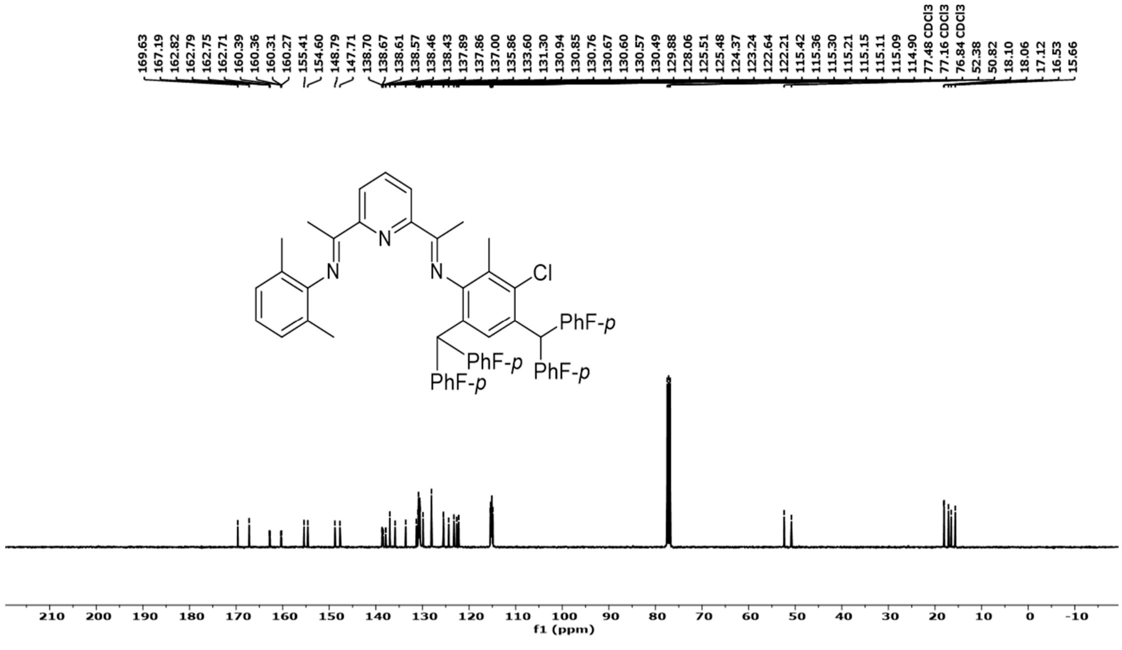

Figure S2-2: The ^{13}C NMR spectrum of **L1** (Ar=2,6-$Me_2C_6H_3$) in $CDCl_3$

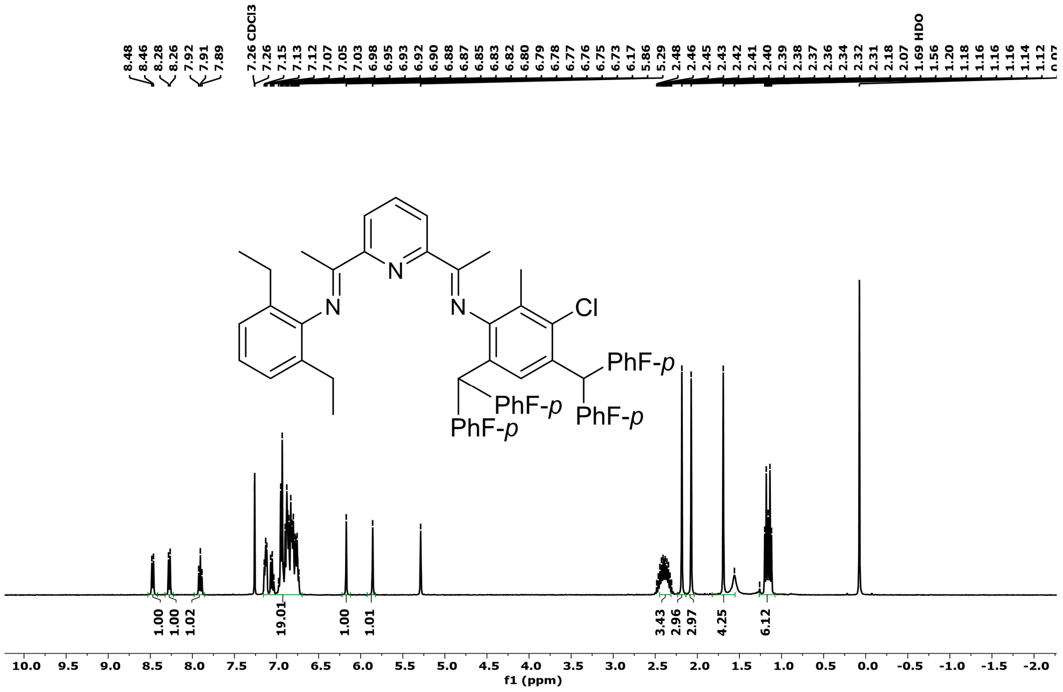

Figure S2-3: The ^{1}H NMR spectrum of **L2** (2,6-$Et_2C_6H_3$) in $CDCl_3$

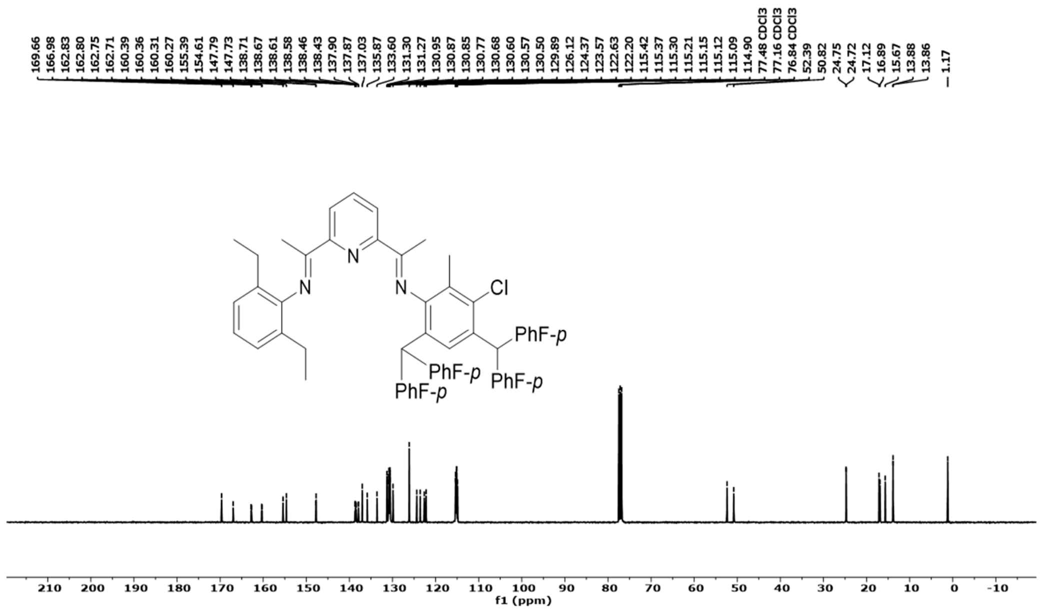

Figure S2-4: The ^{13}C NMR spectrum of **L2** (2,6-$Et_2C_6H_3$) in $CDCl_3$

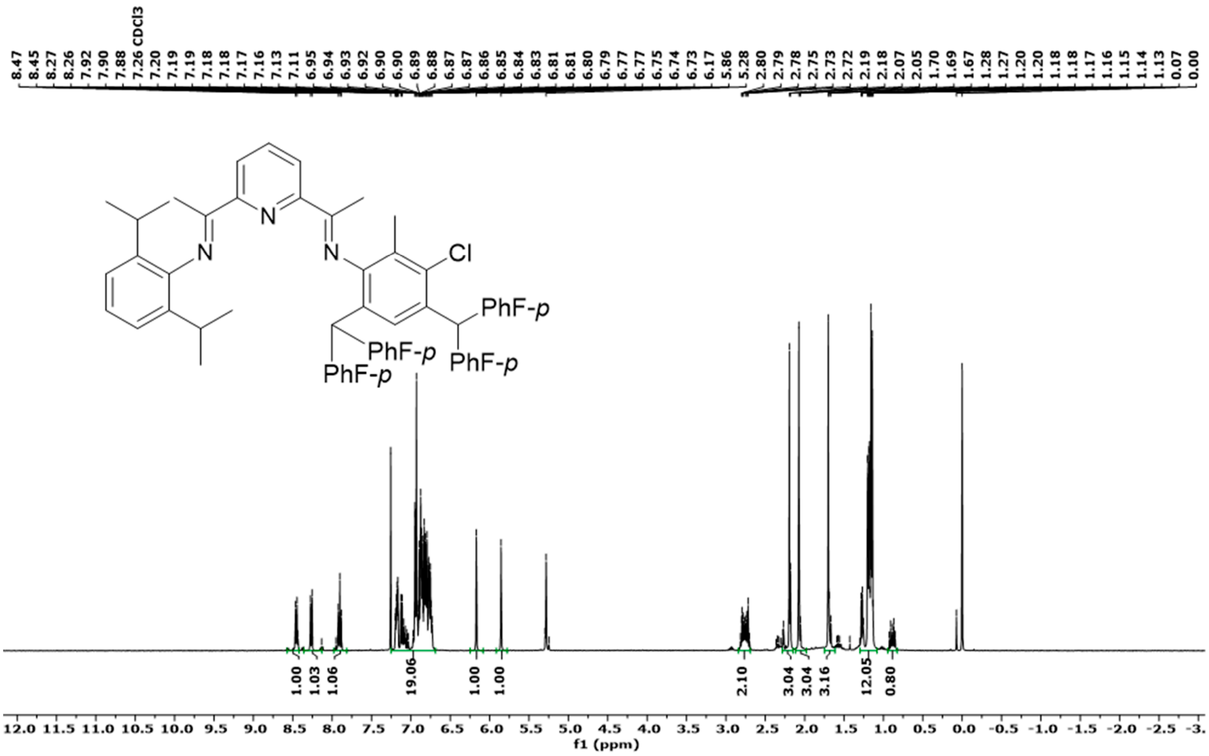

Figure S2-5: The ^{1}H NMR spectrum of **L3** (2,6-iPr$_2$C$_6$H$_3$) in $CDCl_3$

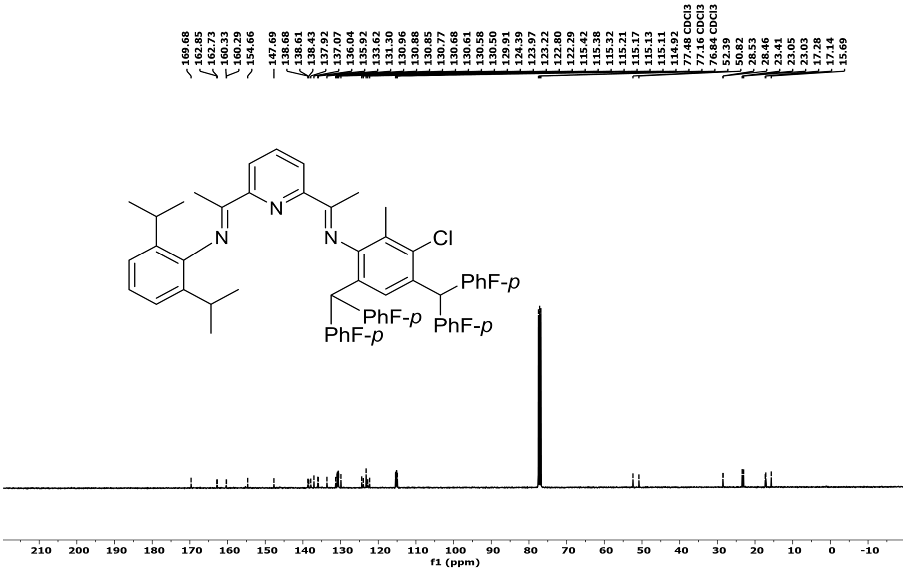

Figure S2-6: The ^{13}C NMR spectrum of **L3** (2,6-*i*Pr$_2$C$_6$H$_3$) in $CDCl_3$

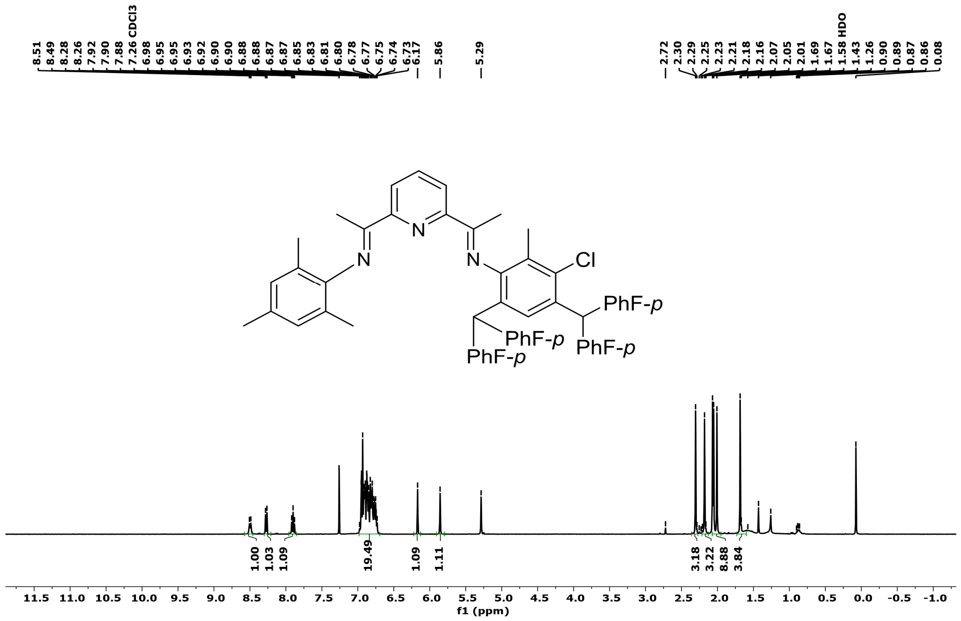

Figure S2-7: The ^{1}H NMR spectrum of **L4** (2,4,6-$Me_3C_6H_2$) in $CDCl_3$

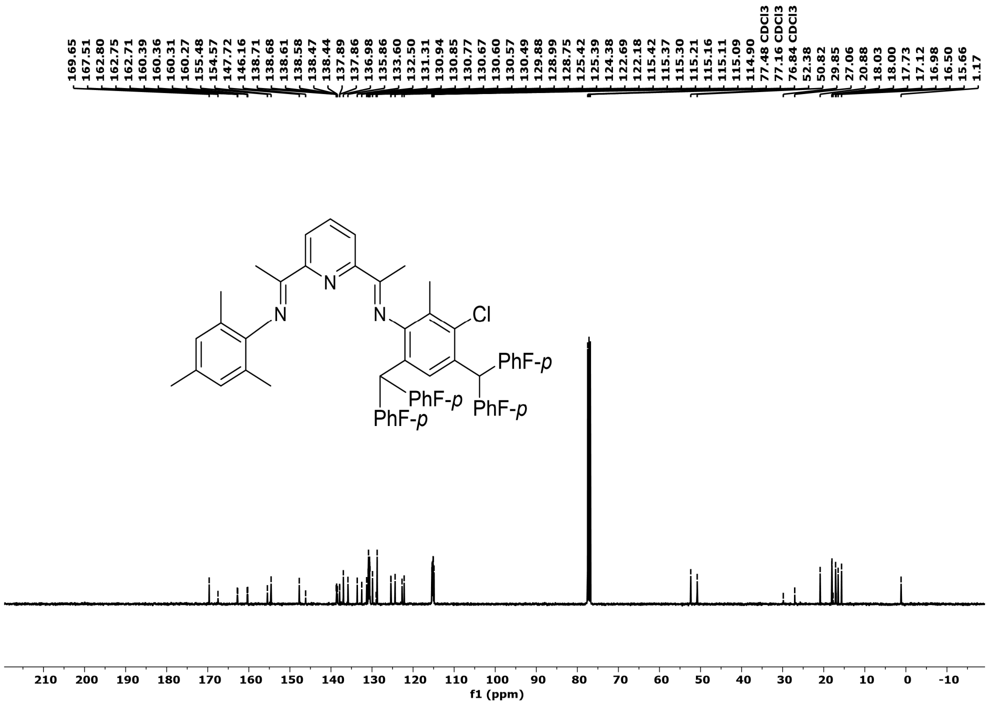

Figure S2-8: The ^{13}C NMR spectrum of **L4** (2,4,6-$Me_3C_6H_2$) in $CDCl_3$

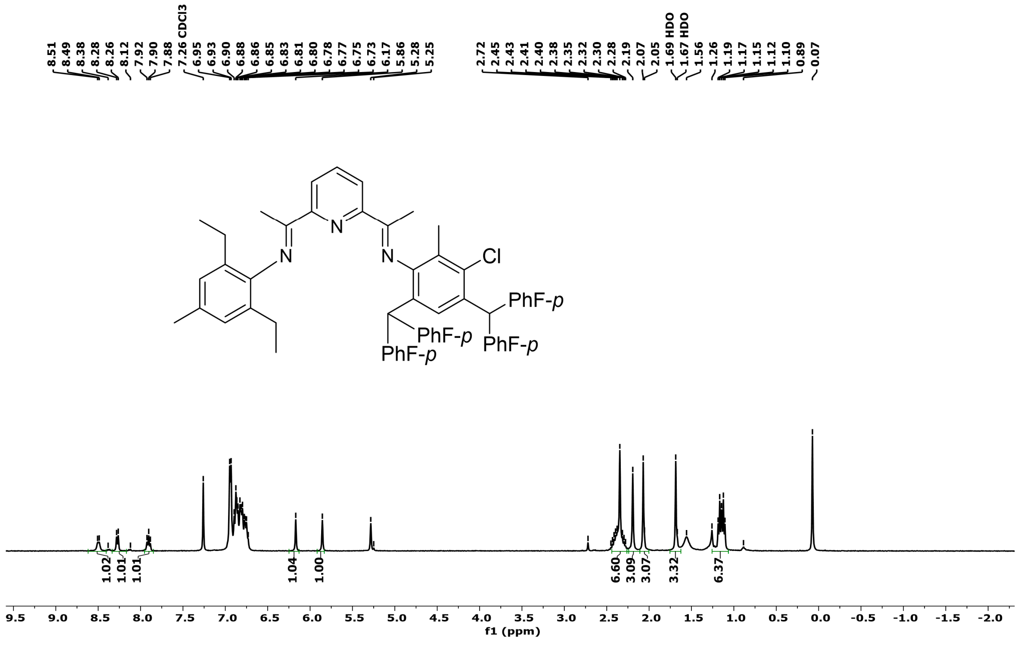

Figure S2-9: The ^{1}H NMR spectrum of **L5** (2,6-Et_2-4-MeC_6H_2) in $CDCl_3$

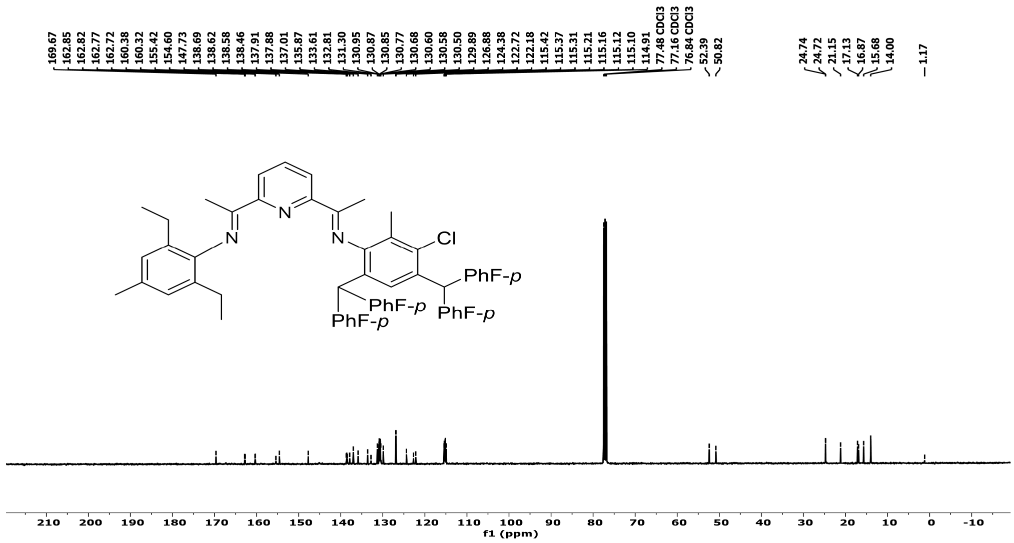

Figure S2-10: The ^{13}C NMR spectrum of **L5** (2,6-Et_2-4-MeC_6H_2) in $CDCl_3$

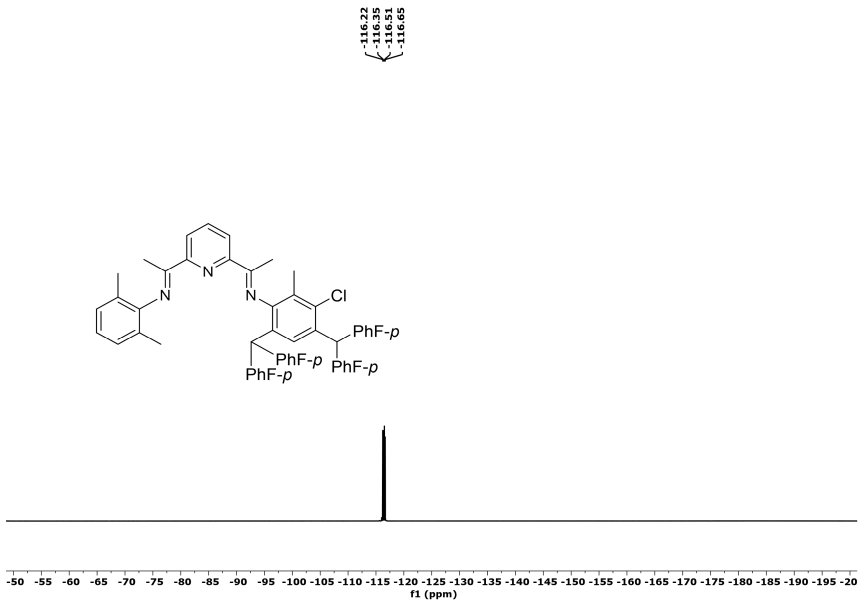

Figure S3-1. ^{19}F NMR spectra of **L1**, recorded in $CDCl_3$ at ambient temperature.

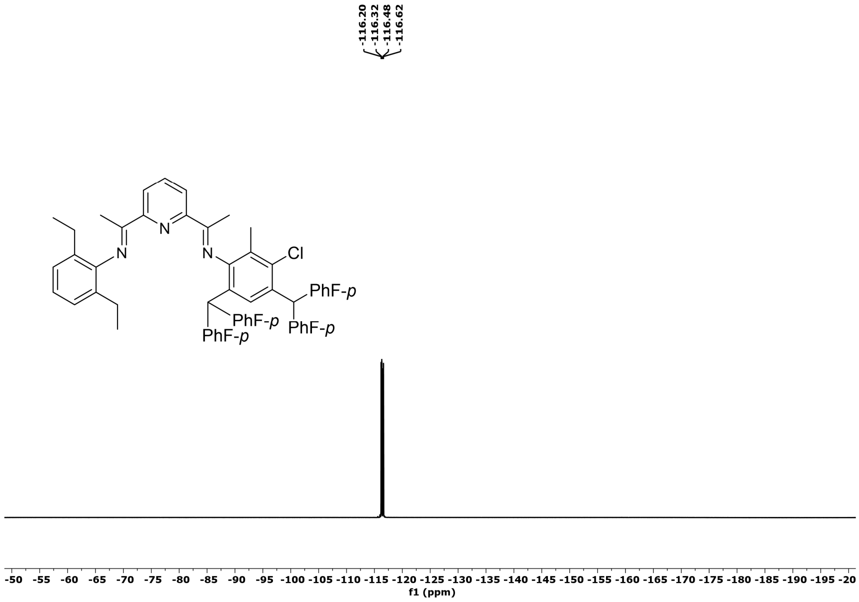

Figure S3-2. ^{19}F NMR spectra of **L2**, recorded in $CDCl_3$ at ambient temperature.

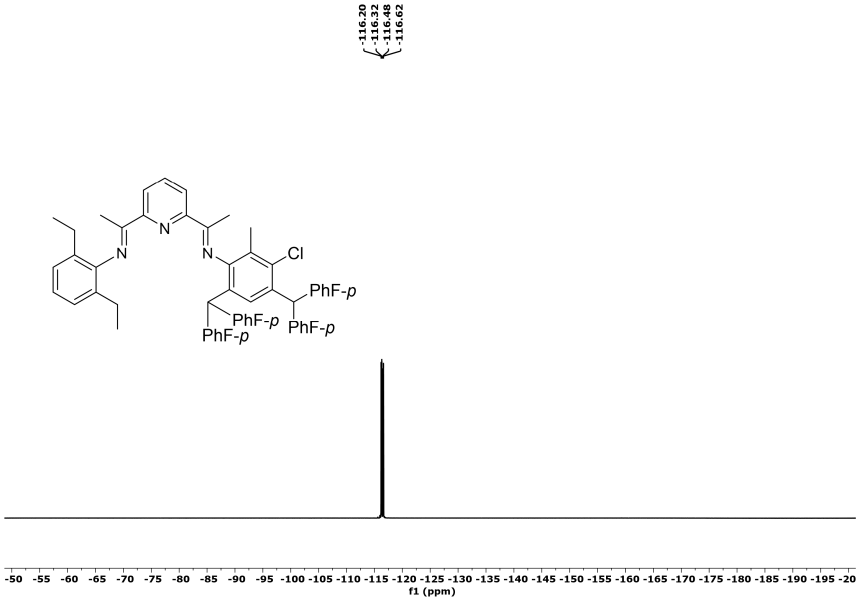

Figure S3-3. ^{19}F NMR spectra of **L3**, recorded in $CDCl_3$ at ambient temperature.

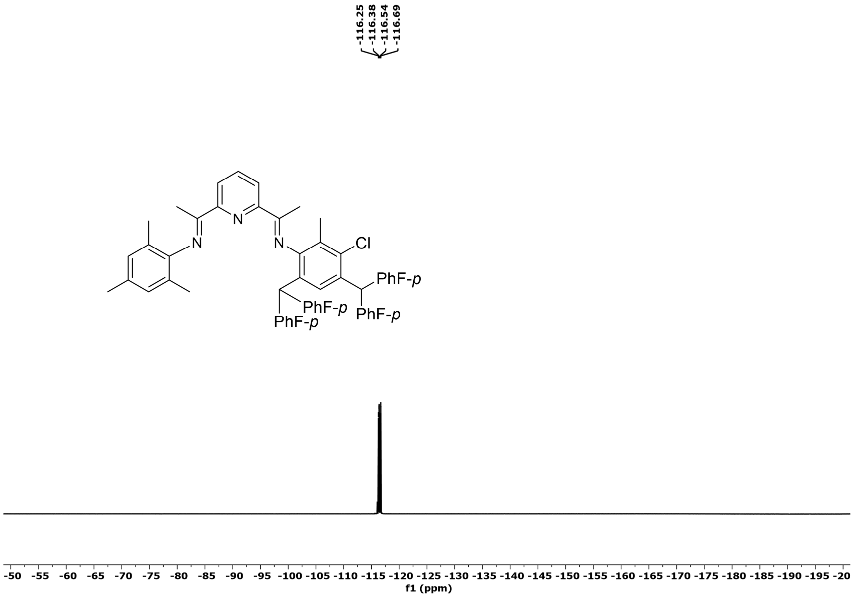

Figure S3-4. ^{19}F NMR spectra of **L4**, recorded in $CDCl_3$ at ambient temperature.

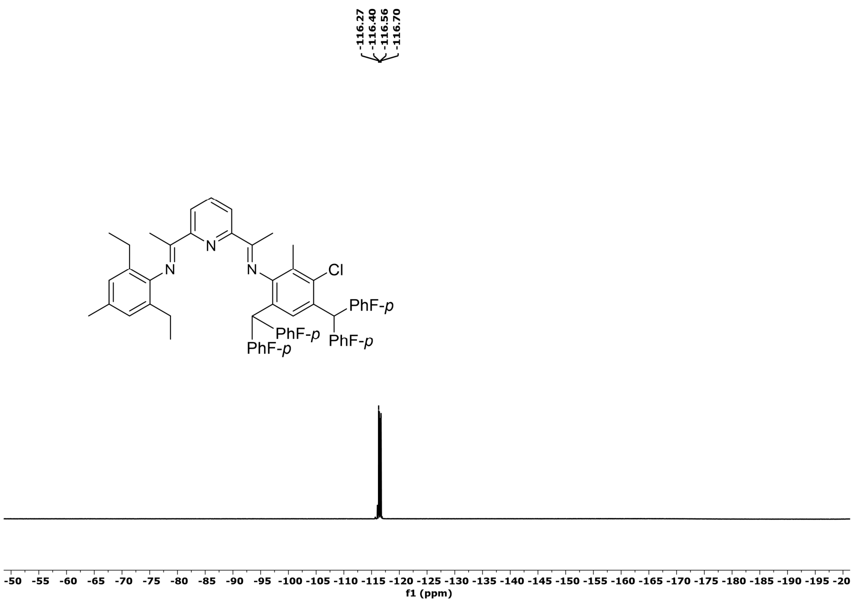

Figure S3-5. ^{19}F NMR spectra of **L5**, recorded in $CDCl_3$ at ambient temperature.

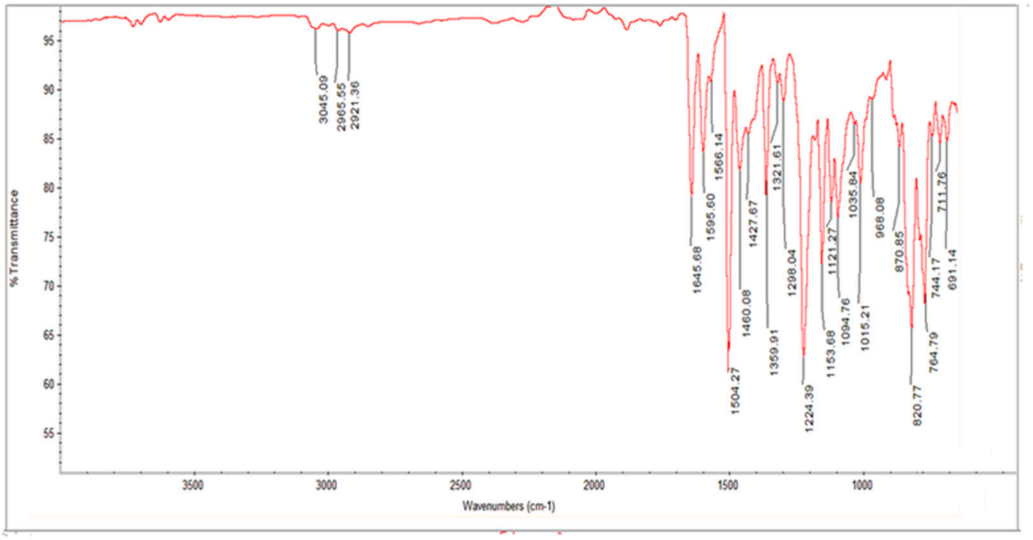

Figure S4-1: FT-IR spectrum of **L1** (Ar=2,6-$Me_2C_6H_3$)

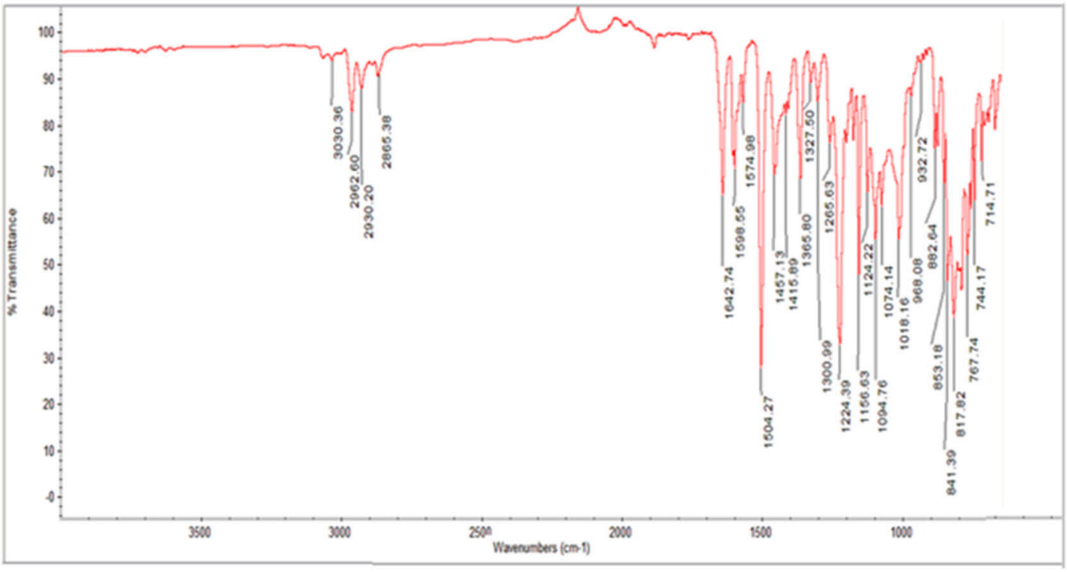

Figure S4-2: FT-IR spectrum of **L2** ($2,6\text{-}Et_2C_6H_3$)

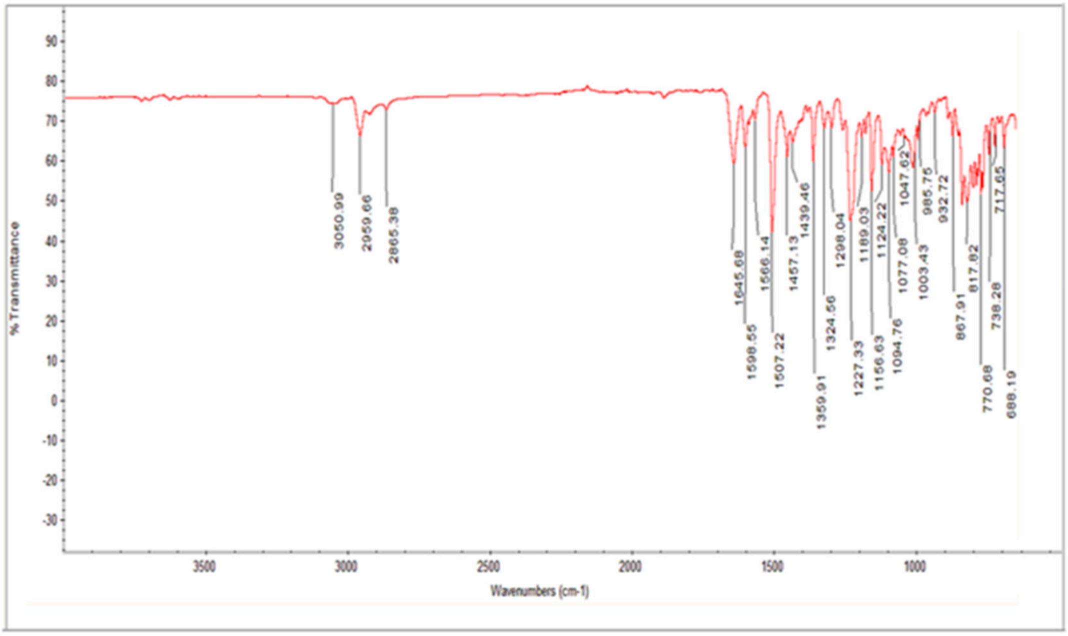

Figure S4-3: FT-IR spectrum of **L3** ($2,6\text{-}iPr_2C_6H_3$)

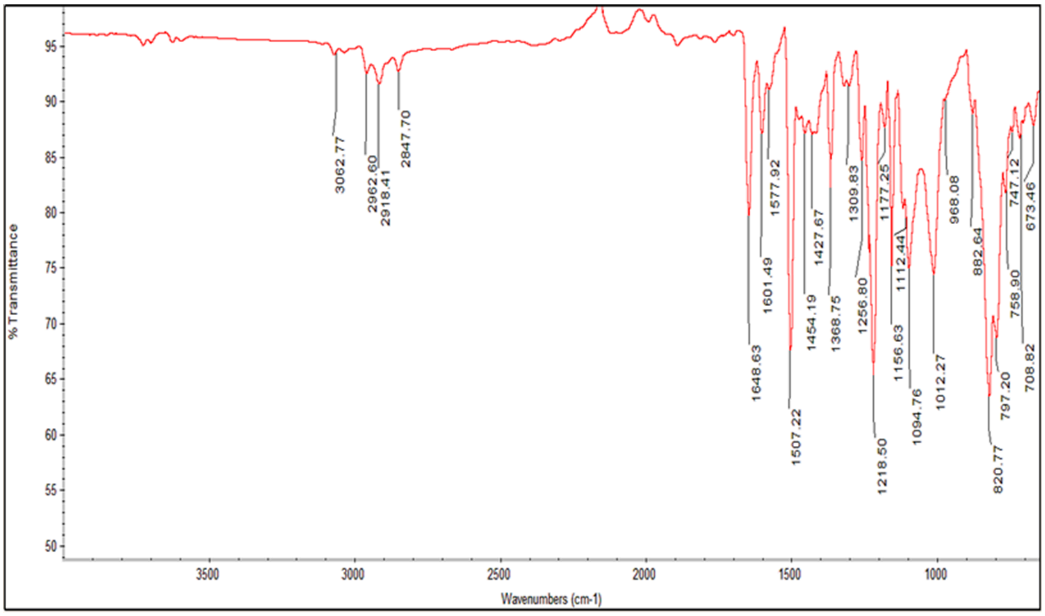

Figure S4-4: FT-IR spectrum of **L4** (2,4,6-$Me_3C_6H_2$)

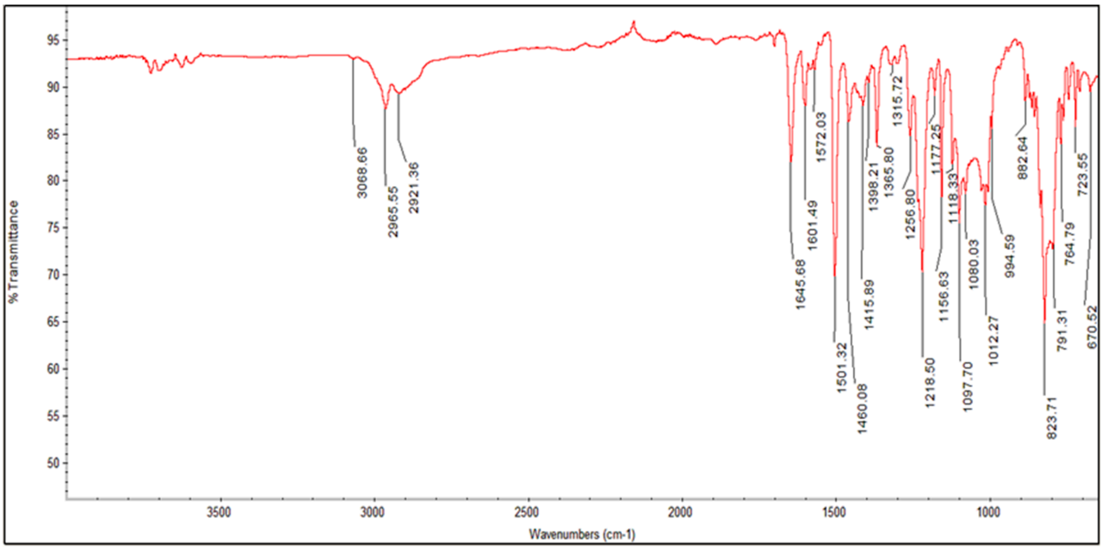

Figure S4-5: FT-IR spectrum of **L5** (Ar=2,6-Et_2-4-MeC_6H_2)

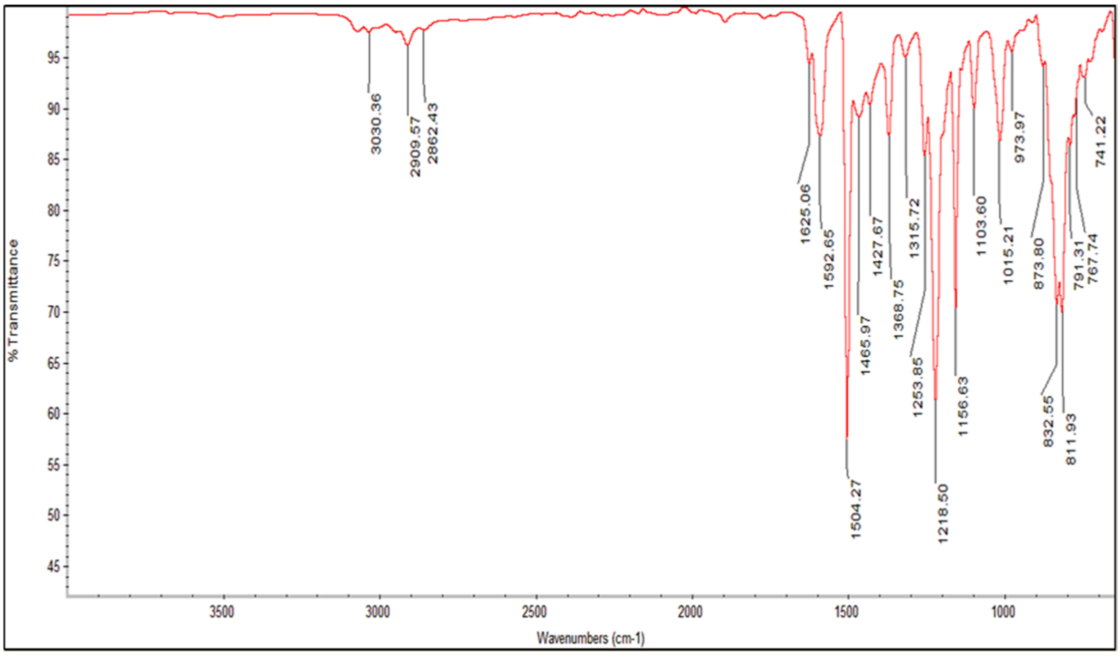

Figure S4-6: FT-IR spectrum of **Co1** (Ar=2,6-$Me_2C_6H_3$)

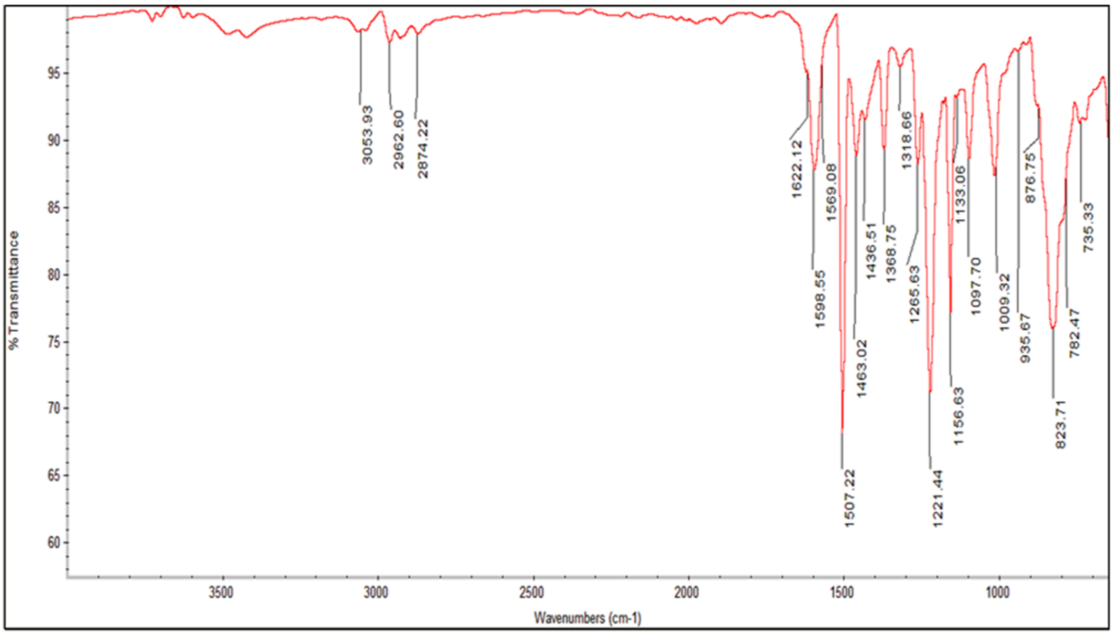

Figure S4-7: FT-IR spectrum of **Co2** (Ar=2,6-$Et_2C_6H_3$)

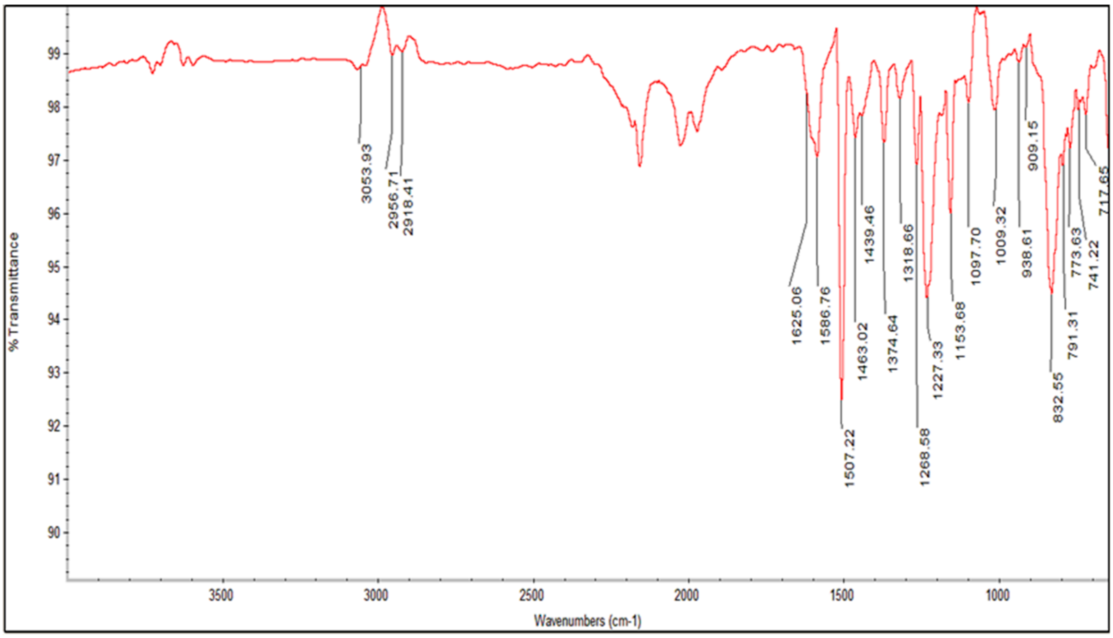

Figure S4-8: FT-IR spectrum of **Co3**(Ar=2,6-i-$Pr_2C_6H_3$)

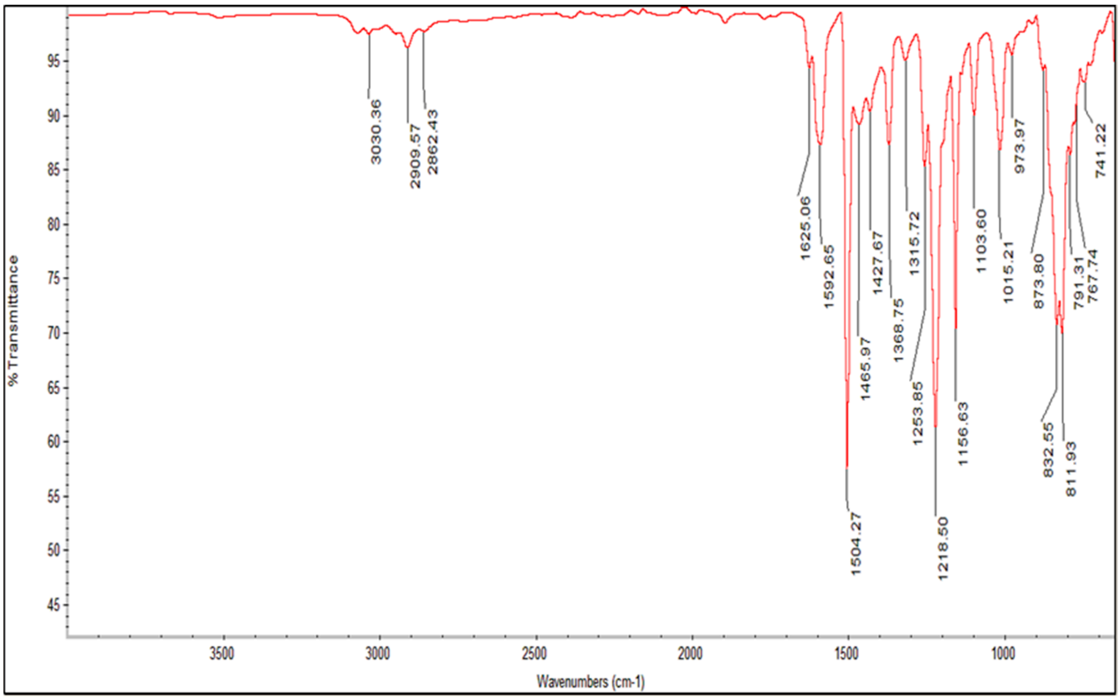

Figure S4-9: FT-IR spectrum of **Co4** (Ar=2,4,6-$Me_3C_6H_2$)

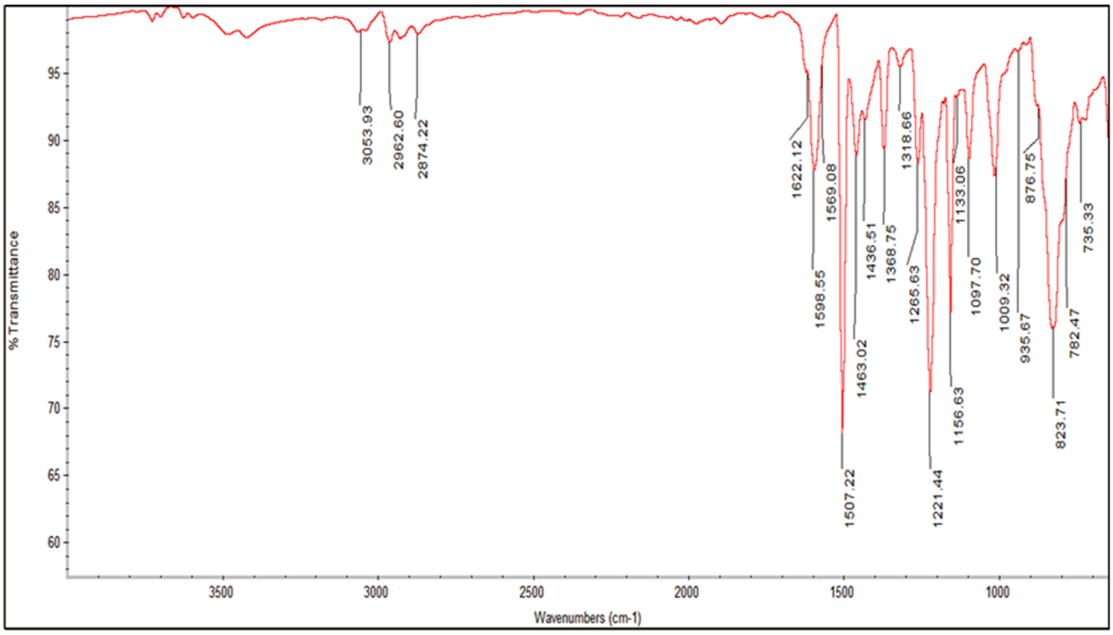

Figure S4-10: FT-IR spectrum of **Co5** (Ar = 2,6-Et_2-4-MeC_6H_2)

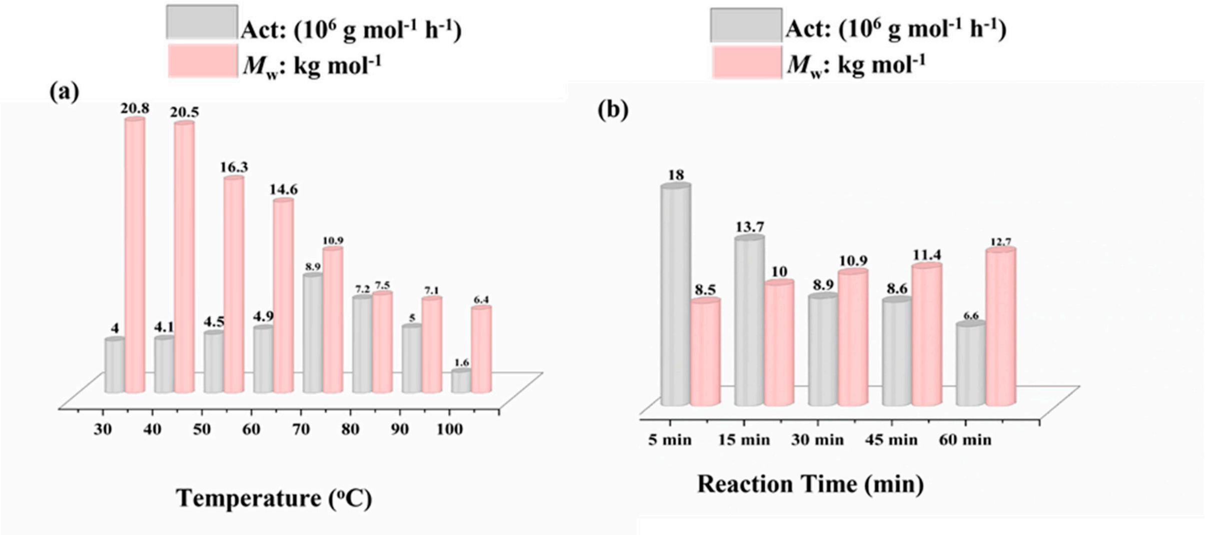

Figure S5: Catalytic activity and polymer molecular weights at different temperatures (a), and reaction times (b) using **Co4/MAO** system (**Table 2**).

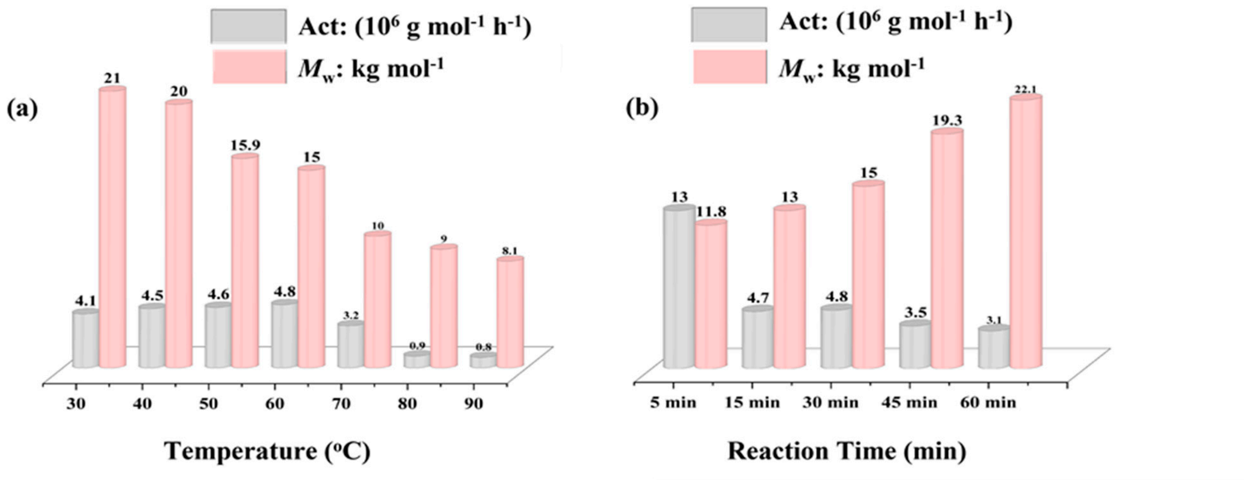

Figure S6: Catalytic activity and polymer molecular weights at different temperatures (a), and reaction times (b) using **Co4/MMAO** system (**Table 3**)

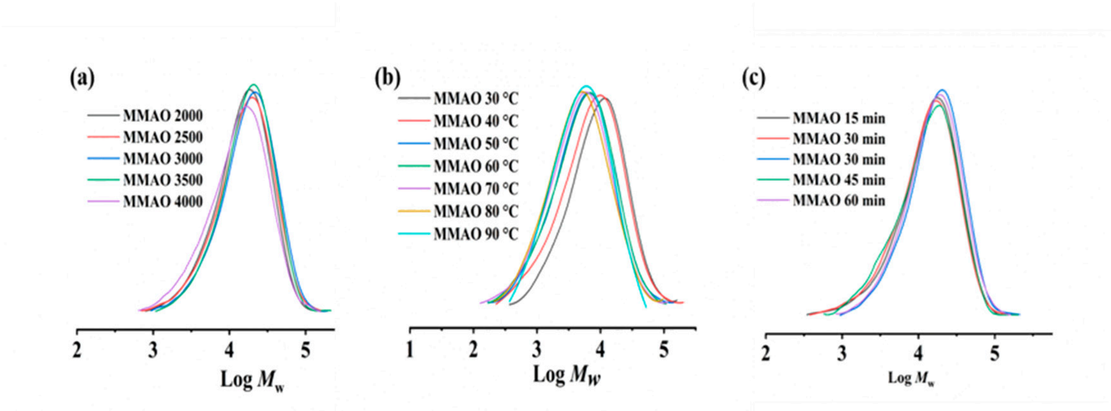

Figure S7: The GPC curves indicating Log M_w for the polyethylene produced using **Co4/MMAO** as a function of Al:Co molar ratio(a), different temperature (b) and reaction time (c) (entries 1-5, **Table 3**)

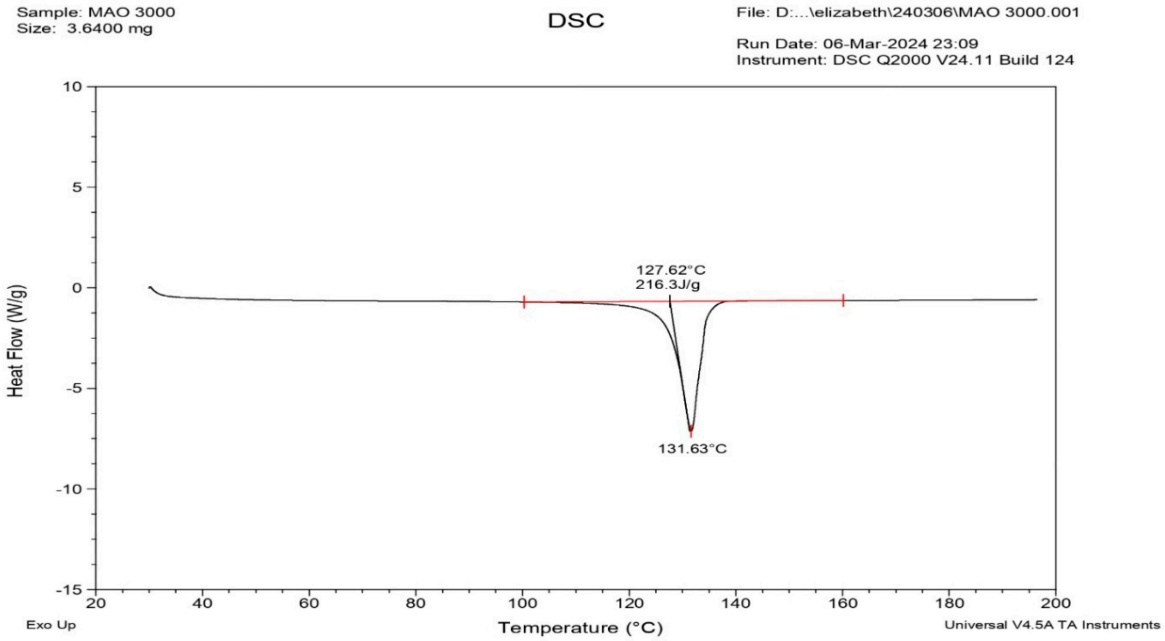

Figure S8-1: DSC curve indicating the T_m of PE prepared by **Co4/MAO** (**Table 2**, entry 4)

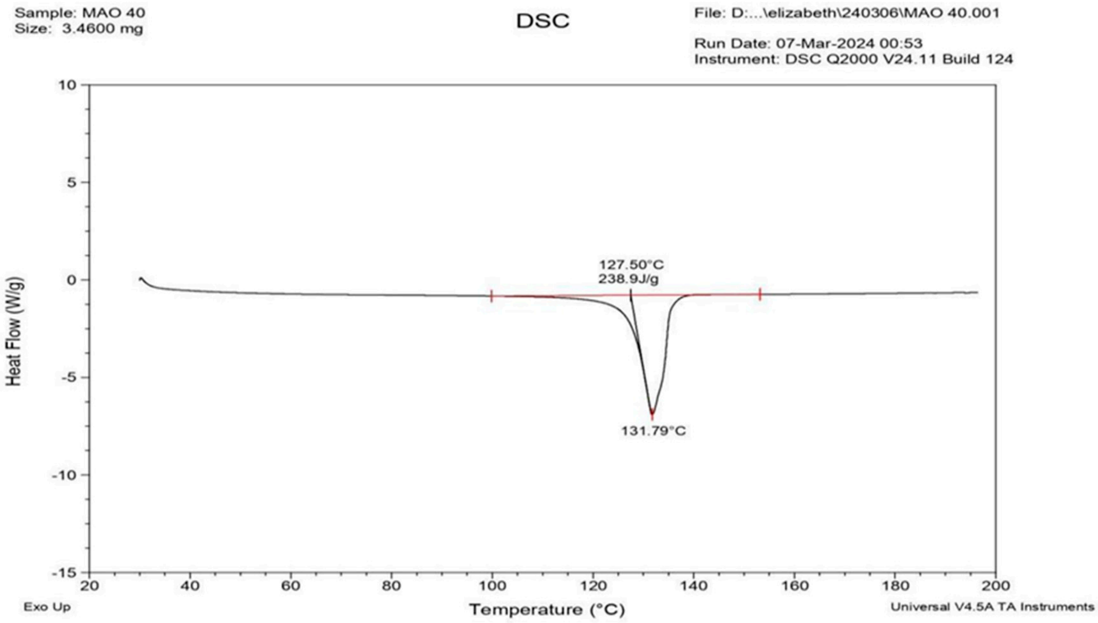

Figure S8-2: DSC curve indicating the T_m of PE prepared by **Co4/MAO** (**Table 2**, entry 6)

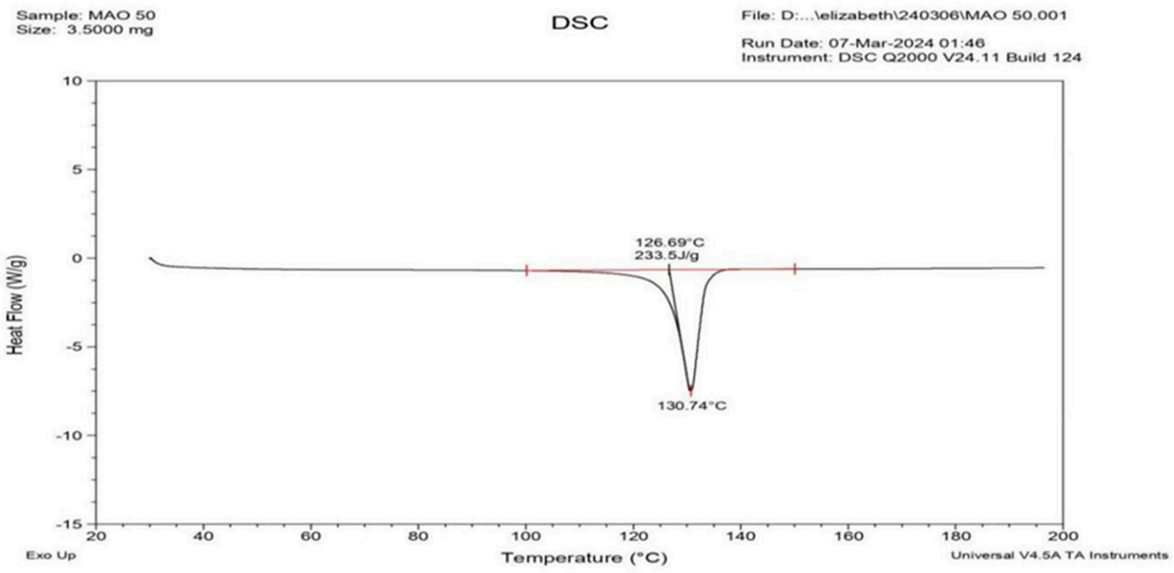

Figure S8-3: DSC curve indicating T_m of PE prepared by **Co4/MAO** (**Table 2**, entry 7)

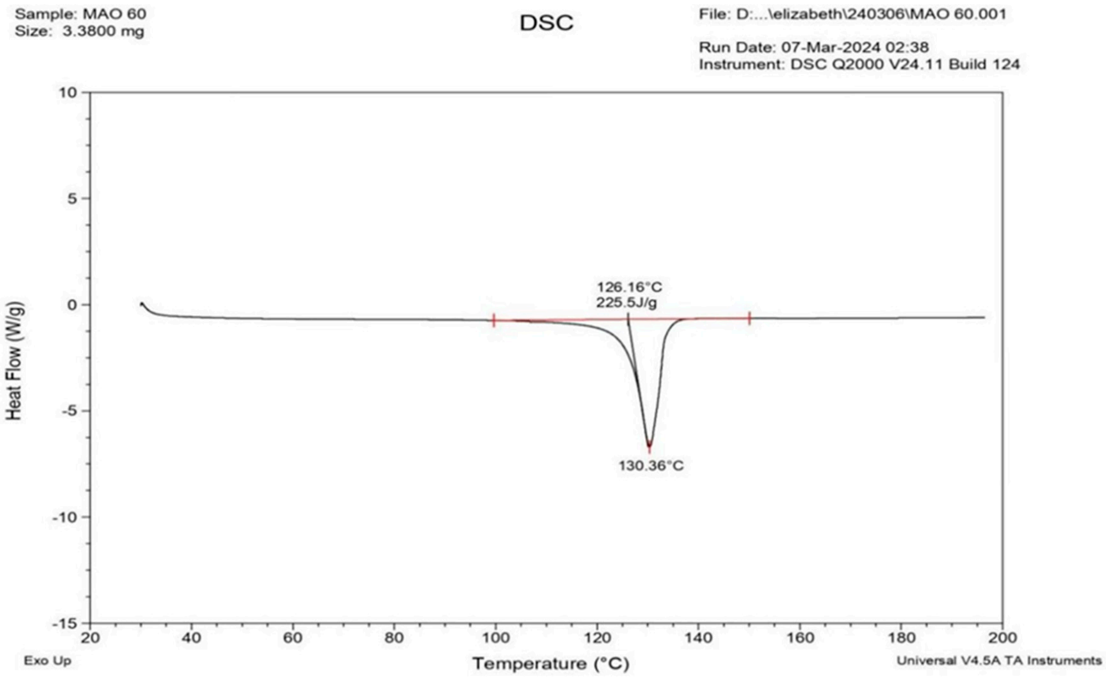

Figure S8-4: DSC curve indicating the T_m of PE prepared by **Co4/MAO** (**Table 2**, entry 8)

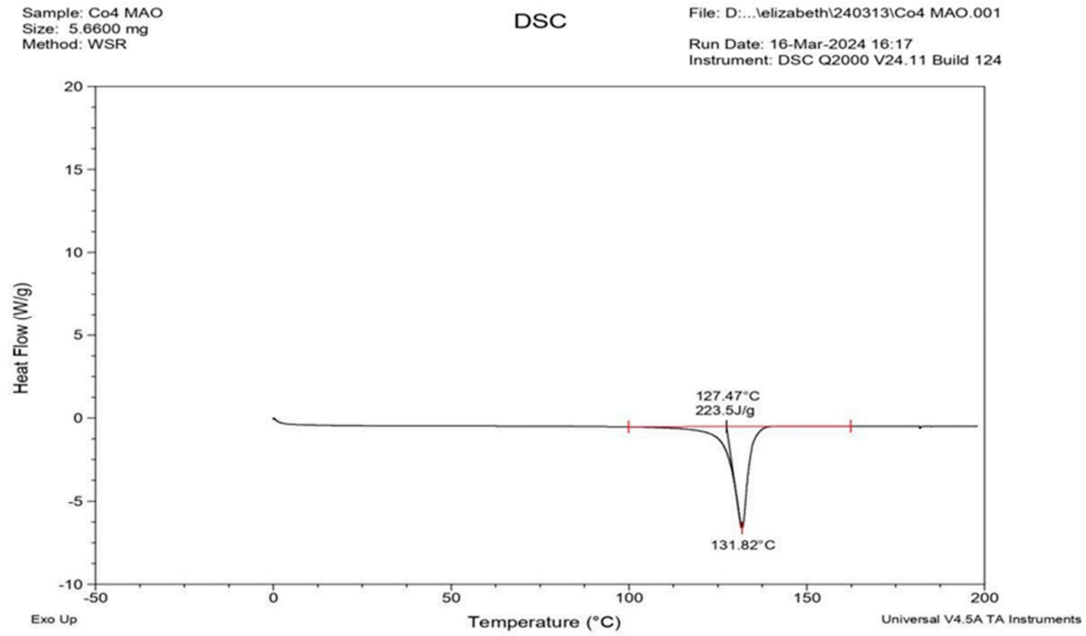

Figure S8-5: DSC curve indicating the T_m of PE prepared by **Co4/MAO** (**Table 2**, entry 9)

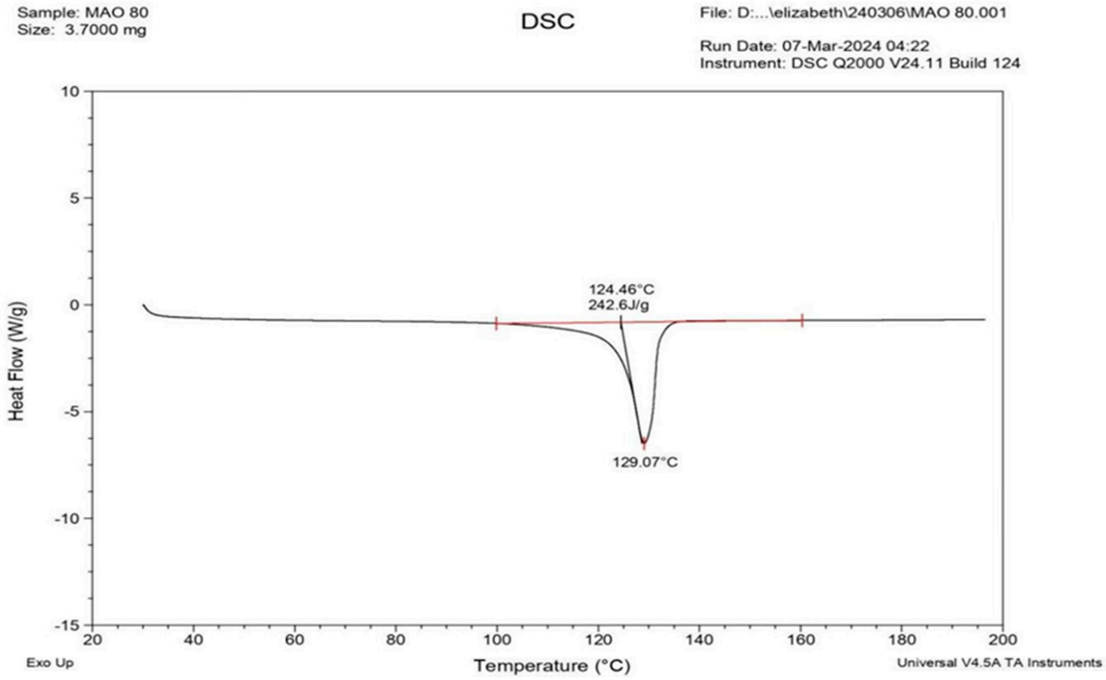

Figure S8-6: DSC curve indicating the T_m of PE prepared by **Co4/MAO** (**Table 2**, entry 10)

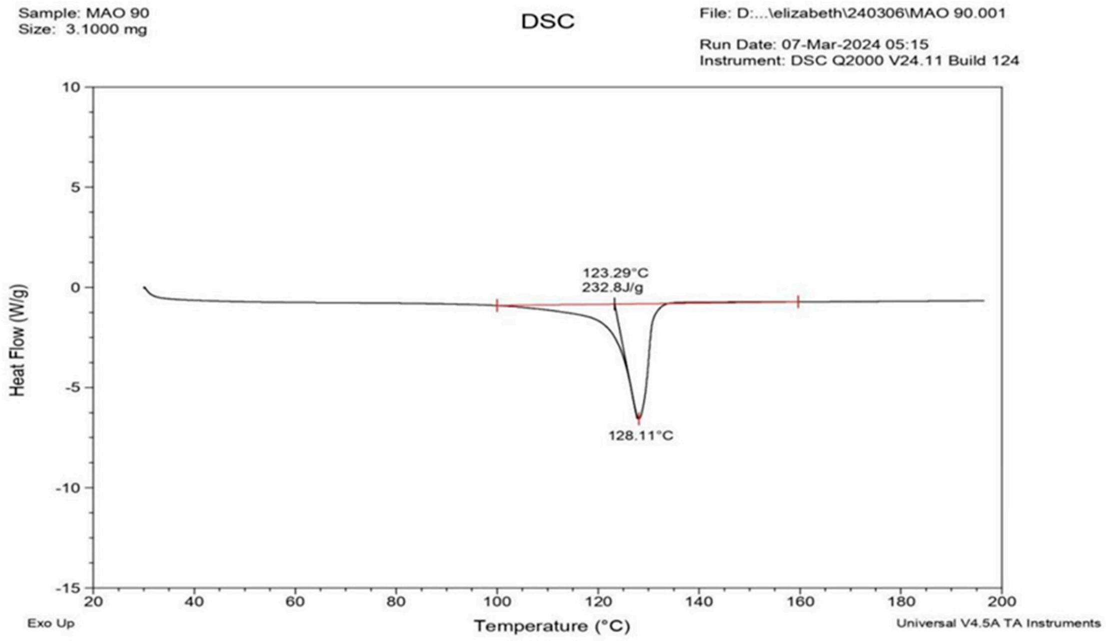

Figure S8-7: DSC curve indicating the *T*m of PE prepared by **Co4/MAO** (**Table 2**, entry 11)

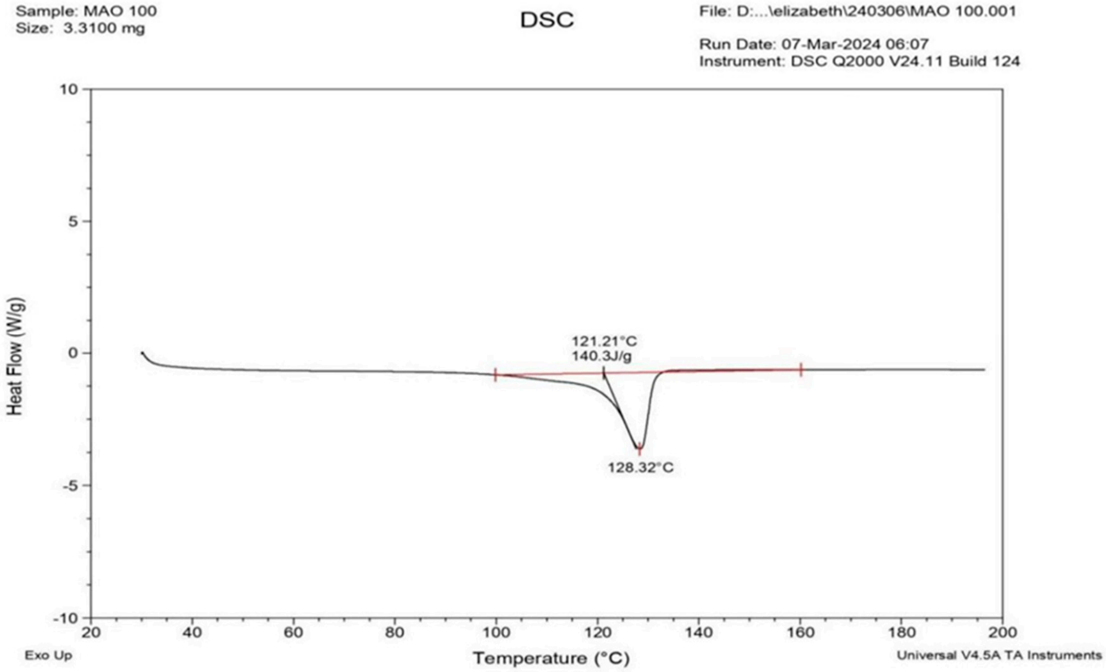

Figure S8-8: DSC curve showing T_m of PE prepared by **Co4/MAO** (**Table 2**, entry 12)

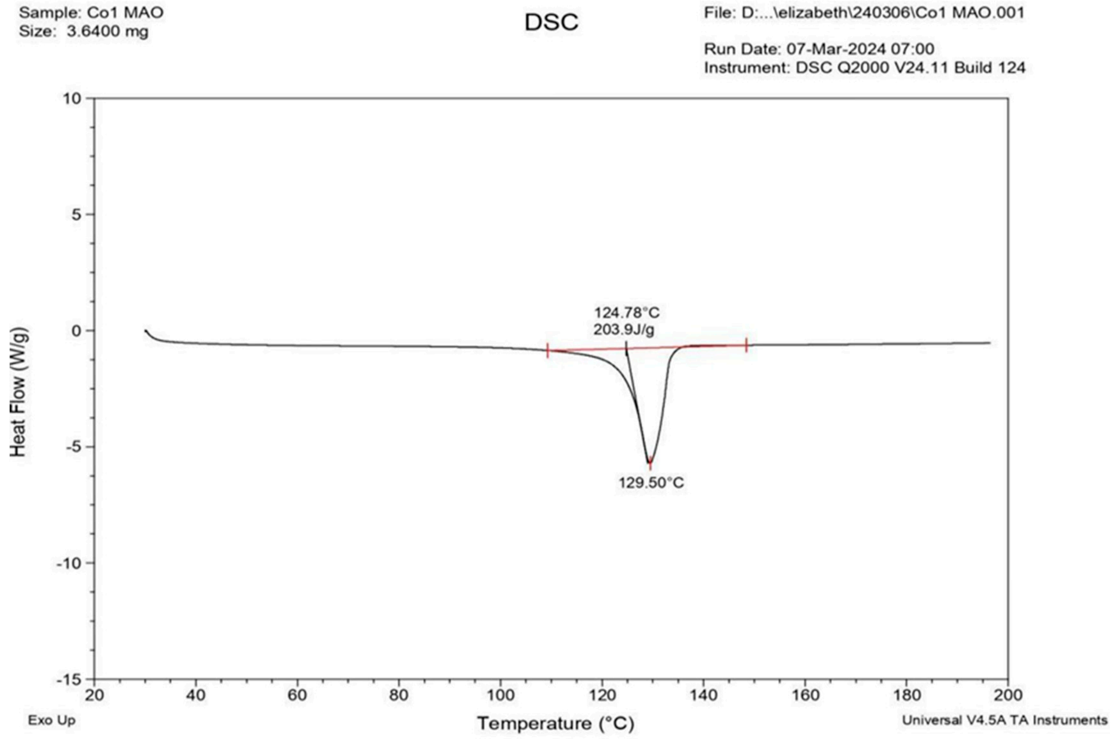

Figure S8-9: DSC curve showing T_m of PE prepared by **Co1/MAO** (**Table 4**, entry 1)

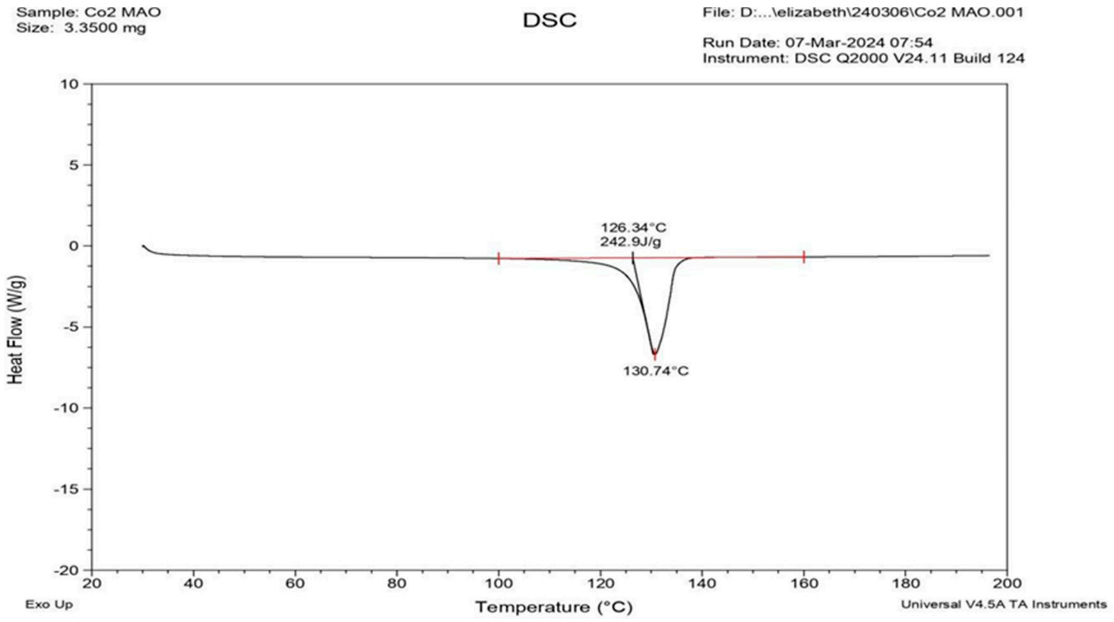

Figure S8-10: DSC curve showing T_m of PE prepared by **Co2/MAO** (**Table 4**, entry 2)

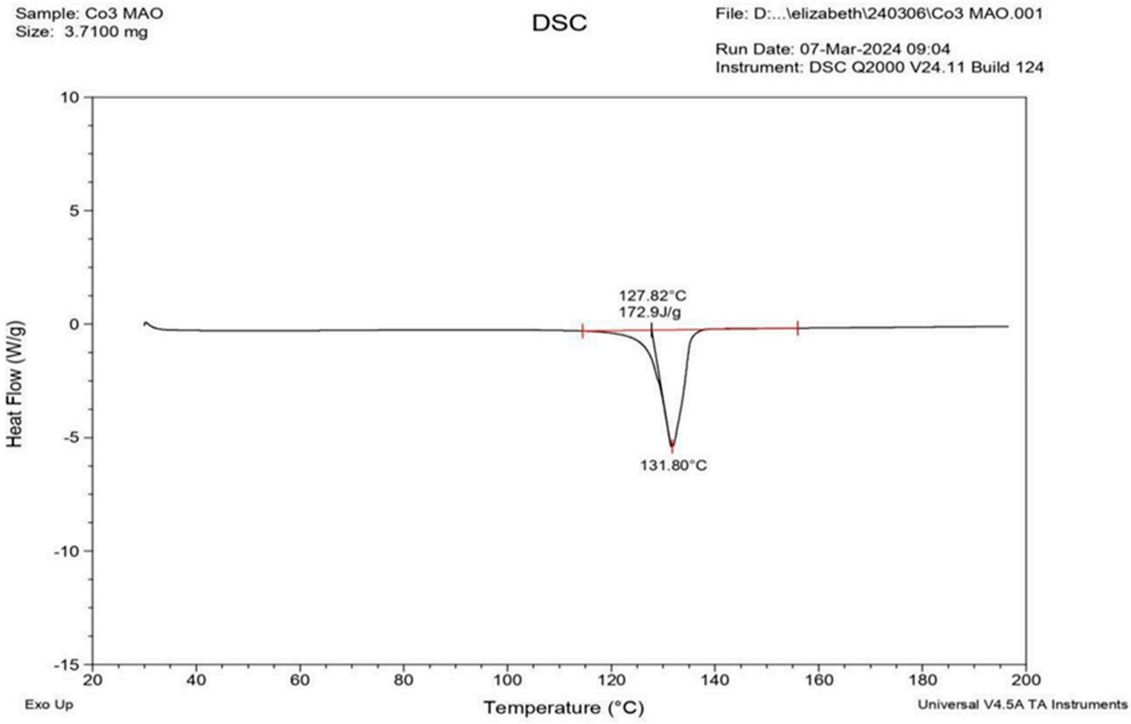

Figure S8-11: DSC curve showing T_m of PE prepared by **Co3/MAO** (**Table 4**, entry 3)

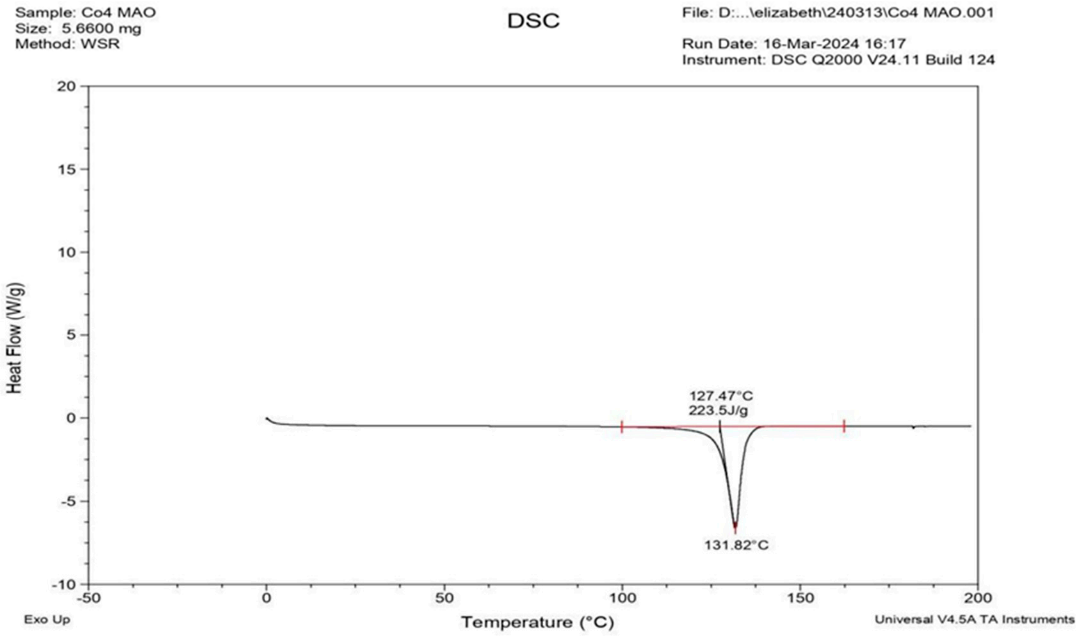

Figure S8-12: DSC curve showing T_m of PE prepared by **Co4/MAO** (**Table 4**, entry 4)

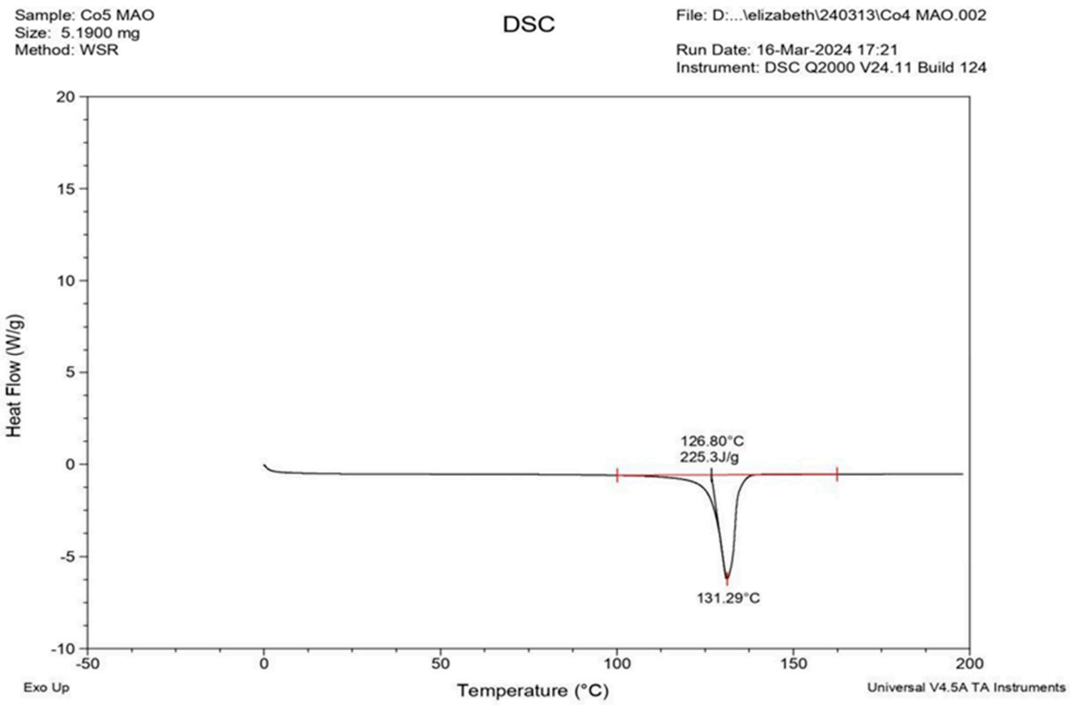

Figure S8-13: DSC curve showing T_m of PE prepared by **Co5/MAO** (**Table 4**, entry 5)

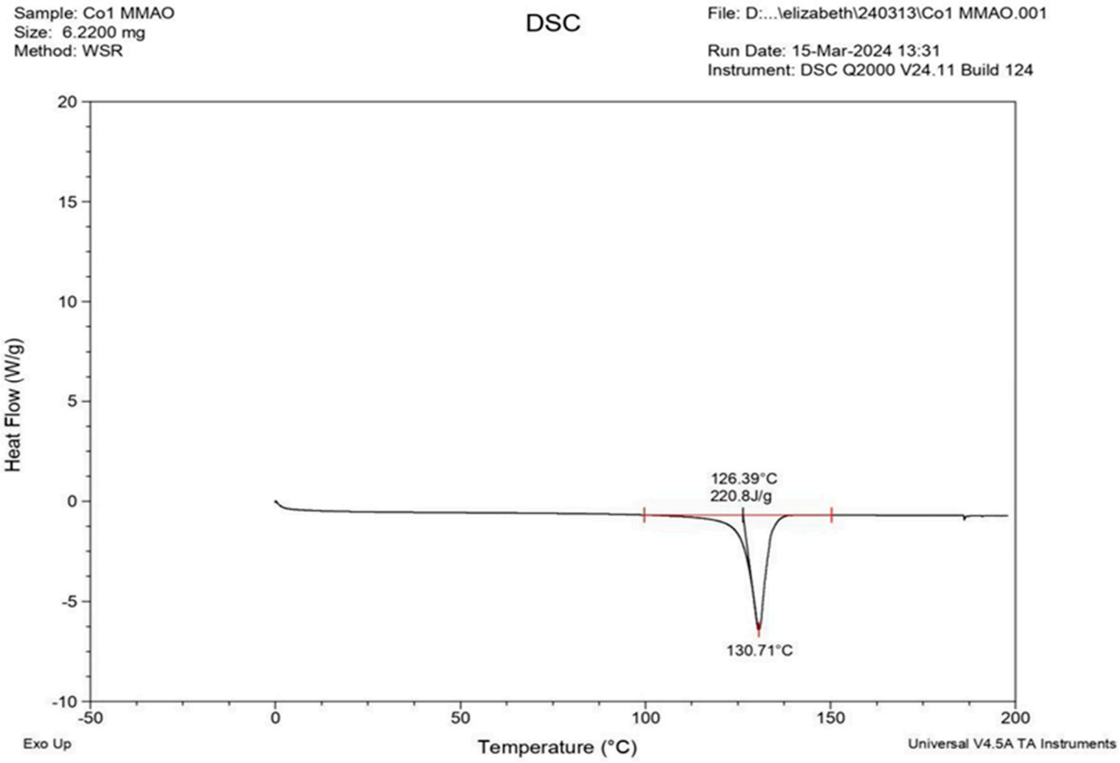

Figure S8-14: DSC curve showing T_m of PE prepared by **Co1/MMAO** (**Table 4**, entry 6)

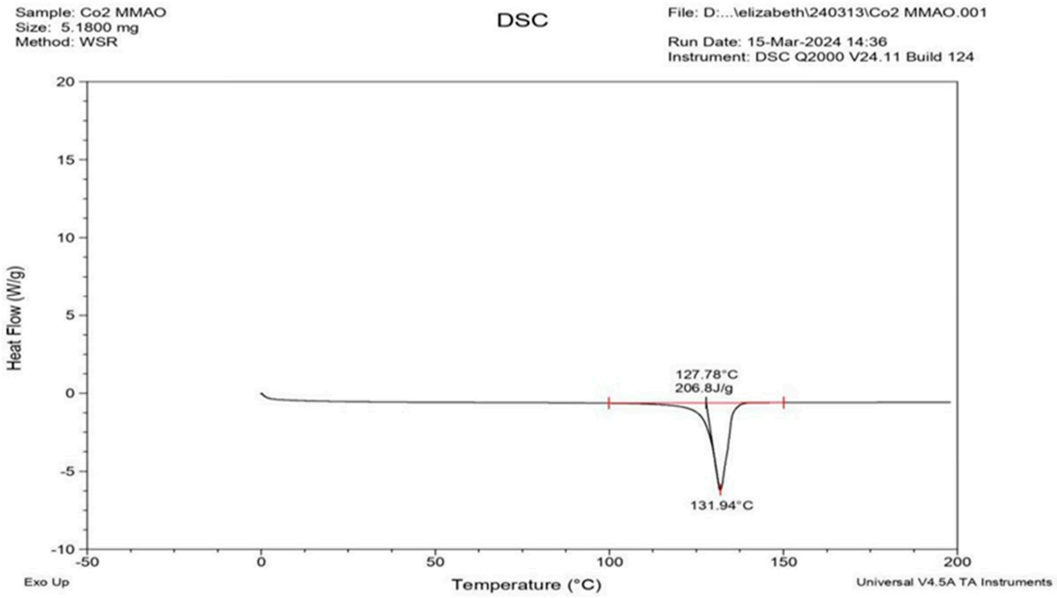

Figure S8-15: DSC curve showing T_m of PE prepared by **Co2/MMAO** (**Table 4**, entry 7)

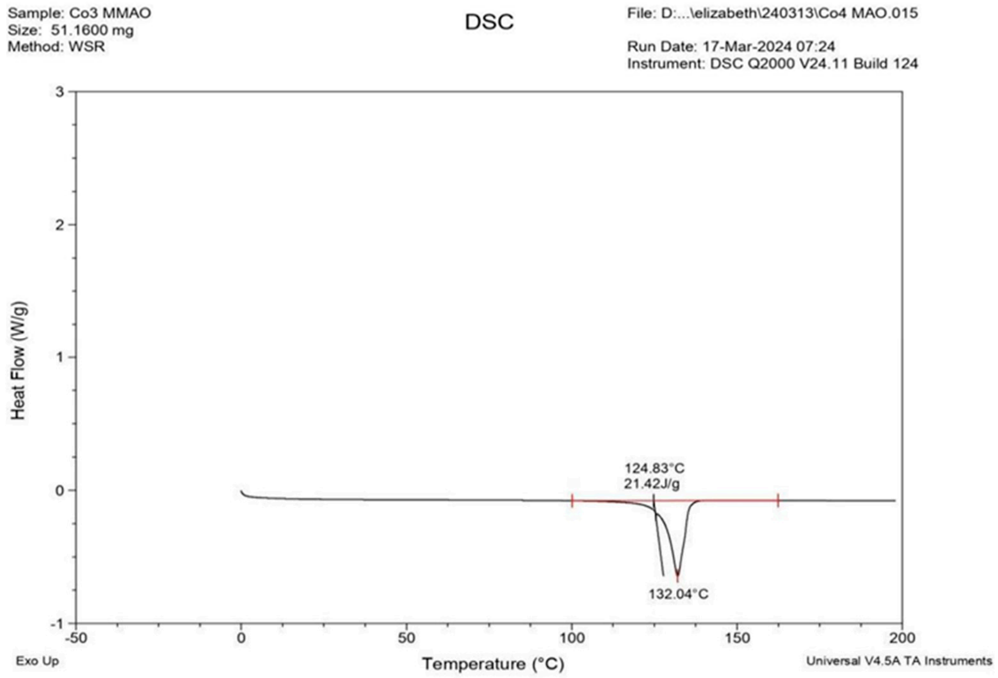

Figure S8-16: DSC curve showing T_m of PE prepared by **Co3/MMAO** (**Table 4**, entry 8)

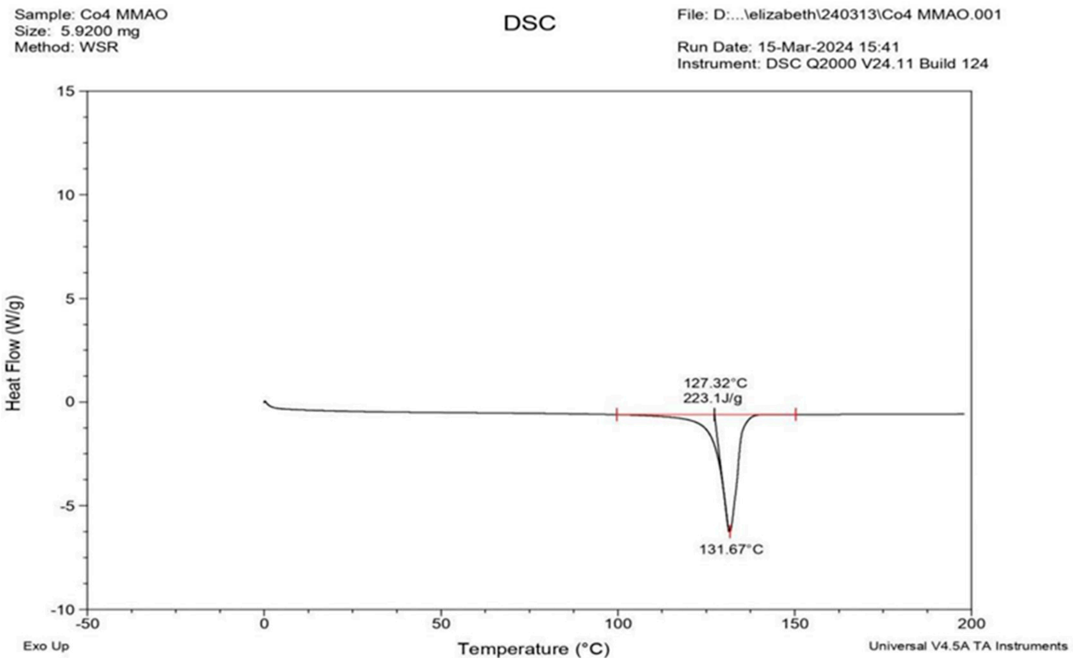

Figure S8-17: DSC curve showing T_m of PE prepared by **Co4/MMAO** (**Table 4**, entry 9)

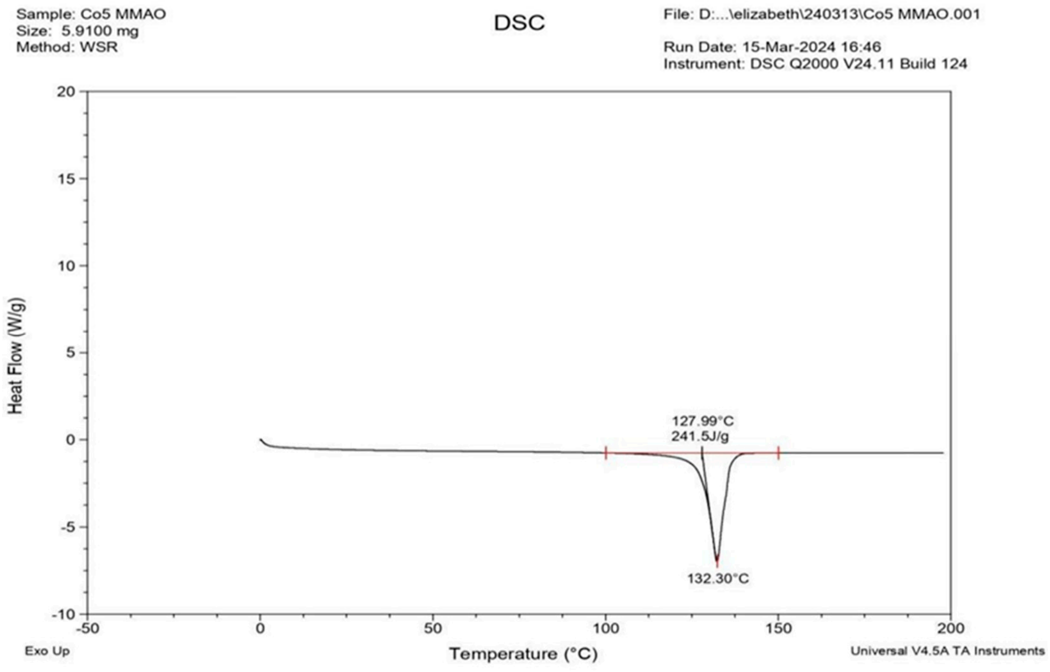

Figure S8-18: DSC curve showing T_m of PE prepared by **Co5/MMAO** (**Table 4**, entry 10)

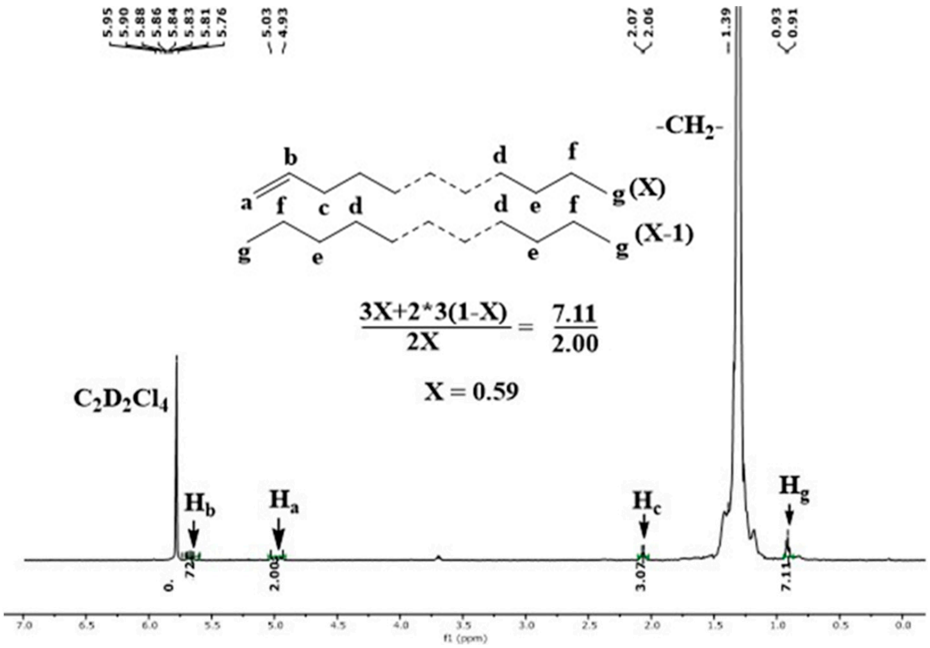

Figure S9: ^{1}H NMR spectrum of polyethylene produced using **Co4/MMAO** (**Table 4** entry 9) was recorded in $C_2D_2Cl_4$ at 100°C

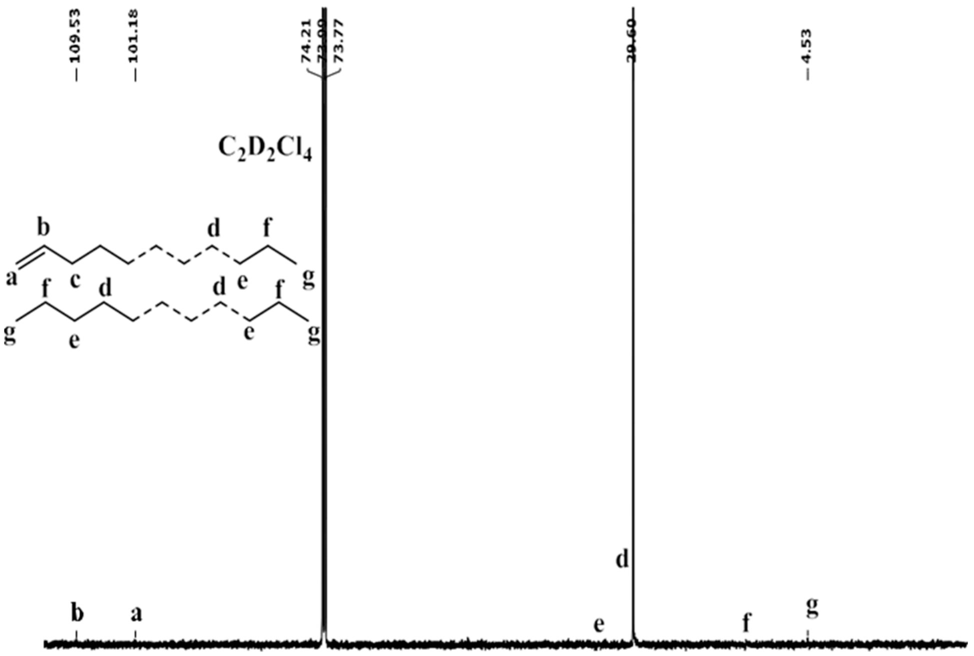

Figure S10: ^{13}C NMR spectrum of polyethylene produced using **Co4/MMAO** (**Table 4,** entry 9) was recorded in $C_2D_2Cl_4$ at 100°C.
